# Benzophenon-3: MAK-Begründung

**DOI:** 10.34865/mb13157d11_2or

**Published:** 2026-06-30

**Authors:** Andrea Hartwig, MAK Commission

**Affiliations:** 1 Institut für Angewandte Biowissenschaften, Abteilung Lebensmittelchemie und Toxikologie, Karlsruher Institut für Technologie (KIT) Adenauerring 20a, Geb. 50.41 76131 Karlsruhe Deutschland; 2 Ständige Senatskommission zur Prüfung gesundheitsschädlicher Arbeitsstoffe, Deutsche Forschungsgemeinschaft, Kennedyallee 40, 53175 Bonn, Deutschland. Weitere Informationen: Ständige Senatskommission zur Prüfung gesundheitsschädlicher Arbeitsstoffe | DFG

**Keywords:** Benzophenon-3, Sensibilisierung, Photosensibilisierung, Kanzerogenität, Meningeome, In-utero-Exposition, Hautresorption, endokrine Wirkungen, Hochdosis

## Abstract

The German Senate Commission for the Investigation of Health Hazards of Chemical Compounds in the Work Area (MAK Commission) summarized and evaluated the data for benzophenone-3 [131-57-7] considering all toxicological end points. Relevant studies were identified from a literature search and also unpublished study reports were used. Benzophenone-3 is not genotoxic. A carcinogenicity study in mice was negative. A special carcinogenicity study was carried out in which rats were exposed in utero beginning on gestation day 6 with continuous exposure of the F1 animals for up to 2 years. Male rats developed meningiomas without statistical significance: in the brain one at the low dose, three at the middle dose and one in the spine at the middle dose. The mid dose animals belonged to two different litters. The mechanism underlying the formation of meningiomas has not been identified, nor is it known whether benzophenone-3 plays any role in this process. As it is unclear whether the in-utero exposure triggered the development of meningiomas in rats, benzophenone-3 has been classified in Category 3 for carcinogens. A MAK value cannot be derived. The data for developmental toxicity and endocrine disruption do not indicate that there is a risk for the unborn child. Benzophenone-3 yielded positive results for hormonal activity in different in vitro assays. By contrast, negative results were obtained for benzophenone-3 in several in vivo studies, such as six uterotrophic assays in rats and mice that tested doses up to 1000 mg/kg body weight (bw) and day for 3 or 4 days. However, one uterotrophic assay yielded positive results in rats at 1525 mg/kg bw and day exposed for 4 days. In a one-generation study in rats, benzophenone-3 did not exhibit estrogenic, androgenic or antiandrogenic activity up to 2700 mg/kg bw and day. Benzophenone-3 has therefore been evaluated as a weak endocrine disruptor at high doses. Benzophenone-3 can provoke contact allergic reactions in humans and has therefore been designated with “Sh”. Light may increase the severity of the contact allergic reactions and may result also in photocontact allergic reactions. Benzophenone-3 penetrates the skin in toxicologically relevant amounts and has therefore been designated with “H”.

**Table:** 

**MAK-Wert**	**–**
**Spitzenbegrenzung**	**–**
	
**Hautresorption (2025)**	**H**
**Sensibilisierende Wirkung (2025)**	**Sh**
**Krebserzeugende Wirkung (2025)**	**Kategorie 3**
**Fruchtschädigende Wirkung**	**–**
**Keimzellmutagene Wirkung**	**–**
	
**BAT-Wert**	**–**
	
	
Synonyma	Oxybenzon
Chemische Bezeichnung (IUPAC-Name)	2-Hydroxy-4-methoxybenzophenon
CAS-Nr.	131-57-7
Formel	Strukturformel von Benzophenon-3 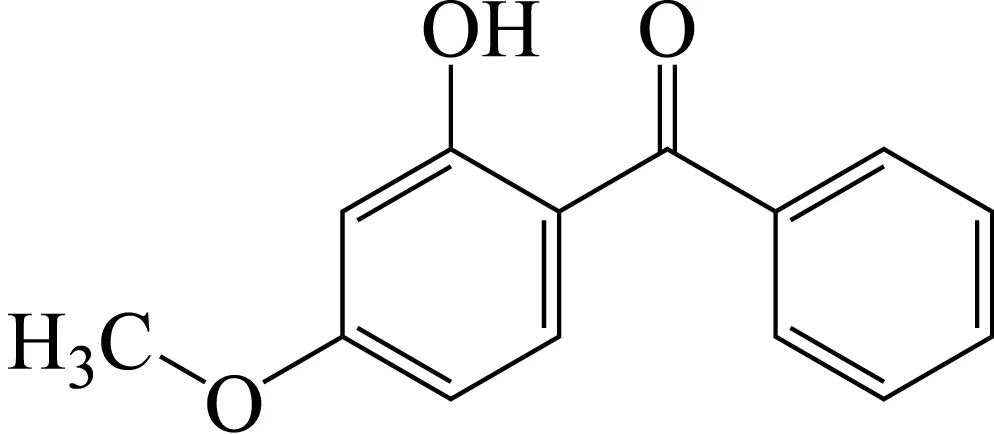
	C_14_H_12_O_3_
Molmasse	228,25 g/mol
Schmelzpunkt	62–65 °C (NTP 2020)
Dampfdruck bei 25 °C	1,1 × 10^–^^5^ hPa (ECHA 2013)
log K_OW_ bei 40 °C	3,45 (ECHA 2013)
Löslichkeit	69 mg/l Wasser bei 25 °C (NTP 2020)
**1 ml/m^3^ (ppm) ≙ 9,471 mg/m^3^**	**1 mg/m^3^ ≙ 0,106 ml/m^3^ (ppm)**
	
Hydrolysestabilität	k. A.
Verwendung	Schutz von Kosmetikprodukten vor (UV)-Licht, als UV-Schutz (Haut), Photoinitiator in UV-Härtungs-Anwendungen (wie Tinten und Beschichtungen in der Druckindustrie), in Farben und Lacken, in (modifizierten) Acrylplastikprodukten kann Kontakt mit Lebensmitteln auftreten (NTP 2020)

Zitierte unveröffentlichte toxikologische Studien von Firmen wurden der Kommission zur Verfügung gestellt.

Benzophenon-3 kommt in Pigmenten von Blumen natürlich vor, ist bei Raumtemperatur ein leicht gelbliches Pulver und wird als UV-Schutz für Haut und Haare in kosmetischen Mitteln eingesetzt. Es absorbiert UVA-Strahlung (320 bis 400 nm) und UVB-Strahlung (290 bis 320 nm) und ist photostabil. Die maximale Einsatzkonzentration in Kosmetikprodukten beträgt bis zu 6 %. Durch den weiten Einsatz von Benzophenon-3 in Kosmetik- und Sonnenschutzprodukten liegt die Hintergrundbelastung im Urin (Kreatinin-adjustierte geometrische Mittelwerte in den USA) bei 9 bis 17 ng/ml Urin bei Männern und 18 bis 45 ng/ml Urin bei Frauen (French 1992; NTP 2020).

## 1 Allgemeiner Wirkungscharakter

Benzophenon-3 wirkt beim Menschen als Kontaktallergen und Photokontaktallergen und kann in seltenen Fällen Kontakturtikaria und kontaktvermittelte, am ehesten als anaphylaktoid zu interpretierende Reaktionen auslösen.

Benzophenon-3 wird von Tieren oral sowie von Mensch und Tier dermal gut resorbiert, Inhalationsdaten fehlen.

In 13-Wochen-Fütterungsstudien ist bei Ratten ab der niedrigsten Dosis von 213 bzw. 196 mg/kg KG und Tag das absolute und relative Lebergewicht um 10–15 % ohne histopathologisches Korrelat erhöht. Bei der höchsten Dosis von 3656 bzw. 3261 mg/kg KG und Tag treten auch Nierenbefunde wie Dilatationen im Tubulus oder Nekrosen in der Papille sowie bei männlichen Tieren eine verminderte Spermienzahl auf. Auch bei Mäusen ist in der 13-Wochen-Fütterungsstudie das Lebergewicht ab 1068 bzw. 1425 mg/kg KG und Tag um 11–16 % ohne weitere histopathologische Befunde erhöht und bei weiblichen Tieren auch das relative Nierengewicht.

In dermalen 13-Wochen-Studien ist bei Mäusen ab der niedrigsten Dosis von 22 mg/kg KG und Tag eine verminderte Spermienzahl in den Nebenhoden aufgetreten. Bei Ratten ist es ab 25 mg/kg KG und Tag zu einem erhöhten relativen Nierengewicht von ca. 10 % ohne histopathologisches Korrelat gekommen.

Benzophenon-3 wirkt nicht mutagen in Bakterien und Säugerzellen. In CHO-Zellen kommt es mit metabolischer Aktivierung zur Induktion von Schwesterchromatidaustauschen und Chromosomenaberrationen. In vivo ergibt Benzophenon-3 in einer Untersuchung auf somatische Mutationen und Rekombinationen in Drosophila ein negatives Ergebnis, der Stoff führt nicht zu Chromosomenaberrationen am Knochenmark männlicher Ratten und nicht zu Mikronuklei in Erythrozyten von Mäusen nach 90-tägiger Gabe mit dem Futter. 

Eine orale Kanzerogenitätsstudie an B6C3F1-Mäusen mit bis zu 1278 mg Benzophenon-3/kg KG und Tag ist negativ. Eine Kanzerogenitätsstudie mit F1-Sprague-Dawley-Ratten und Expositionsbeginn in utero hat nach zwei Jahren bei 58 mg/kg KG und Tag bei einem männlichen Tier und bei 168 mg/kg KG und Tag bei drei männlichen Tieren zu einem Meningeom im Gehirn und bei einem weiteren Tier zu einem im Rückenmark geführt. Bei gleichem Studiendesign mit Expositionsbeginn in utero ist es in zwei weiteren Untersuchungen in diesem Labor im selben Zeitraum auch bei einem männlichen bzw. einem weiblichen Tier der Kontrollgruppe zu einem solchen Tumor im Gehirn gekommen.

Pulverförmiges, angefeuchtetes Benzophenon-3 wirkt nicht reizend an der Haut von Kaninchen und an rekonstituierter Humanhaut in vitro. Am Kaninchenauge wirkt die Substanz als Pulver nicht bis maximal sehr leicht reizend.

In einer Studie zur pränatalen Entwicklungstoxizität an Ratten mit Gavagegabe vom 6. bis zum 19. Gestationstag ist es bei 1000 mg/kg KG und Tag bei gleichzeitiger Maternaltoxizität bei den Feten vermehrt zu skelettalen Variationen, wie nicht vollständige Ossifikationen und zusätzliche 14. Rippen, gekommen. Teratogene Effekte sind nicht aufgetreten.

Benzophenon-3 weist eine schwache endokrine Wirkung bei hoher Dosierung auf.

## 2 Wirkungsmechanismus

### 2.1 Nierentoxizität

Nach oraler Gabe von Benzophenon-3 an Ratten und Mäusen kam es zu Nierentoxizität und bei Ratten auch zu Nekrosen in der Nierenpapille. Der Nekrose voraus gingen Erweiterungen der Nierentubuli und Regeneration der Epithelzellen in den Nieren. Für die durch Chemikalien induzierten Nierenpapillennekrosen werden als Ursachen ein veränderter Blutfluss in der Niere, die Bildung freier Radikale mit direkter Schädigung der Nierenpapillen durch die Chemikalie und eine Hemmung der Prostaglandinsynthese diskutiert. Auch ein reduzierter Urinfluss und der Anstieg gelöster Stoffe im Urin können dazu beitragen. Ob ein Anstieg gelöster Stoffe im Urin nach Benzophenon-3-Gabe der Auslöser der Nierenpapillennekrosen ist, kann nicht geklärt werden. Bei männlichen Ratten tritt jedoch beides in der höchsten Dosisgruppe der oralen 13-Wochen-Studie parallel auf (French 1992).

### 2.2 Meningeome

Benzophenon-3 wirkt nicht genotoxisch (siehe Abschnitt 5.6). In einer 2-Jahre-Studie beginnend mit Exposition in utero traten bei männlichen F1-Sprague-Dawley-Ratten Meningeome auf (NTP 2020; siehe Abschnitt 5.7). Der Entstehungsmechanismus ist unklar. Meningeome sind Tumoren, die aus der Arachnoidea, d. h. dem mittleren Blatt der drei Hirnhäute, ausgehen und beim Menschen ca. 20–30 % aller Tumoren des zentralen Nervensystems ausmachen (Kockro 2025).

#### 2.2.1 Mensch

Beim Menschen kommen Meningeome deutlich häufiger bei Frauen vor, im Durchschnitt aller Altersstufen mit einer doppelten, bis im gebärfähigen Alter dreifachen Häufigkeit im Vergleich zu Männern (Agopiantz et al. 2023). Dies gilt besonders für Meningeome im Rückenmark, bei denen 75–90 % der Fälle bei Frauen beobachtet werden. Es gibt einen Zusammenhang mit endogenen oder exogenen Sexualhormonen, da sich die Größe der Meningeome mit dem Zyklus, beim Auftreten einer Schwangerschaft und in der Menopause verändert (Claus et al. 2008). Der Mechanismus der Wechselwirkung ist jedoch noch unklar (Portet et al. 2020) und zum Einfluss einer Benzophenon-3-Exposition liegen keine Daten vor. Im Gegensatz dazu traten in der Kanzerogenitätsstudie mit Ratten Meningeome nur bei männlichen Tieren mit nicht statistisch signifikanter Inzidenz auf (NTP 2020).

Bei genetischen Untersuchungen von Meningeomen des Menschen zeigte sich, dass ca. 70 % kein Chromosom 22 mehr aufwiesen. Die meisten dieser Meningeome hatten Mutationen im Neurofibromatose-Typ II (*NF2)*-Gen, das auf dem langen Arm des Chromosoms 22 (22q) lokalisiert ist und das Moesin-Ezrin-Radixin like (MERLIN)-Tumorsuppressorprotein codiert. Es wurde geschlossen, dass es sich dabei um ein frühes genetisches Ereignis handeln muss, da der Verlust oder eine Mutation in diesem Gen bei Meningeomen aller Gradierungen auftritt und die häufigste Veränderung bei Meningeomen von Grad 1 ist (Claus et al. 2008). Da es sich um eine Analyse bei manifesten Tumorproben handelt, kann nicht auf einen auslösenden Mechanismus geschlossen werden.

Der hormonelle Status in Meningeomen wurde in 510 humanen Tumorproben untersucht. Dabei zeigte sich, dass Grad-1-Meningeome signifikant mehr Östrogen- und Androgenrezeptoren exprimiert hatten als höhergradige Meningeome. Es traten keine Unterschiede bei der Expression von Sexualhormonrezeptoren zwischen Frauen und Männern oder in verschiedenen Altersgruppen auf. Anhand dieser Daten kann die höhere Inzidenz von Meningeomen bei Frauen nicht erklärt werden. Atypische und maligne Meningeome hatten weniger Östrogen- und Androgenrezeptoren. Östrogenrezeptor-positive Meningeome proliferierten signifikant schneller als Östrogenrezeptor-negative (Korhonen et al. 2006). Da es sich um eine Analyse bei manifesten Tumorproben handelt, kann nicht auf einen auslösenden Mechanismus geschlossen werden.

Die Expression der Hormon-Rezeptoren für Androgene, Östrogene und Progesterone wurde bei 30 intrakranialen und 30 spinalen Meningeomen sowie 30 durch Cyproteronacetat ausgelösten Meningeomen mit Hilfe eines Mikroarrays sowie immunhistochemisch untersucht. Der Androgenrezeptor war in 73 % der intrakranialen Meningeome, 87 % der durch Cyproteronacetat induzierten Meningeome und in allen spinalen Meningeomen exprimiert. Der Östrogenrezeptor war bei 7 % der intrakranialen Meningeome, 3 % der durch Cyproteronacetat induzierten Meningeome und 30 % der spinalen Meningeome exprimiert. Der Progesteronrezeptor war bei 90 % der intrakranialen Meningeome, 97 % der durch Cyproteronacetat induzierten Meningeome und in 87 % der spinalen Meningeome exprimiert. In allen Gruppen korrelierte die Expression von Androgen- und Progesteron-Rezeptoren (Portet et al. 2020). Da es sich um eine Analyse bei manifesten Tumorproben handelt, kann nicht auf einen auslösenden Mechanismus geschlossen werden.

Die Expression von Progesteronrezeptoren nimmt mit zunehmendem Malignitätsgrad ab (Maiuri et al. 2021).

Seit den 1980er Jahren wird eine hormonabhängige Entstehung von Meningeomen diskutiert. Um einen Zusammenhang mit der Expression bestimmter Hormonrezeptoren zu untersuchen, wurde anhand publizierter Literatur ein systematischer Review durchgeführt. Meningeome treten deutlich häufiger bei Frauen auf. Auch scheint eine Assoziation zu Brusttumoren zu bestehen. Die gesunde Hirnhaut exprimiert Progesteronrezeptoren, was nicht bei allen Meningeomen der Fall ist. Die Gabe von Progesteron steigert beim Menschen die Bildung von Meningeomen deutlich: eine Cyproteronacetat-Gabe von sechs Monaten steigert die Inzidenz um das Siebenfache, eine fünfjährige Gabe um das 20-Fache, eine fünfjährige Gabe von Nomegestrolacetat um das Zwölffache. In der Mehrheit der analysierten Literaturstellen zeigte sich eine Expression des Progesteronrezeptors in Meningeomen (bei ca. 75 % der Meningeome, nicht ganz die Hälfte exprimierte den Androgenrezeptor und nur wenige den Östrogenrezeptor (11,3 %)). Der Progesteronrezeptor war in Meningeomen in Abhängigkeit des Geschlechtes (mehr bei Frauen), des Lebensalters (mehr im gebärfähigen Alter), während Schwangerschaft und Stillzeit sowie bei hormoneller Behandlung verstärkt exprimiert. Eine solch klare Abhängigkeit zeigte sich weder beim Östrogen-, noch beim Androgenrezeptor. Einige Studien zeigten eine negative Korrelation zwischen Überlebenszeit der Menschen mit Meningeom und exprimierten Östrogenrezeptoren. Es wurde keine Risikoassoziation mit Medikamenten gefunden, die den Androgenspiegel beeinflussen wie Gonadotropin-Releasing-Hormone (GnRH)-Agonisten oder Antiandrogene. Jedoch war eine Behandlung mit Medikamenten, die SSTR2-abhängig wirkten, wie Somatostatin-Analoga, vorteilhaft, besonders bei inoperablen Tumorlokalisationen. Die meisten der in diesem Review einbezogenen Studien untersuchten benigne Meningeome, wobei die Qualität und Ausrichtung der Studien im Hinblick auf die Fragestellung des Reviews inhomogen war (Agopiantz et al. 2023). Auch in diesem Review fand die Untersuchung an manifesten Tumoren statt, sodass daraus kein Schluss in Bezug auf den Entstehungsmechanismus gezogen werden kann.

#### 2.2.2 Tier

Es wurde das Auftreten von Gliomen wie Meningeomen bei Sprague-Dawley-Kontrollratten in 42 Studien, durchgeführt innerhalb eines Zeitraumes von fünf Jahren, ausgewertet. Männliche Tiere wiesen mit einer Inzidenz von 21/2630 häufiger Meningeome auf als weibliche Tiere mit einer Inzidenz von 9/2765. Das bei Menschen häufige Muster von mulitformen Glioblastomen trat nie auf (Gopinath 1986).

#### 2.2.3 Fazit

Es ist kein Wirkungsmechanismus bekannt, durch den Benzophenon-3 bei Mensch und Tier zu Meningeomen führt. Da Meningeome bei Frauen häufiger sind als bei Männern und sich die Tumorgröße mit dem weiblichen Zyklus innerhalb der Schwangerschaft oder der Menopause verändern kann, ist ein hormoneller Einfluss wahrscheinlich (siehe Abschnitt 2.4). In der Kanzerogenitätsstudie traten Meningeome ausschließlich bei männlichen Ratten nach Expositionsbeginn in utero auf, wobei ein Zusammenhang mit der Benzophenon-3-Exposition unklar ist. Ob diese Tumoren auch bei Expositionsbeginn im Erwachsenenalter beim Tier oder bei Erwachsenen am Arbeitsplatz induziert werden könnten, ist nicht bekannt.

### 2.3 Reproduktionstoxizität

In einem Review von acht epidemiologischen Studien zu weiblichen und fünf Studien zu männlichen Fertilitätsendpunkten wird von Hinweisen auf Störungen des Menstruationszyklus sowie auf ein erhöhtes Risiko für Myome des Uterus und von Endometriose durch Benzophenon-3 berichtet (Mustieles et al. 2023). Die untersuchten Endpunkte, Studiendesigns und Ergebnisse sind sehr unterschiedlich.

Bei Ratten und Mäusen führt die 90-tägige Gabe von Benzophenon-3 mit dem Futter zu verringerten Spermienzahlen (French 1992). In einer In-vitro-Studie an primären Sertolizellen aus Mäusen sind bei der höchsten Konzentration von 100 µM Barrierefunktionsstörungen der Sertolizellen festgestellt worden, jedoch keine Apoptose (Zhang et al. 2022). Daraus lässt sich auf Störungen der Blut-Testes-Schranke als Voraussetzung für die Störungen der Spermatogenese schließen.

Aus In-vitro-Studien und Studien an Mäusen hat sich ergeben, dass Melatonin vor der Benzophenon-3-induzierten Depletion der Oozyten schützt, was die Beteiligung von oxidativem Stress nahelegt (Jin et al. 2020).

Bei Mäusen kommt es nach pränataler oraler und dermaler Exposition in der frühen Phase der Gestation zu fetalen Verlusten. Metabolomanalysen weisen auf Störungen des Metabolismus von Fructose, Mannose, Arginin, Prolin, Pyrimidin und Biotin sowie des Zitronensäurezyklus hin. Dies hat Auswirkungen auf die Steroidgenese, die Proteinsynthese, die Autophagie und die Immunmodulation. Zusammen mit Effekten auf den NO-Haushalt, der Auswirkungen auf die plazentale Thrombose hat, kann es zu fetalen Verlusten kommen (Han et al. 2022).

Es wurde untersucht, ob Benzophenon-3 in vitro oder intrauterin an Mäuse verabreicht die Brustdrüsenentwicklung weiblicher Tiere beeinflusst. Dafür erhielten trächtige Tiere vom 8. bis 18. Trächtigkeitstag 0; 0,15 oder 50 mg Benzophenon-3/kg KG und Tag in Sesamöl auf die Haut appliziert und es wurden die Brustdrüsen der Nachkommen am 45. Lebenstag untersucht. Benzophenon-3 hemmte bei den in utero exponierten Tieren die mRNA-Menge des Progesteronrezeptors und die mRNA-Menge des WNT4, ohne zu einer Veränderung der Brustdrüsenentwicklung zu führen (Schierano-Marotti et al. 2024).

### 2.4 Endokrine Wirkung

#### 2.4.1 In vitro

In Screening-Assays im Rahmen des Tox21-Programms zeigte sich eine Aktivität von **Benzophenon-3** bei der Stimulierung von Östrogenrezeptoren, Progesteronrezeptoren, Bestandteilen der Androstanrezeptoren, Pregnan-X-Rezeptoren, Retinsäurerezeptoren und Östrogen-abhängigen Signalwegen. Benzophenon-3 hemmte den Androgenrezeptor-Signalweg (NTP 2020).

Der Metabolit** 2,4-Dihydroxybenzophenon** (Benzophenon-1) zeigte in Screening-Assays im Rahmen des ToxCast-Programms in 26 von 42 Untersuchungen eine geringe östrogene Aktivität (gering: Faktor 100–1 000 000 schwächer als die Positivkontrolle), zur antiöstrogenen Wirkung konnte keine Aussage getroffen werden (zu wenige Untersuchungen). Eine androgene Wirkung trat nicht auf und die antiandrogene Wirkung war sehr gering. Es zeigte sich eine geringe inhibierende Wirkung auf die Schilddrüsenperoxidase im Hochdurchsatz-Screening, aber keine Wirkung auf die Aromatisierung von Androgenen oder Östrogenen (SCCS 2025).

**Benzophenon-3** band an und aktivierte den Östrogenrezeptor alpha (ERα) mit einer IC_50_ von 3–20 µM und kann auch ERβ aktivieren (NTP 2020) mit höherer Affinität zu ERα (SCCS 2025). Möglicherweise kann Benzophenon-3 auch als ERα-, ERβ- und Progesteron-Antagonist wirken. In spezifischen Untersuchungen zur ER-Bindung lag die gebundene Menge von Benzophenon-3 jedoch unterhalb von 75 %, welches als „keine Wechselwirkung“ bewertet wird. In einem Luciferase-Assay zeigte Benzophenon-3 eine schwache Antwort und konnte die Proliferation von MCF7-Zellen stimulieren (östrogenartige Wirkung) (NTP 2020).

In vitro hatte **2,4-Dihydroxybenzophenon** eine 5- bis 5000-fach geringere östrogene Aktivität als Standard-Agonisten. Die EC_50_-Werte in Östrogenrezeptor-Agonisten-Tests lagen zwischen 1,15 und 86 µM. Ebenso zeigte 2,4-Dihydroxybenzophenon keine eindeutigen Hinweise auf eine Bindung an den Androgenrezeptor der Ratte und wies eine schwache antiandrogene Aktivität über die Transkriptionsaktivierung auf (IC_50_ im Bereich von 0,69 bis > 100 µM), dies ist in etwa 4- bis mehr als 100-mal weniger stark als die bekannten antagonistischen Referenzsubstanzen (z. B. Hydroxyflutamid). Außerdem veränderte 2,4-Dihydroxybenzophenon drei Gene, die an der zentralen Regulierung des Schilddrüsensystems beteiligt sind, wobei es im Vergleich zum Standard-Agonisten T3 mehr als 10 000-mal weniger wirksam war. 2,4-Dihydroxybenzophenon hatte keine Auswirkungen auf die Steroidgenese (SCCS 2025).

In der humanen Brustzelllinie MCF-7 betrug die EC_50_ für östrogene Aktivität von Benzophenon-3 19,5 µM und die von 2,4-Dihydroxybenzophenon 1,26 µM. Die antiandrogene Aktivität (IC_50_) lag in NIH3T3-Zellen, die mit einem Luciferasetransportergen transfiziert waren, für Benzophenon-3 bei > 100 µM und für 2,4-Dihydroxybenzophenon bei 10 µM (Suzuki et al. 2005). In diesen Untersuchungen war der Metabolit also gut 10-fach wirksamer als Benzophenon-3.

In einem kompetitiven Reporter-Gen-Bindungstest mit den humanen Östrogenrezeptoren hERα und hERβ betrugen die EC_50_-Werte (halb-maximale Luciferaseaktivität) bei hERα > 30 µM für Benzophenon-3 und 8,5 µM für 2,4-Dihydroxybenzophenon. Bei hERβ war Benzophenon-3 ohne Effekt und der Wert für 2,4-Dihydroxybenzophenon lag bei 3,9 µM (Molina-Molina et al. 2008). Auch in dieser Untersuchung war der Metabolit aktiver als Benzophenon-3.

Eine erhöhte Anfälligkeit gegen oxidativen Stress und Zytotoxizität zeigte sich in Rattenthymozyten, die gegen Benzophenon-3 exponiert waren (Wnuk et al. 2022).

Insgesamt war in den In-vitro-Untersuchungen der Hauptmetabolit 2,4-Dihydroxybenzophenon ähnlich oder etwas stärker wirksam als Benzophenon-3.

#### 2.4.2 In vivo

##### 2.4.2.1 Humandaten

Bei Männern zeigte sich eine Assoziation zwischen der Benzophenon-3-Konzentration im Urin und einem signifikant niedrigeren Testosterongehalt im Serum. Bei schwangeren Frauen wurde hingegen keine Assoziation zwischen der Benzo­phenon-3-Konzentration im Urin und den Gehalten an Östrogen, Progesteron, luteinisierendem Hormon, follikelstimulierendem Hormon und Geschlechtshormon-bindendem Globulin gefunden. Um Aussagen zu einer möglichen endokrinen Wirkung zu treffen, wurden Benzophenon-3-Konzentrationen im Urin mit dem Eintritt der Geschlechtsreife bei Jugendlichen verglichen. Die Ergebnisse waren uneindeutig, da sich bei einer Untersuchung eine Assoziation der Benzophenon-3-Konzentration im Urin mit einem früheren Einsetzen der Regelblutung zeigte, in einer anderen Untersuchung jedoch die Brustentwicklung bei Mädchen verzögerte. Eine weitere Untersuchung zeigte keine Assoziation zwischen der Urinkonzentration von Benzophenon-3 bei schwangeren Frauen und dem Zeitpunkt der Pubertät der Nachkommen. Uneindeutig waren auch die Ergebnisse verschiedener Untersuchungen zur Wirkung von Benzophenon-3 auf die Hypothalamus-Hypophysen-Schilddrüsenachse. In einer Untersuchung führte ein Anstieg von Benzophenon-3 im Urin schwangerer Frauen zu einer 3%igen Abnahme von freiem T3 im Blut, wobei Thyreoid-stimulierendes Hormon (TSH) und T4 konstant blieben. Auch in der Allgemeinbevölkerung der USA war Benzophenon-3 bestimmend für verminderte Konzentrationen an Schilddrüsenhormonen. Dem gegenüber stehen andere Untersuchungen, die bei Nachweis von Benzophenon-3 im mütterlichen Urin keine veränderten T3- oder T4-Spiegel gefunden haben (Wnuk et al. 2022).

An 15 jungen Männern und 17 Frauen in der Postmenopause wurde die Wirkung einer viertägigen dermalen Applikation von Sonnencreme (Formulierung mit 10 % Benzophenon-3, G/G; 2 mg Benzophenon-3/cm^2^) auf die Hypothalamus-Hypophysen-Schilddrüsenachse in einer zweiwöchigen, einfach verblindeten Studie untersucht. In der ersten Woche wurde eine Basiscreme, in der zweiten Woche vier Tage lang eine Sonnencreme mit Benzophenon-3 am ganzen Körper aufgetragen. Die täglich applizierte Menge betrug bei Männern 40 g, bei Frauen 35 g. Alle Probanden waren gesund und nahmen keine Medikamente. Der Hormonstatus wurde vier Wochen vor und während der Studie untersucht. Es trat kein signifikanter Effekt auf die Spiegel von Thyroxin-bindendem Globulin (TGB), TSH, Gesamt-T4, freiem T4, Gesamt T3 und freiem T3 auf. Das resorbierte Benzophenon-3 erreichte eine maximale mediane Plasmakonzentration von 187 ng/ml bei Frauen nach vier Stunden und 238 ng/ml bei Männern nach drei Stunden. Im Urin wurden maximale Konzentrationen von 44 ng/ml bei Frauen nach 48 Stunden und 81 ng/ml bei Männern nach 24 Stunden gefunden (Janjua et al. 2007, 2008).

##### 2.4.2.2 Tierdaten

Benzophenon-3 wurde im Hershberger-Test in Anlehnung an OECD-Prüfrichtlinie 441 an kastrierten aber mit Testosteron-Propionat substituierten (0,4 mg/kg KG und Tag) männlichen Wistar-Ratten auf eine antiandrogene Wirkung untersucht. Statt drei, wie in der Prüfrichtlinie vorgeschrieben, wurden nur zwei Testgruppen und keine Positivkontrolle eingesetzt. Benzophenon-3 wurde in Maiskeimöl verabreicht. Die Ratten erhielten zehn Tage lang 5 ml Benzophenon-3-Lösung/kg KG und Tag mit der Schlundsonde und 0,5 ml Testosteron-Propional/kg KG und Tag subkutan. Die orale Dosis betrug 300 oder 1000 mg Benzophenon-3/kg KG und Tag, die an je sechs Tiere pro Dosisgruppe verabreicht wurde. Unter diesen Testbedingungen zeigte sich keine antiandrogene Wirkung bei klinischen und hormonellen oder histopathologischen Untersuchungen (ECHA 2013). Im Gegensatz dazu zeigte Benzophenon-3 im Hershberger-Test nach OECD-Prüfrichtlinie 441 nach zehntägiger oraler Applikation mit der Schlundsonde in Maiskeimöl an kastrierte männliche Sprague-Dawley-Ratten ab der niedrigsten Dosis von 40 mg/kg KG und Tag antiandrogene Effekte (Sung et al. 2024). Da der erste Test mit Abweichungen von der Prüfrichtlinie und ohne Positivkontrolle durchgeführt wurde, kann er zur Bewertung nicht herangezogen werden. Der zweite Test nach OECD-Prüfrichtlinie ist als valide zu betrachten.

Ein Uterotrophie-Test an nicht geschlechtsreifen Ratten wurde in Anlehnung an OECD-Prüfrichtlinie 440 durchgeführt. Als Abweichung zur Prüfrichtlinie wurde an vier statt an drei aufeinanderfolgenden Tagen mit der Schlundsonde appliziert, die Dosierung war am 25. Lebenstag beendet statt davor, es erfolgten täglich zweimal 5-ml/kg-KG-Applikationen (einmal morgens, einmal abends) statt nur einmal 5 ml/kg KG. Außerdem wurde nicht zwischen Feuchtgewicht und blutigem Uterusgewicht unterschieden, es lagen keine Angaben zur Stabilität vor, als Positivkontrolle wurde Diethylstilbestrol statt Ethinylestradiol verwendet und keine Negativkontrolle mitgeführt. Benzophenon-3 in Dosierungen von 500 und 1000 mg/kg KG und Tag in Sesamöl induzierte kein Uteruswachstum und zeigte somit unter diesen Testbedingungen keine östrogene Wirkung, die Positivkontrolle hingegen schon (ECHA 2013).

Es wurden sechs Uterotrophie-Tests an nicht geschlechtsreifen Mäusen und Ratten mit Dosierungen von 3 bis 1000 mg Benzophenon-3/kg KG und Tag durchgeführt, die alle negativ verliefen und keinen signifikanten Effekt auf das Uterusgewicht zeigten: BALB/c-Mäuse, vier Tage, 3 mg/kg KG und Tag, Schlundsonde bzw. Pipette (LaPlante et al. 2018; Majhi et al. 2020); Sprague-Dawley-Ratten, drei Tage, 320 oder 1000 mg/kg KG und Tag, Schlundsonde (NTP 2020); C57BL/6J-Mäuse, sieben Tage, 30–1000 mg/kg KG und Tag, oral oder subkutan (Ohta et al. 2012); Sprague-Dawley-Ratten, fünf Tage, 250 oder 1000 mg/kg KG und Tag, Schlundsonde (Schlecht et al. 2004); F344-Ratten, drei Tage, 20–500 mg/kg KG und Tag, intraperitoneal (Suzuki et al. 2005). Ein weiterer Assay ergab bei der höchsten Dosierung von 1525 mg/kg KG und Tag nach viertägiger Applikation per Futter einen signifikanten 23%igen Anstieg des Uterusgewichtes bei Long-Evans-Ratten (LOAEL, NOAEL = 937 mg/kg KG und Tag) (Schlumpf et al. 2001).

Benzophenon-3 zeigte in der im Abschnitt 5.5.1 beschriebenen Ein-Generationenstudie an Sprague-Dawley-Ratten bis zur höchsten Dosis von 2644/2760 mg/kg KG und Tag weder Veränderungen der Androgen-vermittelten Entwicklungsmarker noch histopathologische Veränderungen, die auf eine Störung der normalen Östrogen- oder Androgenrezeptor-vermittelten Entwicklung hindeuten. Es traten auch keine Veränderungen bei Östrogen- oder Androgenrezeptor-abhängigen Prozessen und keine Wirkung auf Verpaarung oder Schwangerschaftsindizes auf (NTP 2022 a).

In einer Untersuchung an trächtigen Ratten führte Benzophenon-3 nach dermaler Gabe zu höheren T3- und T4- und verminderten TSH-Spiegeln, was zu Hyperthyreose führte. Benzophenon-3 unterbrach die Hypothalamus-Hypophysen-Schilddrüsenachse z. B. bei Ratten, da es als Agonist des Thyroid-Rezeptors fungierte und dadurch die Expression des Östrogenrezeptors ERR1 verminderte (Wnuk et al. 2022).

Mit dem Metaboliten **2,4-Dihydroxybenzophenon** wurden sechs Uterotrophie-Tests an Nagetieren durchgeführt. Alle Versuche zeigten eine schwache uterotrophe (östrogene) Wirkung bei Ratten und Mäusen. Der orale (Schlundsonde) NOAEL für östrogene Wirkungen bei Mäusen wurde mit 300 mg/kg KG und Tag angegeben, der LOAEL betrug 1000 mg/kg KG und Tag. Der NOAEL bei subkutaner Verabreichung lag bei 100, der LOAEL bei 300 mg/kg KG und Tag (Ohta et al. 2012). Mit Ratten wurde bei oraler Gabe kein NOAEL erhalten, ab 100 mg/kg KG und Tag trat erhöhtes Uterusgewicht auf (Yamasaki et al. 2004). Bei subkutaner Verabreichung lag der NOAEL bei 100 mg/kg KG und Tag, der LOAEL bei 625 mg/kg KG und Tag (Koda et al. 2005). Der NOAEL bei intraperitonealer Verabreichung betrug für östrogene Wirkungen 100, der LOAEL 500 mg/kg KG und Tag (Suzuki et al. 2005). In einem Uterotrophie-Test an Ratten lagen die niedrigsten effektiven Dosen für östrogene bzw. antiöstrogene Wirkungen bei log 2,67 bzw. log 3,15 µmol/kg KG und Tag (nach Angabe der Autoren entsprechend 0,553 oder 0,652 mg/kg KG und Tag nach Division durch den Umrechnungsfaktor 4,83) (SCCS 2025). Somit wirkt der Metabolit etwas stärker uterotroph als Benzophenon-3.

#### 2.4.3 Fazit

In vitro wurde die Stimulation einiger Hormonrezeptoren durch Benzophenon-3 nachgewiesen (Molina-Molina et al. 2008; NTP 2020; SCCS 2025; Suzuki et al. 2005). Bei Ratten und Mäusen führte Benzophenon-3 in vivo bis zu einer Dosis von 1000 mg/kg KG und Tag im Uterotrophie-Assay zu keinem Effekt (ECHA 2013; NTP 2020; Ohta et al. 2012; Schlecht et al. 2004). Eine schwach östrogene Wirkung zeigt sich aufgrund des mit einer höheren Dosis von 1525 mg/kg KG und Tag positiven Uterotrophie-Tests an ovariektomierten Ratten, der NOAEL in dieser Untersuchung lag bei 937 mg/kg KG und Tag (Schlumpf et al. 2001). 

In einer Ein-Generationenstudie an Sprague-Dawley-Ratten verursachte Benzophenon-3 bis zur höchsten Dosis von 2644/2760 mg/kg KG und Tag keine Veränderungen bei Östrogen- oder Androgenrezeptor-abhängigen Prozessen und zeigte keinen Einfluss auf Verpaarung oder Schwangerschaftsindizes (NTP 2022 a).

In den vorliegenden Humandaten mit Benzophenon-3 wurden zum Teil Assoziationen zu bestimmten endokrinen Endpunkten berichtet, die jedoch in weiteren Untersuchungen nicht auftraten oder zu einem gegenläufigen Ergebnis führten. In einer zweiwöchigen, einfach verblindeten Studie zeigten sich keine Effekte auf die Hypothalamus-Hypophysen-Schilddrüsenachse (Wnuk et al. 2022).

Der Metabolit 2,4-Dihydroxybenzophenon hat eine etwas stärkere endokrine Wirkung als das Benzophenon-3. Beide Substanzen weisen im Vergleich zu Positivsubstanzen jedoch nur eine geringe endokrine Wirkung bei hoher Dosierung auf (Koda et al. 2005; Ohta et al. 2012; SCCS 2025; Suzuki et al. 2005; Yamasaki et al. 2004).

### 2.5 UV-Reaktivität

Benzophenon-3 absorbiert ein breites Spektrum von UVA-, UVB- und UVC-Strahlung und wird in Kosmetikprodukten zum Schutz vor UV-Strahlung bzw. in Sonnencremes als UV-Schutz der Haut eingesetzt (Wnuk et al. 2022). Benzophenon-3 ist photostabil bei Bestrahlung mit UVA (320 bis 400 nm) und UVB (290 bis 320 nm) in einer Dosis von 100 J UVA pro cm^2^ (Menge, die in Skandinavien an einem ganzen Tag die Erdoberfläche erreicht) bzw. 20 minimalen Erythem-Dosen (MED) von UVB (maximale Dosis während eines Tages in Äquatornähe). Nach Angaben der Autoren bietet ein Sonnenschutzmittel, das unter diesen Bedingungen stabil ist, einen ausreichenden UVB-Schutz (Tarras-Wahlberg et al. 1999).

In einer In-vitro-Studie reduzierte Benzophenon-3 UV-induzierte Radikalreaktionen und besitzt somit antioxidative Eigenschaften in einer Fibroblasten-Zelllinie. Ein vermuteter Mechanismus dafür ist eine Keto-Enol-Tautomerisierung. Obwohl Benzophenon-3 diese Eigenschaften besitzt und somit die Haut vor UV-Schäden schützt, gibt es Hinweise darauf, dass Sonnenschutzmittel durch UV-Strahlung Abbauprozesse durchlaufen, bei denen freie Radikale und andere reaktive oder toxische Reaktionsprodukte entstehen, die mit DNA, Lipiden und Proteinen reagieren können. Beispielsweise führte UV-Strahlung (k. w. A.) oder Sonnenlichtexposition von gegen Benzophenon-3 exponierten HaCaT-Zellen zu reaktiven Sauerstoffspezies, erhöhter Lipidperoxidation, vermindertem Membranpotential von Mitochondrien, Freisetzung von apoptoseinduzierenden Proteinen und Aktivierung von Caspase-3. Die Autoren dieser Studie diskutieren diese Ergebnisse im Zusammenhang mit dem phototoxischen Potenzial von Benzophenonen in der HaCaT-Zelllinie (Wnuk et al. 2022). Im Gegensatz zu diesen Ergebnissen wurden bisher keine phototoxischen Effekte durch Benzophenon-3-Abbauprodukte nach UV-Exposition festgestellt (siehe Abschnitt 4.3 und 5.1).

**Fazit**: Benzophenon-3 zeigt keine phototoxische Wirkung.

## 3 Toxikokinetik und Metabolismus

### 3.1 Aufnahme, Verteilung, Ausscheidung

#### 3.1.1 Inhalative Aufnahme

Hierzu liegen keine Daten vor.

#### 3.1.2 Orale Aufnahme

##### 3.1.2.1 Mensch

Hierzu liegen keine Daten vor.

##### 3.1.2.2 Tier

Nach einmaliger Schlundsonden-Gabe von 100 mg Benzophenon-3/kg KG an männliche Sprague-Dawley-**Ratten** wurde T_max_ nach drei Stunden erreicht, C_max_ betrug 21,21 µg/ml Blut. Die Elimination aus dem Blut verlief biphasisch mit Halbwertszeiten von 0,88 und 15,9 Stunden (NTP 2020). Nach Resorption verteilt sich Benzophenon-3 mit dem Blut in die Organe und kann möglicherweise die Blut-Hirn-Schranke passieren (Wnuk et al. 2022). In den untersuchten Geweben war die Konzentration in der Leber am höchsten (k. w. A.) mit einem Maximalwert nach sechs Stunden (k. w. A.). In einer anderen Untersuchung an männlichen Sprague-Dawley-Ratten und Schlundsonden-Gabe von 100 mg/kg KG betrug T_max_ 2,72 Stunden, C_max_ 21,21 µg/ml und die Plasma-Halbwertszeit lag bei 4,58 Stunden (NTP 2020).

Wurde 10 mg/kg KG an männliche und weibliche Harlan Sprague-Dawley-Ratten mit der Schlundsonde verabreicht, wurde eine T_max_ von 6,0 Stunden und eine C_max_ von 8,5 ng/ml für männliche und 2,3 Stunden und 2,9 ng/ml für weibliche Tiere bestimmt. Die Elimination aus dem Plasma wies eine Halbwertszeit von 6,4 Stunden für männliche und 18,5 Stunden für weibliche Tiere auf (Mutlu et al. 2020).

Bei männlichen und weiblichen Sprague-Dawley-Ratten wurden nach einmaliger Schlundsonden-Gabe von 10, 100 und 500 mg ^14^C-Benzophenon-3/kg KG Radioaktivität in dosisabhängiger Menge in den verschiedenen Geweben nachgewiesen. Die insgesamt wiedergefundene Radioaktivität aller untersuchten Gewebe betrug bei 100 mg/kg KG nach 2, 24 oder 72 Stunden 27,5 %, 3,1 % und < 0,5 % der applizierten Radioaktivität. Dies zeigte eine Verteilung, aber keine Anreicherung in den Geweben an. Innerhalb von 72 Stunden wurden bei allen Dosierungen 53 % bis 58 % der resorbierten Radioaktivität mit dem Urin, und 38 % bis 42 % mit den Faeces ausgeschieden sowie ca. 1 % als CO_2_ abgeatmet, wobei sich keine Unterschiede zwischen den Geschlechtern zeigten. Benzophenon-3 hatte im Blut eine Bioverfügbarkeit von < 1 %, was vermutlich durch einen First-Pass-Effekt in der Leber und eine Konjugation im Darm zu erklären ist (Mutlu et al. 2020).

Nach einer einmaligen Schlundsonden-Gabe von 3 bis 2570 mg ^14^C-Benzophenon-3/kg KG an männliche F344-Ratten wurden ≥ 63,9 % resorbiert und innerhalb von 72 Stunden 63,9 % bis 72,9 % mit dem Urin und 19,3 % bis 41,7 % mit den Faeces ausgeschieden. Im Körper verblieben nach dieser Zeit 0,1 % der applizierten Radioaktivität. Bei einmaliger Gabe war die Aufnahme nahezu vollständig und dosisunabhängig. Die Ausscheidung war nur bei der höchsten Dosis prozentual etwas geringer (El Dareer et al. 1986). Die Ausscheidung erfolgte als Glucuronsäure- oder Sulfat-Konjugat der Muttersubstanz oder der Metaboliten (Mutlu et al. 2020; Wnuk et al. 2022).

Die einmalige Schlundsonden-Gabe von 100 mg ^14^C-Benzophenon-3/kg KG an männliche und weibliche B6C3F1-Mäuse führte zu einer Ausscheidung von ≥ 24 % mit dem Urin, ≥ 24 % mit den Faeces und 5 % bis 15 % wurden als CO_2_ abgeatmet. Der Restgehalt an Radioaktivität im Gewebe lag bei Ratten und Mäusen nach 72 Stunden unterhalb von 1 % (Mutlu et al. 2020).

#### 3.1.3 Dermale Aufnahme

##### 3.1.3.1 Mensch

###### 3.1.3.1.1 In vitro

Für einige UV-filternde Substanzen wurden In-vitro- und In-vivo-Resorptionsdaten von **Sonnenlotion und -spray** verglichen. Für Benzophenon-3 stimmte das In-vitro-Modell gut mit den In-vivo-Daten überein. Wurde eine „infinite dose“ von 56,6 mg/cm^2^ mit der „finit dose“ von 2,0 mg/cm^2^ im HSE (Hitze-separierte Epidermis)-Hautmodell verglichen, zeigte sich bei der 28,3-fach höheren Dosis eine zehnfach höhere Aufnahme. Die Permeation in einem „all inclusive in vitro Permeation Test (IVPT)“ aus einer Sonnenlotion bei Verwendung einer einmaligen „finit dose“ von 2,0 mg/cm^2^ war > 2000 ng Benzophenon-3/cm^2^ in einem HSE-Hautmodell bei einer Applikationszeit von 24 Stunden. Die Resorption aus der Lotion war höher als aus einem Spray (Yang et al. 2022).

Wurde humane exzidierte Epidermis in Franz-Diffusionszellen untersucht, waren maximal 10 % der mit **Sonnenschutzmittel** aufgetragenen Benzophenon-3-Dosis, das entspricht 80 mg/m^2^, in acht Stunden in der Rezeptorflüssigkeit nachweisbar, etwa dieselbe Menge penetrierte in die Haut (Jiang et al. 1999). Daraus errechnet sich ein Flux von 2 µg/cm^2^ und Stunde in und durch die Haut.

###### 3.1.3.1.2 In vivo

Um die Aufnahme von Benzophenon-3 aus Kleidung durch die Haut zu untersuchen, hielten sich drei männliche Freiwillige in einem Krankenhaus auf und trugen T-Shirts, die mit Benzophenon-3 angereichert waren. Dafür wurden langärmelige T-Shirts 32 Tage lang in einer Kammer gegen eine steigende Konzentration von Benzophenon-3 exponiert, die Endkonzentration betrug 4,4 µg/m^3^. Die Freiwilligen trugen die exponierten T-Shirts sofort nach dem Herausholen aus der Expositionskammer für einen Zeitraum von drei Stunden. Danach trugen sie wieder ihre eigene Kleidung und der Urin wurde 48 Stunden lang gesammelt. Bereits im ersten Morgenurin, der vor dem Tragen des T-Shirts gesammelt wurde, betrug die Konzentration von Benzophenon-3 im Urin 30, 7 bzw. 17 ng/ml (25–75. Perzentil der US-Bevölkerung: 5,8–94 ng/ml). Die Aufnahme wurde anhand der Ausscheidung von Benzophenon-3, des Metaboliten Benzophenon-1 und sechs weiteren UV-Filtern mit dem Urin bestimmt. Für jeden Freiwilligen waren Alter, Gewicht, Größe, BMI und BSA (Körperoberfläche) bekannt und ähnlich (BMI 22,5; 21,9; 24,8; BSA 1,92; 1,89; 2,08). Die Mengen an Benzophenon-3 in den T-Shirts betrugen für Person 1, 2 und 3 (Mittelwert aus vier Proben) 2,3 µg/cm^2^, 1,4 µg/cm^2^ bzw. 1,4 µg/cm^2^. Die Personen 1, 2 und 3 schieden als Massensumme 12; 9,9 bzw. 82 µg Benzophenon-3 und Benzophenon-1 innerhalb von 48 Stunden (2. Morgenurin eingeschlossen) aus. Es wurden auch Serummessungen durchgeführt, jedoch werden nur über alle Personen gemittelte Werte berichtet: während und zwei Stunden nach der Exposition betrug der mittlere Serumwert 0,26 ± 0,13 ng/ml. Zum Messzeitpunkt 24 und 48 Stunden nach der Exposition betrugen die gemittelten Werte 0,13 ± 0,16 ng/ml (Morrison et al. 2017). Die Studie zeigt eine Aufnahme über die Kleidung, Berechnungen der Resorption bei direktem Hautkontakt sind nicht möglich.

In Untersuchungen mit **Sonnencremes** an 32 Freiwilligen, 15 jungen Männern und 17 Frauen in der Postmenopause, wurden 2 mg Sonnencreme/cm^2^ mit 10 % (G/G) Benzophenon-3 an vier Tagen am Morgen auf den gesamten Körper (bis auf Kopfhaut und Genitalbereich) appliziert und 20 Minuten einwirken lassen, bevor die Probanden sich wieder ankleiden durften. Frauen erhielten täglich im Mittel 35 ± 3 g Creme bei einer berechneten Körperoberfläche von 1,73 ± 0,14 m^2^, Männer 40 ± 3 g Creme bei einer berechneten Körperoberfläche von 1,99 ± 0,13 m^2^. Die Dosis entsprach etwa 50 mg Benzophenon-3/kg KG (KG ca. 70 kg). Innerhalb der ersten beiden Stunden nach der Applikation konnte Benzophenon-3 im Plasma bestimmt werden, war aber auch bei der letzten Messung nach 96 Stunden noch nachweisbar. Die höchsten Werte im Plasma (Medianwerte) wurden mit 187 ng/ml bei Frauen nach vier Stunden und mit 238 ng/ml bei Männern nach drei Stunden gemessen. Die höchsten Urinkonzentrationen (Medianwerte) betrugen 44 ng/ml im 24- bis 48-Stunden-Urin für Frauen (12 ng/ml im ersten 24-Stunden-Urin) und 81 ng/ml im 24-Stunden-Urin für Männer. Vor der Applikation lag der Gehalt an Benzophenon-3 in Plasma und Urin unterhalb der Nachweisgrenze von 3,9 ng/ml (Janjua et al. 2008). Bei 35 g Creme/Tag bei Frauen bzw. 40 g bei Männern errechnet sich eine Menge von 3,5 g bzw. 4 g Benzophenon-3, die täglich auf die Haut appliziert wurden. Innerhalb der ersten 24 Stunden schieden Frauen mit dem Urin 12 ng/ml und Männer 81 ng Benzophenon-3/ml aus, dies entspricht bei einer Ausscheidung von 1,5 l Urin/Tag 18 µg bzw. 121 µg. Der Stoff wird auch mit den Faeces ausgeschieden (bei Ratten: etwa 50 % der mit dem Urin ausgeschiedenen Menge (Mutlu et al. 2020; Wnuk et al. 2022)). Nimmt man dieses Verhältnis auch für den Menschen an, sind die ausgeschiedenen und damit resorbierten Mengen 27 bzw. 180 µg. Umgerechnet auf das Körpergewicht (70 kg) sind dies zwischen 0,35 µg/kg KG und 2,5 µg/kg KG. Dies erscheint im Vergleich zu den anderen Untersuchungen als sehr gering und ist daher wenig plausibel.

Elf Freiwillige, sieben Männer, vier Frauen im Alter von 22 bis 37 Jahren, trugen sich einmalig ca. 40 g **Sonnenschutzlotion** mit 4 % Benzophenon-3 auf die gesamte Körperhaut (bis auf Kopfhaut und Genitalbereich) auf (ca. 2 m^2^). Nach dem Einziehen der Lotion kleideten sie sich an und sammelten 48 Stunden lang ihren Urin. Jeder Proband hatte eine unterschiedliche Anzahl an Urinproben. Nach zwölf Stunden war einmal Duschen erlaubt. Die durchschnittlich mit dem Urin ausgeschiedene Menge betrug 11 mg, der Median 9,8 mg, was etwa 0,4 % der applizierten Menge entspricht. Vier der Freiwilligen schieden auch 48 Stunden nach der Applikation noch Benzophenon-3 mit dem Urin aus (Gustavsson Gonzalez et al. 2002). Bei angenommener zusätzlicher Ausscheidung von 50 % mit den Faeces (s. o.) wurden 15 mg resorbiert, bezogen auf 70 kg KG sind dies 0,21 mg/kg KG.

Die Untersuchung der Resorption von Benzophenon-3 aus **Sonnenschutzlotion** mit 4 % Benzophenon-3 mit und ohne anschließende Bestrahlung mit UVA- und UVB-Licht führte bei 25 Teilnehmern (16 Frauen, neun Männer, 22–42 Jahre alt) innerhalb von zehn Tagen zu einer Ausscheidung von 1,2 % bis 8,7 % (im Mittel 3,7 %) der applizierten Dosis mit dem Urin. Die Applikation erfolgte an fünf aufeinander folgenden Tagen je morgens und abends auf die gesamte Hautoberfläche, Kopfhaut und Genitalbereich ausgenommen. Erlaubt war eine Dusche pro Tag vor der zweiten Anwendung. Es wurde 2 mg Lotion/cm^2^ Haut gemäß der aus Größe und Gewicht ermittelten Körperoberfläche appliziert, dies waren zwischen 26 und 47 g Lotion bzw. 1,04 und 1,88 g Benzophenon-3 pro Person. Benzophenon-3 konnte noch drei bis fünf Tage nach der Gabe im Urin nachgewiesen werden. Die Bestrahlung mit UV-Licht veränderte die Menge der Benzophenon-3-Ausscheidung nicht. Die Häufigkeit der Anwendung von Sonnenlotion zeigte sich in den mit dem Urin ausgeschiedenen Mengen an Benzophenon-3: Bei Probanden mit häufiger Anwendung von Sonnencreme war die mit dem Urin ausgeschiedene Menge von Benzophenon-3 deutlich erhöht (Gonzalez et al. 2006). Aus der Studie ist ersichtlich, dass bei wiederholter Anwendung die resorbierte Menge deutlich ansteigt und die Halbwertszeit der Ausscheidung mit dem Urin ca. zwei Tage beträgt. 

In einer Untersuchung wurde je sechs Männern und sechs Frauen auf 75 % der Hautoberfläche **Sonnenschutzmittel** mit Benzophenon-3 aufgetragen und die Aufnahme über die Haut als maximale Plasmakonzentration gemessen. Die Probanden befanden sich dabei im Krankenhaus und waren nicht dem Tageslicht ausgesetzt. Die applizierte Menge betrug 2 mg Sonnenschutzmittel pro cm^2^ Haut und erfolgte am ersten Tag einmal, an den darauffolgenden drei Tagen viermal jeweils im Abstand von zwei Stunden entweder als Lotion oder als Aerosolspray, insgesamt 13 Applikationen. Die Lotion enthielt 4 % Benzophenon-3, das Aerosolspray 6 %. Die Plasmakonzentration wurde sieben Tage lang gemessen. Bei einer Person trat ein Hautausschlag auf. Die maximale Plasmakonzentration von Benzophenon-3 betrug mit Lotion 258,1 ng/ml und mit Aerosolspray 180,1 ng/ml und trat am vierten Tag auf. Die AUC betrug am vierten Tag 3443,7 (Lotion) bzw. 2757,3 ng × h/ml für das Aerosolspray. Die Halbwertzeiten lagen bei 78,5 und 79,2 Stunden für Lotion bzw. Aerosolspray (Matta et al. 2020). Es fehlt eine Angabe zur Ausscheidung, sodass keine Resorption berechnet werden kann.

Nach dermaler Exposition wird Benzophenon-3 beim Menschen auch in Muttermilch und Fruchtwasser nachgewiesen (Wnuk et al. 2022).

Die vorliegenden Daten zeigen eine gute dermale Resorption von Benzophenon-3 durch die menschliche Haut, die durch wiederholte Applikation deutlich zunimmt. Die aus den verschiedenen Untersuchungen berechneten resorbierten Mengen schwanken.

##### 3.1.3.2 Tier

###### 3.1.3.2.1 In vitro

In einer In-vitro-Untersuchung an **Schweine**haut wurde je eine Sonnenlotion- und Sonnencreme-Formulierung geprüft, die 2 % oder 6 % Benzophenon-3 als UV-Filter enthielt. Unter den Studienbedingungen penetrierten innerhalb von 24 Stunden ca. 3–4 % der Dosis die Haut (7,9 bzw. 6,7 µg/cm^2^ (2 % in Creme oder Lotion) oder 18,3 bzw. 19,3 µg/cm^2^ (6 % in Creme oder Lotion)) und waren in Rezeptorflüssigkeit plus Epidermis plus Dermis nachweisbar, also bioverfügbar; dies entspricht 4,0 bzw. 3,4 % oder 3,1 bzw. 3,3 % der applizierten Dosis (SCCP 2008). Aus den Daten mit 6 % in Creme oder Lotion errechnet sich ein Flux von 0,75 µg/cm^2^ und Stunde.

###### 3.1.3.2.2 In vivo

Wurden 51,6; 204 oder 800 µg ^14^C-Benzophenon-3 in Ethanol 72 Stunden lang dermal auf 1 cm^2^ Haut von männlichen Ratten appliziert, wurden innerhalb von 72 Stunden 32,4 %, 39,2 % bzw. 13,2 % der Radioaktivität mit dem Urin und 16,9 %, 22,2 % bzw. 9,15 % mit den Faeces ausgeschieden. Daraus errechnet sich eine Aufnahme von 49,3 %, 61,4 % bzw. 22,4 % der applizierten Dosis. Die prozentuale dermale Aufnahme nahm also bei der höchsten Dosis ab. Bei Applikation von 50 µg ^14^C-Benzophenon-3/cm^2^ in Lotion statt in Ethanol auf 1 cm^2^ Haut wurden 51,8 % resorbiert und 33,9 % mit dem Urin und 17,9 % mit den Faeces ausgeschieden, die Aufnahme war somit ähnlich (El Dareer et al. 1986). Bei einer Exposition gegen 50 µg/cm^2^ berechnet sich für eine 72-stündige Exposition unter der Annahme einer 50%igen Resorption ein Flux von 0,347 µg/cm^2^ und Stunde, bei Exposition gegen 800 µg/cm^2^ und 22 % Resorption in 72 Stunden ein Flux von 2,4 µg/cm^2^ und Stunde.

Bei Sprague-Dawley-Ratten und B6C3F1-Mäusen wurde die dermale Aufnahme im Bereich von 0,1 bis 15 mg ^14^C-Benzo­phenon-3/kg KG in verschiedenen Lösungsmitteln innerhalb von 72 Stunden nach einmaliger Gabe untersucht. Die Tiere saßen in einem Metabolismuskäfig und es wurden Urin, Faeces, Käfigluft und das Wasser, mit dem der Käfig gespült wurde, später die einzelnen Organe und der Kadaver untersucht. Bei den Tieren wurde auf 4 cm^2^ rasierter Hautfläche mit Methylacrylat-Zement ein Isolator aus Schaum oder Edelstahl angebracht, in den die Probe mit einer gasdichten Spritze durch Zwischenräume appliziert wurde. Diese Stellen wurden nach der Probengabe mit atmungsaktivem Leinen abgedeckt. Am Ende des Versuches wurden die Tiere getötet und die Hautfläche mit untersucht. Nach dermaler Gabe von 10 mg/kg KG an männliche Ratten lag die höchste Resorption bei 80 % bei Verwendung von leichtem Paraffinöl. Erfolgte die Gabe in Ethanol, Ethanol : Kokosnussöl (1:1) oder in Kokosnussöl, lag die maximale Resorption bei 64 % bis 73 %. Bei Verwendung einer Lotion aus Olivenöl und emulgierendem Wachs und Wasser (15:15:70 V:V:V) betrug sie 46 % bei männlichen Ratten (10 mg/kg KG) und 29 % bei weiblichen Ratten (15 mg/kg KG). Wurden 10 mg ^14^C-Benzophenon-3/kg KG männlichen oder weiblichen Mäusen aufgetragen, wurden 41–69 % aus Ethanol oder Aceton und 37–46 % aus einer Lotion heraus aufgenommen. Die Menge an nicht resorbierter Substanz bei männlichen Ratten betrug bei Gabe von 10 mg/kg (0,1 mg/kg) 1,5 % (6,7 %) bei Applikation in Paraffinöl, 51,7 % bei Applikation in Lotion, 10,7 % bei Applikation in Kokosnussöl, 4,41 % bei Applikation in Ethanol : Kokosnussöl und 16,2 % (1 mg/kg: 22,4 %) bei Applikation in Ethanol. Es zeigte sich keine dosisabhängige Resorption bei Ratten oder Mäusen und kein Unterschied zwischen den Spezies oder Geschlechtern (Mutlu et al. 2020). Damit errechnet sich für die Gabe von 10 mg/kg KG in Paraffinöl (80 % Resorption) ein Flux von ca. 7 µg/cm^2^ und Stunde und bei Applikation in Lotion (29 % Resorption) ein Flux von 2,5 µg/cm^2^ und Stunde. Dieselbe Berechnung ergibt bei Applikation von 0,1 mg/kg KG einen Flux von 70 ng/cm^2^ und Stunde (in Paraffinöl) bzw. 25 ng/cm^2^ und Stunde (in Lotion).

Weiblichen C57BL/6J-Mäusen wurden vom ersten bis zum sechsten Gestationstag täglich einmal 50 mg Benzophenon-3/kg KG in Olivenöl auf die rasierte Rückenhaut aufgetragen. Vier Stunden nach der dermalen Applikation am 6. Gestationstag betrug die Konzentration im Serum 22,4 ng/ml, angegeben als Mittelwert von drei Bestimmungen. Am achten Tag nach der letzten dermalen Applikation (14. Gestationstag) betrug die Konzentration im Serum noch 16,8 ng Benzophenon-3/ml (Einzelwert) und 22,63 ng/ml im Fruchtwasser (Mittelwert aus drei Messungen; Santamaria et al. 2020).

**Berechnung der Belastung bei dermaler Applikation**

Bei Ratten wurden dermale Aufnahmen von 20–60 % aus einer ethanolischen Lösung berichtet, der Flux betrug je nach applizierter Menge 0,347 µg/cm^2^ und Stunde (50 µg/cm^2^) oder 2,4 µg/cm^2^ und Stunde (800 µg/cm^2^) (El Dareer et al. 1986). In einer anderen Untersuchung an Ratten und Mäusen lag die dermale Resorption von Benzophenon-3 aus Paraffinöl bei Ratten bei 80 % und aus Lotion bei weiblichen Tieren bei 29 %. Daraus errechnen sich bei einer applizierten Dosis von 10 mg Benzophenon-3/kg KG Fluxe von ca. 7 µg/cm^2^ und Stunde aus Paraffinöl und 2,5 µg/cm^2^ und Stunde aus Lotion (Mutlu et al. 2020). Aus einer In-vitro-Untersuchung an Schweinehaut ergibt sich ein Flux von 0,75 µg/cm^2^ und Stunde (siehe oben; SCCP 2008). 

Für eine dermale Belastung am Arbeitsplatz errechnen sich aus dem niedrigsten Flux mit einer ethanolischen Lösung an Ratten und dem höchsten Flux aus Paraffinöl bei Ratten bei einer einstündigen Exposition von 2500 cm^2^ Haut (Hände, Unterarme und Gesicht) Aufnahmemengen von 867 bzw. 17 500 µg Benzophenon-3. Zieht man den Flux der In-vitro-Studie an Humanhaut mit 2 µg/cm^2^ und Stunde heran (s. o.), ergibt sich eine aufgenommene Menge von 5000 µg Benzophenon-3.

Aus den dargestellten Daten beträgt die maximal resorbierte Dosis 17,5 mg, bezogen auf 70 kg Körpergewicht sind dies 0,25 mg/kg KG. Dieser Wert ist sehr konservativ, weil der Flux durch die Rattenhaut höher ist als der Flux durch Humanhaut. Aus der In-vitro-Studie mit Humanhaut ergibt sich eine Dosis von 5 mg, bezogen auf 70 kg KG sind dies 0,07 mg/kg KG.

Anhand des Modells von Fiserova-Bergerova (Fiserova-Bergerova et al. 1990) errechnet sich ein Flux von 51 µg/cm^2^ und Stunde und eine aufgenommene Menge von 127,5 mg pro 2500 cm^2^.

Nach IH SkinPerm (Tibaldi et al. 2014) beträgt der Flux 1,64 µg/cm^2^ und Stunde und die aufgenommene Menge 4,1 mg pro 2500 cm^2^.

##### 3.1.3.3 Fazit

Es liegen keine Daten zur inhalativen Aufnahme vor. Die orale Aufnahme erfolgt beim Tier im Dosisbereich von 3 bis 2500 mg/kg KG bei einmaliger Gabe nahezu vollständig und dosisunabhängig. Bei der oralen Aufnahme findet ein starker First-Pass-Effekt statt, sodass die Bioverfügbarkeit im Blut ca. 1 % beträgt. Benzophenon-3 verteilt sich mit dem Blut in alle Organe und wird daraus schnell wieder eliminiert. Auch bei dermaler Gabe zeigt sich eine ähnliche Resorption und Verteilung in den Organen wie bei oraler Gabe. Die Resorption ist jedoch vehikelabhängig und z. B. in leichtem Paraffinöl besonders gut. Auch die Ausscheidung ist dosisunabhängig und es verbleiben zwei Tage nach der Applikation von radioaktiv markiertem Benzophenon-3 weniger als 1 % der applizierten Radioaktivität (nach drei Tagen < 0,1 %) im Körper, der Rest wird metabolisiert und mit Urin (zwei Drittel) und Faeces ausgeschieden, ein sehr geringer Anteil von ca. 1 % wird als CO_2_ abgeatmet (Mutlu et al. 2020).

Auch beim Menschen wurde eine dermale Resorption nachgewiesen. Benzophenon-3 kann möglicherweise die Blut-Hirn-Schranke passieren und findet sich in Fruchtwasser und Muttermilch (Wnuk et al. 2022). Da bei dermaler Gabe fast ausschließlich Sonnenschutzformulierungen untersucht wurden, ist eine quantitative Aussage zur dermalen Resorption von Benzophenon-3 beim Menschen aus anderer Matrix nicht möglich. In einer In-vitro-Studie mit Humanhaut betrug der Flux 2 µg/cm^2^ und Stunde, bei 20%iger Resorption (durch und in die Haut). Bei Ratten wurden dermale Aufnahmen von 20–60 % aus einer ethanolischen Lösung berichtet, der Flux betrug je nach applizierter Menge 0,347 µg/cm^2^ und Stunde (50 µg/cm^2^) oder 2,4 µg/cm^2^ und Stunde (800 µg/cm^2^) (El Dareer et al. 1986). In einer anderen Untersuchung an Ratten und Mäusen lag die dermale Resorption von Benzophenon-3 aus leichtem Paraffinöl bei männlichen Ratten bei 80 % und aus Lotion bei weiblichen Ratten bei 29 %. Daraus errechnen sich bei einer applizierten Dosis von 10 mg Benzophenon-3/kg KG ein Flux von ca. 7 µg/cm^2^ und Stunde aus Paraffinöl und 2,5 µg/cm^2^ und Stunde aus Lotion (Mutlu et al. 2020). Aus einer In-vitro-Untersuchung an Schweinehaut (SCCP 2008) ergibt sich ein Flux von 0,75 µg Benzophenon-3/cm^2^ und Stunde für die Applikation in Sonnenschutzmittel.

### 3.2 Metabolismus

#### 3.2.1 Mensch

Es liegen nur wenige Daten am Menschen vor. In einer Untersuchung wurden bei Auftragung von Benzophenon-3 auf die Haut (k. w. A.) im Serum und dem Urin Benzophenon-3 und die Metaboliten 2,4-Dihydroxybenzophenon (DHB, Benzo­phenon-1) und 2,2′-Dihydroxy-4-methoxybenzophenon (2,2′-DHMB, Benzophenon-8) nachgewiesen (NTP 2020). Bei In-vitro-Inkubation von Benzophenon-3 mit humanen Lebermikrosomen wurden die vier Hauptmetaboliten 2,3,4-Trihydroxybenzophenon, 2,4,5-Trihydroxybenzophenon, 2,4-Dihydroxybenzophenon und 2,5-Dihydroxy-4-methoxybenzophenon nachgewiesen (Watanabe et al. 2015). Die Metaboliten 2,2′-Dihydroxy-4-methoxybenzophenon und 2,4,5-Trihydroxybenzophenon wurden bei Untersuchungen mit Ratte und Maus nicht berichtet.

#### 3.2.2 Tier

**Abb. 1:**
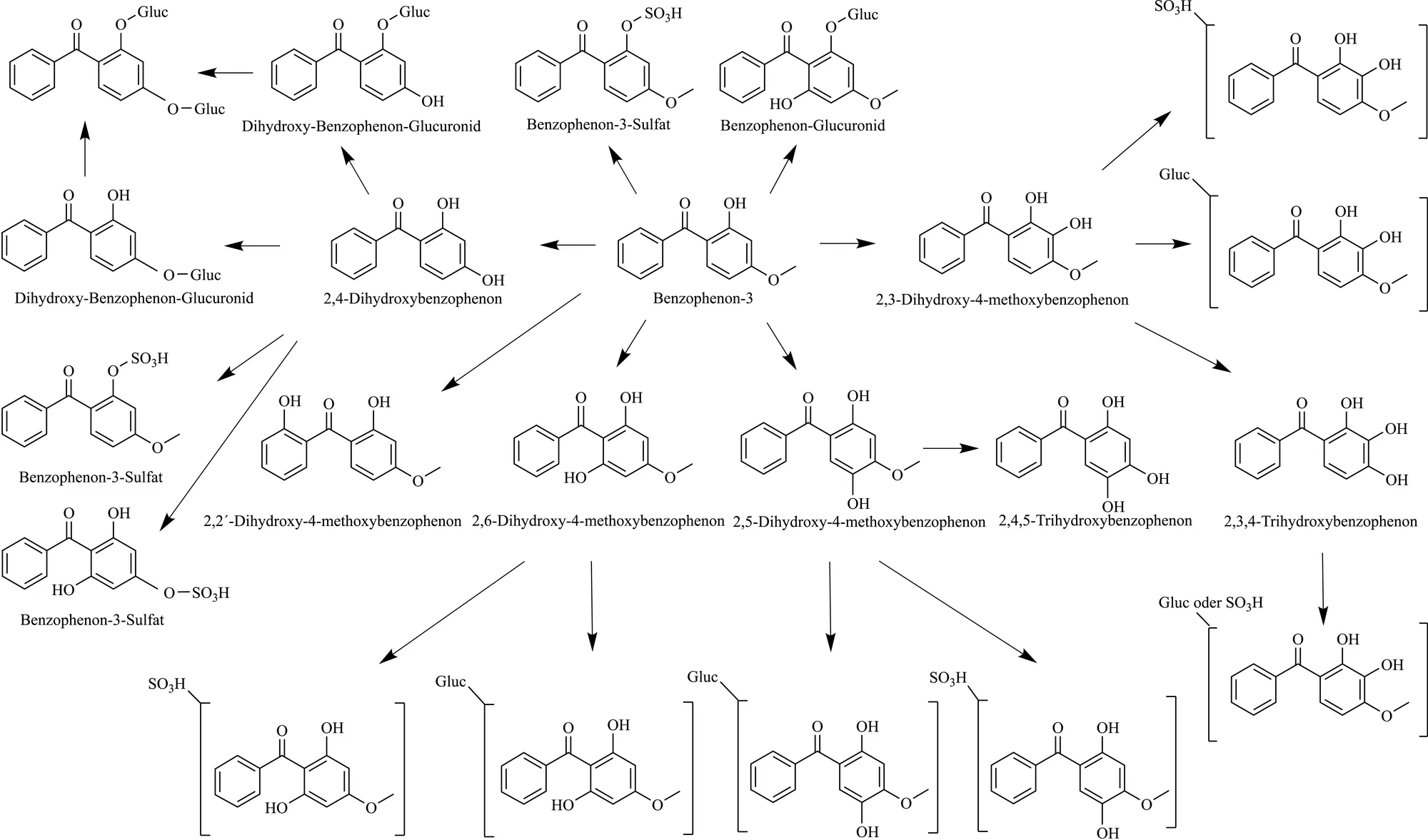
Metabolismus von Benzophenon-3 bei Mensch, Ratte, Maus. 2,2′-Dihydroxy-4-methoxybenzophenon (Benzophenon-8; nur Mensch, Serum, Urin); 2,4,5-Trihydroxybenzophenon (nur mit humanen Lebermikrosomen). Gluc: Glucoronid

Untersucht wurde der Metabolismus unter anderem nach einmaliger Schlundsondengabe von 10, 100 oder 500 mg/kg KG, einmaliger intravenöser Gabe von 10 mg/kg KG oder einmaliger dermaler Gabe von 0,1; 1; 10 oder 15 mg/kg KG von ^14^C-Benzophenon-3 an Ratten und Mäuse. In Gallenflüssigkeit und Urin wurden folgende Metaboliten nachgewiesen (siehe Abbildung 1): unverändertes Benzophenon-3 (HMB), 2,4-Dihydroxybenzophenon (DHB), 2,3-Dihydroxy-4-methoxybenzophenon (2,3-DHMB), 2,5-Dihydroxy-4-methoxybenzophenon (2,5-DHMB), 2,3,4-Trihydroxybenzophenon (THB), 2,5-Dihydroxy-4-methoxybenzophenon (D2H4MBP) und die korrespondierenden Glucuronid- und Sulfat-Konjugate. Der Hauptmetabolit von Benzophenon-3 bei Ratten ist DHB (Mutlu et al. 2020; Wnuk et al. 2022).

Benzophenon-3 und 2,4-Dihydroxybenzophenon wurden im Serum von trächtigen Ratten quantifiziert. Bei in utero und anschließend gegen 0, 3000, 10 000 oder 30 000 mg/kg Futter exponierten Ratten wurde die Plasmakonzentration von nicht konjugiertem, und der Gesamtgehalt von konjugiertem und nicht konjugiertem Benzophenon-3, 2,4-Dihydroxybenzophenon, 2,3,4-Trihydroxybenzophenon und 2,5-Dihydroxy-4-methoxybenzophenon bestimmt. Im Plasma trat kein freies 2,5-Dihydroxy-4-methoxybenzophenon oder 2,3,4-Trihydroxybenzophenon auf. Die Gesamtmenge an freiem und gebundenem Benzophenon-3 und 2,4-Dihydroxybenzophenon im Plasma war 100- bis 300-fach höher als der Anteil an den entsprechenden freien Verbindungen. Die Gesamtmenge an freien und gebundenen Verbindungen war für Benzophenon-3 ähnlich der von 2,4-Dihydroxybenzophenon, beide Mengen größer als die von 2,5-Dihydroxy-4-methoxybenzophenon und diese deutlich größer als die von 2,3,4-Trihydroxybenzophenon. Die Menge an freien oder konjugierten Verbindungen im Plasma war am 28. oder 56. Lebenstag nicht geschlechtsabhängig verschieden (NTP 2020).

Die vorliegenden Daten zeigen, dass Benzophenon-3 bei Nagern umfassend metabolisiert und nur ein geringer Teil unverändert mit dem Urin ausgeschieden wird. In den vorliegenden Untersuchungen verlaufen Metabolisierung und die Ausscheidung dosisunabhängig. Beschriebene Reaktionen des Metabolismus sind eine Demethylierung, eine Oxidation an der Ringstruktur, eine Glucuronidierung von Benzophenon-3 sowie seiner Metaboliten sowie Sulfatierung derselben. Das Metabolismusschema ist in Abbildung 1 dargestellt.

## 4 Erfahrungen beim Menschen

### 4.1 Einmalige Exposition

Hierzu liegen keine Daten vor.

### 4.2 Wiederholte Exposition

Es liegen nur Daten zur dermalen Resorption vor, die im Abschnitt 3.1.3.1 beschrieben sind.

### 4.3 Wirkung auf Haut und Schleimhäute

Hierzu liegen keine Daten vor.

#### Phototoxizität

In vivo wurden Photopatch-Tests mit 28 hautgesunden Teilnehmern durchgeführt. Dazu wurden unterschiedliche Zubereitungen aus einem phosphatbasierten selbstemulgierenden Wachs, verschiedenen UV-Filterkombinationen sowie Retinylpalmitat hergestellt. Die Benzophenon-3-haltige Testzubereitung führte bei keinem der Teilnehmer nach 24, 48 oder 72 Stunden zu Erythemen, Ödemen, Papeln oder Bläschen und wurde als nicht phototoxisch bewertet (Benevenuto et al. 2015).

### 4.4 Allergene Wirkung

#### 4.4.1 Hautsensibilisierende und photosensibilisierende Wirkung

An der hautsensibilisierenden Wirkung von Benzophenon-3 besteht kein Zweifel.

Im Zusammenhang mit Sonnenschutzmitteln treten Sensibilisierungen bei Gesunden jedoch nur selten auf. In einer Auswertung von in verschiedenen Zentren durchgeführten Human Repeated Insult Patch Tests (HRIPT) und Photosensibilisierungstests mit Sonnenschutzmitteln trat eine Sensibilisierung nur bei 0,26 % der gesunden Personen auf. Es wurden insgesamt 19 570 Personen mit 89 verschiedenen Sonnenschutzmitteln getestet, welche zwischen 1 % und 6 % Benzophenon-3 enthielten. Im HRIPT erfolgten neun okklusive Induktionsbehandlungen im Abstand von zwei Tagen (k. A. über applizierte Dosis). Zehn bis 14 Tage nach der letzten Induktionsbehandlung wurde die erste Provokation zwei Tage lang an der gleichen Hautstelle durchgeführt. Nach Bewertung der Reaktion erfolgte eine zweite Provokation, erneut für zwei Tage an einer anderen Hautstelle. Beide Hautstellen wurden nach vier Tagen evaluiert. Insgesamt wurden 0,26 % positive Reaktionen beobachtet. Die Anzahl der im HRIPT getesteten Personen ist unklar. Zur Untersuchung der photosensibilisierenden Wirkung der verschiedenen Sonnenschutzmittel wurden in der Induktionsphase jeweils 20 µl/cm^2^ okklusiv appliziert. Nach einem Tag wurde das Pflaster entfernt, die Hautstelle abgewischt und erneut 2 µl/cm^2^ appliziert. Nach 5–15 Minuten erfolgte die Belichtung mit der doppelten individuell bestimmten minimalen effektiven Dosis (MED). Die Prozedur wurde zweimal wöchentlich drei Wochen lang wiederholt. Neun bis 16 Tage nach der letzten Induktionsbehandlung erfolgte eine Provokation mit zwei Applikationen identisch zur Induktionsphase. Die Belichtung mit einer höheren Dosis erfolgte an einer der beiden Hautstellen mit 10 J/cm^2^ plus der Hälfte der ermittelten MED. Des Weiteren wurde eine nicht behandelte Hautstelle mit der gleichen Dosis, die für die Provokation eingesetzt wurde, belichtet. Die Auswertung erfolgte ein, zwei und drei Tage nach der Belichtung. Es wurden bei 0,27 % positive photokontaktallergische Reaktionen beobachtet (Agin et al. 2008).

Es gibt eine Vielzahl an Studien und Fallberichten über Epikutantestungen und Photoepikutantestungen mit Benzophenon-3, wobei in diesen Untersuchungen eine starke Selektion von Personen mit Hautproblemen vorliegt. Die Testung erfolgte weltweit, sehr häufig bei konkretem Verdacht auf eine Photokontaktallergie im Zusammenhang mit Sonnenschutzmitteln oder Kosmetika wie Lippenbalsam. Während in älteren Studien (1992 bis ca. 2000) die Testung mit einer 2%igen Zubereitung in Vaseline erfolgte, wurde in neueren Studien (etwa ab 2000) die 10%ige Testzubereitung verwendet.

Für Photoepikutantests werden die gleichen Testzubereitungen verwendet wie für den Epikutantest. Die Durchführung eines Photoepikutantests erfolgt nach europäischen (Bruynzeel et al. 2004; Johansen et al. 2015) bzw. nordamerikanischen Richtlinien (DeLeo et al. 2024) durch Applikation der Testzubereitung an zwei Hautstellen. Nach einem oder zwei Tagen erfolgt die Belichtung einer der beiden Hautstellen, idealerweise mit einer individuell bestimmten Dosis, i. d. R. jedoch 5 J/cm^2^ nach europäischer Richtlinie (Bruynzeel et al. 2004; Johansen et al. 2015) oder 10 J/cm^2^ nach nordamerikanischer Richtlinie (DeLeo et al. 2024). Die Ablesung wird zwei Tage nach Belichtung (entsprechend 3. oder 4. Tag nach der Applikation) und ggf. zusätzlich später durchgeführt. Die Spätablesung erscheint hierbei sehr wichtig, da photoallergische Reaktionen teilweise erst am 6. Tag nach der Applikation (vier Tage nach Belichtung) auftreten können. In einer Studie wurden beispielsweise von insgesamt elf positiven Reaktionen auf Benzophenon-3 (10 %) fünf am 4. Tag und sechs erst am 6. Tag nach der Applikation beobachtet (Valbuena Mesa und Hoyos Jiménez 2016).

Die Auswertung erfolgt laut Richtlinien wie folgt: Ist nur eine Reaktion an der unbelichteten Hautstelle oder sowohl an der unbelichteten als auch an der belichteten Hautstelle zu sehen, wird dies als Kontaktallergie gewertet. Ist eine Reaktion an der unbelichteten Hautstelle und eine stärkere Reaktion an der belichteten Hautstelle zu beobachten, handelt es sich um eine Kontaktallergie mit Lichtverstärkung. Zeigt sich nur eine Reaktion an der belichteten Hautstelle, handelt es sich um eine Photokontaktallergie.

Ergebnisse verschiedener Studien (n > 100), in denen Epikutantests oder Photoepikutantests mit Benzophenon-3 durchgeführt wurden, sind in Tabelle 1 exemplarisch zusammengestellt. Von diesen insgesamt 34 Studien wurden 26 zur Bewertung berücksichtigt. Acht Studien (Crouch et al. 2002; Darvay et al. 2001; Gonçalo et al. 1995; Greenspoon et al. 2013; Pigatto et al. 1996; Scalf et al. 2009; Shao et al. 2021; Szczurko et al. 1994) wurden nicht berücksichtigt, da sie bezüglich Testmaterial oder Zeitpunkt der Ablesung unvollständig dokumentiert sind oder die Durchführung des Photoepikutantests erfolgte nicht nach bzw. analog zu den Richtlinien (z. B. erfolgte teilweise die Ablesung nur einen Tag nach Belichtung oder es wurde eine zu geringe Dosis zur Belichtung verwendet).

Die Testungen in den 26 Studien erfolgten im Zeitraum zwischen 1981 und 2020. Insgesamt wurden 74 532 Personen getestet, zwei Drittel davon waren Frauen. Die Personen waren zwischen 11 und 80 Jahre alt, mit einem durchschnittlichen Alter von 41 bis 56 Jahren. Es zeichnet sich kein Unterschied in den Reaktionsquoten in älteren Studien mit 2%iger und neueren Studien mit 10%iger Testzubereitung ab. Ein Trend der Prävalenz im Verlauf des Testzeitraums ist nicht erkennbar, allerdings sind tendenziell etwas höhere Reaktionsquoten bei Belichtung mit 10 J/cm^2^ im Vergleich zu 5 J/cm^2^ zu beobachten.

In Studien, in denen Personen mit Verdacht auf eine (Photo-)Kontaktallergie im Zusammenhang mit Sonnenschutzmitteln, Kosmetika oder Arzneimitteln getestet wurden, lagen die Reaktionsquoten im Epikutantest zwischen 0 und 3,7 %. In Studien mit Testung bei Verdacht auf eine (Photo-)Kontaktallergie (ohne Angabe der vermuteten Expositionsquelle) oder andere lichtbedingte Hauterkrankungen wurden Reaktionsquoten zwischen 0 und 5,2 % beobachtet. 

Mit Benzophenon-3 kann es nach UV-Lichtexposition, wie auch bei einigen weiteren Kontaktallergenen, zu einer verstärkten Reaktion kommen. In einigen Publikationen wurde zusätzlich die Anzahl lichtverstärkter Reaktionen angegeben (0,1–2,0 %). Eine alleinige Photokontaktallergie wurde bei 2,3–11,8 % der Personen mit Verdacht auf Kontaktallergie im Zusammenhang mit Sonnenschutzmitteln, Kosmetika oder Arzneimitteln und bei 1,0–7,7 % der Personen mit Verdacht auf eine Photokontaktallergie (ohne Angabe der vermuteten Expositionsquelle) oder eine andere lichtbedingte Hauterkrankung festgestellt. 

Insgesamt lässt sich aus den Ergebnissen der in Tabelle 1 aufgeführten Studien ableiten, dass Photokontaktallergien gegen Benzophenon-3 etwa zwei- bis dreimal so häufig auftreten wie Kontaktallergien. Einige Studien spiegeln dieses Ergebnis wider (Bryden et al. 2006; Chaiyabutr et al. 2021; DeLeo et al. 2024), ebenso eine Auswertung in einem Übersichtsartikel, in dem insgesamt 118 kontaktallergische und 360 photokontaktallergische Reaktionen auf Benzophenon-3 erfasst wurden (Ekstein und Hylwa 2023). Im Gegensatz hierzu treten bei Benzophenon-4 häufiger kontaktallergische Reaktionen als photokontaktallergische Reaktionen auf.

Teilweise wurden in den Studien Reaktionsstärken angegeben. Im Epikutantest wurden vornehmlich +- und ++-Reaktionen beobachtet. In einer Studie (n = 19 618) wurden beispielsweise 51 × +-, 22 × ++- und 15 × +++-Reaktionen beobachtet (Warshaw et al. 2023). Die Reaktionsstärken im Photoepikutantest wurden nur in einer Studie (n = 1031) angegeben, hier war ein ähnliches Reaktionsprofil zu beobachten (14 × +, 18 × ++, 5 × +++; EMCPPTS Taskforce 2012).

**Tab. 1:** Studien (n > 100) über Hautreaktionen auf Benzophenon-3 in Epikutantests und Photoepikutantests bei Personen mit Verdacht auf Kontaktallergie und/oder Photokontaktallergie

**Anzahl getesteter Personen**	**Konzentration, in Vaseline**	**Ergebnis**			**Bemerkungen**	**Referenz**
		**Kontaktallergie**	**Kontaktallergie mit Lichtverstärkung**	**nur Photokontaktallergie**		
**Vermutete Expositionsquelle: Sonnenschutzmittel, andere Kosmetika und/oder Arzneimittel**						
270	2 %	7 (2,6 %)	5 (1,9 %)	32 (11,8 %)	Bestrahlung mit 8–10 J/cm^2^ an D2	Pons-Guiraud und Jeanmougin 1993
214	2 %	8 (3,7 %)	n. d.	n. d.		Bilsland und Ferguson 1993
108	2 %	0	1 (0,9 %)	3 (2,8 %)	Bestrahlung mit 10 J/cm^2^ an D2	Trevisi et al. 1994
365	10 %, teilweise 2 %	3 (0,8 %)	k. A.	9 (2,5 %)	Bestrahlung mit 5 oder 10 J/cm^2^, Bestrahlung vermutlich an D1	Schauder und Ippen 1997
475	k. A., vermutlich 2 %	14 (2,9 %)	n. d.	n. d.		Goossens et al. 1999
370	2 %, teilweise 10 %	2 (0,5 %)	4 (1,1 %)	21 (5,7 %)	Bestrahlung mit 13 J/cm^2^ oder individueller Dosis an D1	Journe et al. 1999
1155	10 %	9 (0,8 %)	1 (0,1 %)	27 (2,3 %)	Bestrahlung mit 5 J/cm^2^ (teilweise 2,5 oder 1 J/cm^2^) an D1 oder D2	Bryden et al. 2006
553	10 %	3 (0,5 %)	n. d.	n. d.		Hughes und Stone 2007
1031	10 %	6 (0,6 %)	k. A.	37 (3,5 %)	Bestrahlung mit 5 J/cm^2^ (teilweise < 5 J/cm^2^) an D1 oder D2	EMCPPTS Taskforce 2012
23 908	10 oder 3 %	82 (0,3 %)	n. d.	n. d.		Warshaw et al. 2013
1527	10 %	8 (0,5 %)	n. d.	n. d.		Beleznay et al. 2014
4224	10 %	7 (0,2 %)	n. d.	n. d.		Rolls et al. 2021
19 618	10 %	104 (0,5 %)	n. d.	n. d.		Warshaw et al. 2023
**Verdacht auf Photoallergie (ohne Angabe der vermuteten Expositionsquelle) oder andere photosensitive Hauterkrankung**						
355	2 %	1 (0,3 %)	k. A.	15 (4,2 %)	Bestrahlung mit 5 oder 10 J/cm^2^ an D1, zusätzlich wurde eine urtikarielle Reaktion beobachtet.	Berne und Ros 1998
167	k. A., vermutlich 2 %	0	k. A.	5 (3 %)	Bestrahlung mit 5 J/cm^2^ an D1	Bell und Rhodes 2000
99	10 %	0	2 (2,0 %)	1 (1,0 %)	Bestrahlung mit 50–70 % der individuellen Dosis, durchschnittlich 17 J/cm^2^	Bakkum und Heule 2002
5800	3 %	35 (0,6 %)	n. d.	n. d.		Marks et al. 2003
2067	10 %	k. A.	1 (0,1 %)	54 (2,6 %)	Bestrahlung mit 10 J/cm^2^ oder 5 J/cm² an D1	Leonard et al. 2005
207	10 %	k. A.	k. A.	5 (2,4 %)	Bestrahlung mit 5 J/cm^2^ an D2	Katsarou et al. 2008
4859	10 %	31 (0,6 %)	n. d.	n. d.		DeKoven et al. 2017
116	k. A., vermutlich 10 %	2 (1,7 %)	k. A.	2 (1,7 %)	Bestrahlung mit 5 J/cm^2^ an D2	Subiabre-Ferrer et al. 2019
170	10 %	3 (1,8 %)	k. A.	8 (4,7 %)	Bestrahlung mit 10 J/cm^2^ an D2	Chaiyabutr et al. 2021
5957	k. A., vermutlich 10 %	14 (0,2 %)	n. d.	n. d.		Geier und Schubert 2021
363	10 %	19 (5,2 %)	5 (1,4 %)	28 (7,7 %)	Bestrahlung mit 10 J/cm^2^ (teilweise geringere Dosis) an D2 oder D3	DeLeo et al. 2022
454	10 %	17 (3,7 %)	1 (0,2 %)	28 (6,2 %)	Bestrahlung mit 10 J/cm^2^ (teilweise 5 J/cm^2^) an D2 oder D3	DeLeo et al. 2024
**Verdacht auf Photoallergie (ohne Angabe der vermuteten Expositionsquelle), andere lichtbedingte Hauterkrankungen bereits ausgeschlossen**						
100	10 %	4 (4 %)	k. A.	11 (11 %)	Bestrahlung mit 5 J/cm^2^ an D2	Valbuena Mesa und Hoyos Jiménez 2016
**Testung nicht nach Richtlinie bzw. unvollständig dokumentiert**						
283	2 %	1 (0,35 %)	k. A.	21 (7,4 %)	Bestrahlung mit 35 mW/cm^2^ (k. A. über Dauer, daher keine Berechnung von J/cm^2^ möglich) UVA und 1,5 mW/cm^2^ UVB an D1 nach Applikation, Ablesung 1 Tag nach Bestrahlung	Szczurko et al. 1994
1233	2 %	2 (0,2 %)	k. A.	5 (0,4 %)	Bestrahlung mit 5 J/cm^2^, k. A. zum Zeitpunkt der Ablesung	Gonçalo et al. 1995
unklar	vermutlich 2 %	k. A.	k. A.	10 (1,0 %)	Bestrahlung mit 5 J/cm^2^ an D2	Pigatto et al. 1996
unklar	2 oder 10 %	8 (0,3 %)	k. A.	14 (0,5 %)	Bestrahlung mit 5 J/cm^2^ (teilweise 2,5 oder 1 J/cm^2^) an D2 nach Applikation	Darvay et al. 2001
149	2 %	1 (0,7 %)	0	3 (2,0 %)	Bestrahlung mit 5 J/cm^2^ an D2 nach Applikation, Ablesung 1 Tag nach Bestrahlung	Crouch et al. 2002
178	3 %	3 (1,7 %)		5 (2,8 %)	überwiegend mit 1 J/cm^2^ bestrahlt, teilweise mit individueller Dosis, teilweise mit 5 oder 10 J/cm^2^	Scalf et al. 2009
160	k. A.	17 (10,6 %)	6 (3,8 %)	6 (3,8 %)	Bestrahlung mit 5 J/cm^2^ an D1, Ablesung 1 Tag nach Bestrahlung	Greenspoon et al. 2013
2372	1 %	10 (0,4 %)	k. A.	10 (0,4 %)	mit „Benzophenon (UV-Absorber)“, Angabe ungenau, vermutlich Benzophenon-3 gemeint, Bestrahlung mit 10 J/cm^2^ (in Ausnahmefällen geringere Dosis) an D1 nach Applikation	Shao et al. 2021

D: Tag nach Applikation; k. A.: keine Angabe; n. d.: nicht durchgeführt

Insgesamt werden die Ergebnisse der größeren Studien zu Kontaktallergien durch weitere kleinere Studien (n < 100, z. B. Ang et al. 1998; Camarasa und Serra-Baldrich 1986; Cook und Freeman 2001; DeLeo et al. 1992; Lenique et al. 1992; Lim et al. 2023; Matthieu et al. 2004; Spiewak 2013) sowie Fallberichte (z. B. Alanko et al. 2001; Camarasa und Serra-Baldrich 1986; Hanson und Warshaw 2015; Landers et al. 2003; Sasseville et al. 2011; Schram et al. 2007) unterstützt, welche alle im Zusammenhang mit Sonnenschutzmitteln oder Kosmetikprodukten stehen. Auch die Ergebnisse der größeren Studien mit Photoepikutantestung werden durch weitere kleinere Studien (n < 100, z. B. Ang et al. 1998; Avenel-Audran et al. 2010; Cardoso et al. 2009; Chuah et al. 2013; Cook und Freeman 2001; DeLeo et al. 1992; Kim et al. 2021; Lenique et al. 1992; Leow et al. 1994; Lim et al. 2023; Matthieu et al. 2004; Rodríguez et al. 2006; Russo et al. 2018; Spiewak 2013; Thune 1984; Victor et al. 2010), einer Studie an Kindern (Haylett et al. 2014) sowie Fallberichten (z. B. Aguirre et al. 1992; Collins und Ferguson 1994; Green et al. 1991; Hanson und Warshaw 2015; Hölzle und Plewig 1982; Kiec-Swierczynska et al. 2005; Knobler et al. 1989; Langan und Collins 2006; Nedorost 2003; Peluso et al. 1991; Ricci et al. 1998; Schmidt et al. 1998; Silva et al. 1995; Thompson et al. 1977; Veysey und Orton 2006; Wahie et al. 2007; Zhang et al. 1998) unterstützt, welche ebenfalls alle im Zusammenhang mit Sonnenschutzmitteln oder Kosmetikprodukten stehen. Lediglich in einer Publikation wird über eine positive Photoepikutantestreaktion auf Benzophenon-3 im Zusammenhang mit einem Sport-T-Shirt, welches Benzophenon-3 als Lichtschutz enthielt, berichtet (García Castro et al. 2021).

##### Beruflicher Zusammenhang

Es liegt nur eine Studie mit einer Unterteilung in eine berufliche und nicht-berufliche Kontaktdermatitis vor, aus der keine Unterschiede hervorgehen. Es wurden 585 Personen mit beruflich bedingter Kontaktdermatitis und 4787 Personen mit einer nicht-beruflichen Kontaktdermatitis mit Benzophenon-3 (10 %) getestet. In beiden Kollektiven lag die Prävalenz bei 0,2 % (Geier und Schubert 2021). In einer anderen Untersuchung wird über eine berufliche Relevanz bei einer von 104 Personen mit positivem Epikutantest auf Benzophenon-3 (10 %) berichtet. Die Person gehörte dem Beschäftigungsfeld Kosmetikinstitut/Friseursalon an. Als mögliche Expositionsquelle wurden Haarprodukte und Nagellack angegeben (Warshaw et al. 2023).

In einer weiteren Studie zu beruflich bedingter Kontaktdermatitis bei Farmern in Neuseeland reagierte eine von 46 untersuchten Personen positiv auf Benzophenon-3 (2 %), die Reaktion wurde von den Autoren als relevant bewertet (Rademaker 1998). Es fehlen jedoch Angaben über die (vermutete) Ursache sowie Angaben zu weiteren positiven Reaktionen auf andere getestete Allergene. Fallberichte über eine (Photo-)Kontaktallergie gegen Benzophenon-3 mit beruflichem Zusammenhang liegen nicht vor. Insgesamt lässt sich aufgrund der sehr weiten Verbreitung von Benzophenon-3 und Kontaktmöglichkeiten, z. B. durch Verwendung eines Benzophenon-3-haltigen Sonnenschutzmittels, eine rein berufliche Exposition nicht abgrenzen.

##### Kreuzelizitationen

**Abb. 2:**
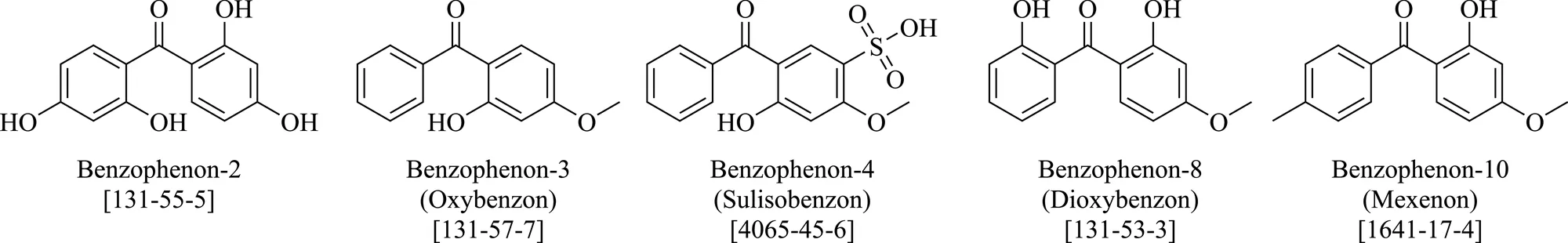
Strukturformeln und CAS-Nummern von Benzophenon-2, Benzophenon-3, Benzophenon-4, Benzophenon-8 und Benzophenon-10

Da Benzophenon-3 und Benzophenon-4 (Abbildung 2) in Kosmetikprodukten weit verbreitet sind, ist eine Exposition gegenüber beiden Stoffen wahrscheinlich. Dennoch reagieren die meisten Personen, die mit beiden Benzophenonen getestet werden, nur auf eines. In einer Untersuchung wurde beispielsweise bei 104 Personen mit Kontaktallergie gegen Benzophenon-3 nur bei 14 Personen (13,5 %) auch eine Reaktion auf Benzophenon-4 festgestellt. Umgekehrt zeigten von 322 Personen mit Kontaktallergie gegen Benzophenon-4 nur insgesamt 14 Personen (4,3 %) auch eine Reaktion gegen Benzophenon-3. Bei acht der 14 Patienten war eine Koexposition gegen beide Benzophenone in Sonnencreme, Körper- und Haarpflegeprodukten bekannt (Warshaw et al. 2023).

Weitere Studien und Einzelfallberichte unterstützen diese Ergebnisse und zeigen ebenfalls, dass gleichzeitige (photoallergische) Reaktionen auf Benzophenon-3 und Benzophenon-4 auftreten, insgesamt aber selten sind (untersucht z. B. von Hanson und Warshaw 2015; Le Coz et al. 1998; Leroy et al. 1997; Nedorost 2003; Spiewak 2013).

Gleichzeitige positive Reaktionen auf weitere Benzophenone (Benzophenon-2, Benzophenon-8 oder Benzophenon-10, Abbildung 2), die seltener als UV-Filter verwendet werden, sind trotz ähnlicher Strukturen sehr selten (Ekstein und Hylwa 2023; Hanson und Warshaw 2015; Heurung et al. 2014; Leroy et al. 1997), sodass es sich vermutlich um substanzspezifische Reaktionen handelt.

**Abb. 3:**
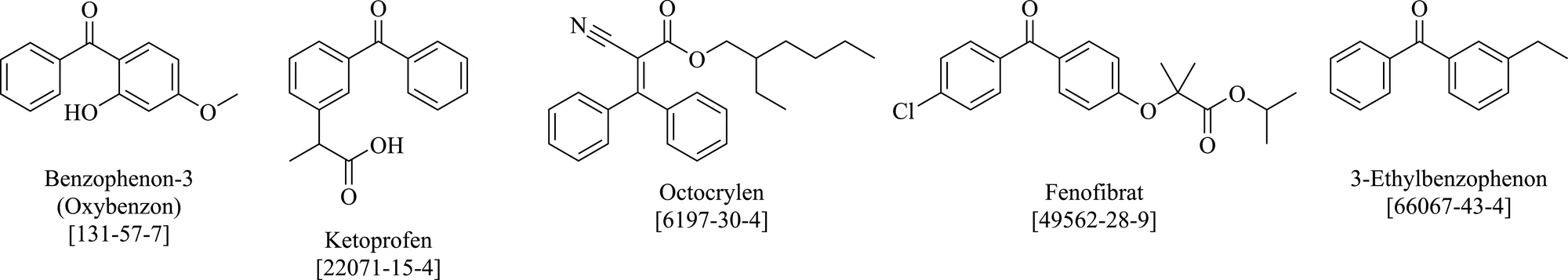
Strukturformeln und CAS-Nummern von Benzophenon-3, Ketoprofen, Octocrylen, Fenofibrat und 3-Ethylbenzophenon

Sehr häufig wird hingegen über Kreuzelizitationen bei Personen mit Ketoprofen-Sensibilisierung berichtet, einem üblicherweise topisch applizierten nichtsteroidalen Antirheumatikum zur Behandlung von Entzündungen, Muskel- und Gelenkschmerzen. Das Arzneimittel ist z. B. in Frankreich, Italien und Spanien freiverkäuflich verfügbar, wird jedoch kaum in den USA eingesetzt, sodass Sensibilisierungen in Europa viel häufiger sind als in den USA. Etwas seltener treten Kreuzelizitationen auch mit dem UV-Filter Octocrylen sowie einem Präparat mit Lipid-modifizierender Aktivität (Fenofibrat) auf. Die Strukturformeln sind in Abbildung 3 dargestellt.

In einer europäischen Studie reagierten beispielsweise 22 von 128 (17 %) der auf Ketoprofen (1%ig) positiv getesteten Personen auch auf Benzophenon-3 (10%ig). Von 41 Personen, die im Photoepikutantest positiv auf Octocrylen (5%ig oder 10%ig) getestet wurden, reagierten 18 auch auf Benzophenon-3 (10%ig) (EMCPPTS Taskforce 2012). Ergebnisse dieser und weiterer kleinerer Studien sind in de Groot und Roberts (2014) zusammengestellt und ausgewertet. In dieser Auswertung reagierten von insgesamt 37 Personen mit positivem Photoepikutantest auf Benzophenon-3 sieben ausschließlich auf Ketoprofen, drei ausschließlich auf Octocrylen, und 15 sowohl auf Ketoprofen als auch auf Octocrylen. Nur zwölf von 37 Patienten (32 %) reagierten ausschließlich auf Benzophenon-3 (de Groot und Roberts 2014). Positive (Photo-)Epikutantestreaktionen auf Benzophenon-3 könnten zumindest in Ländern mit häufigem Ketoprofen-Gebrauch teilweise auch das Ergebnis einer primären Photosensibilisierung durch Ketoprofen sein.

Die Ergebnisse verschiedener Studien mit (Photo-)Epikutantests werden unterstützt durch mehrere Einzelfälle, bei denen Personen gleichzeitig auf Benzophenon-3 und Ketoprofen reagierten (z. B. Durieu et al. 2001; Le Coz et al. 1998; Leroy et al. 1997; Milpied-Homsi 2001; Subiabre-Ferrer et al. 2019; Vigan et al. 2002), aber auch Fälle, in denen Personen eine photoallergische Kontaktdermatitis durch Benzophenon-3 im Zusammenhang mit Sonnenschutz entwickeln, die zuvor ein Ketoprofen-haltiges Gel verwendet oder darauf reagiert haben (z. B. García Castro et al. 2021; Karlsson et al. 2011; Leroy et al. 1997).

Die gleichzeitige Reaktivität auf Benzophenon-3 und Ketoprofen, Octocrylen und/oder Fenofibrat erscheint aufgrund der übereinstimmenden Benzophenon-Grundstruktur (doppelte Benzolringe, die durch eine Ketogruppe verbunden sind) grundsätzlich möglich, obwohl sich Benzophenon-3 hinsichtlich des Substitutionsmusters von Ketoprofen und seinem wichtigsten Photodegradationsprodukt (3-Ethylbenzophenon, siehe Abbildung 3), sowie von Octocrylen und Fenofibrat unterscheidet. Aufgrund der hohen und häufigen Koexposition sind Kreuzelizitationen beim Menschen häufig auf unabhängige gleichzeitige Sensibilisierungen („concomitant sensitization“) zurückzuführen. 

##### Sofortreaktionen

Berichte über Sofortreaktionen im Zusammenhang mit Benzophenon-3-haltigen Sonnenschutzmitteln sind selten (z. B. zusammengefasst in Verhulst und Goossens (2016)). In fünf Fällen wurde über am ehesten als anaphylaktoid zu interpretierende Reaktionen (generalisierte Quaddeln, niedriger Blutdruck und erhöhte Herzfrequenz) und/oder Kontakturtikaria nach Auftragen eines Sonnenschutzmittels berichtet, teilweise unmittelbar nach Auftragung und bevor die Personen Sonnenlicht ausgesetzt waren (Emonet et al. 2001; Landers et al. 2003; Spijker et al. 2008). In einem Fall traten Kontakturtikaria und Anaphylaxie zunächst bei Verwendung eines Sonnenschutzmittels auf, später auch im Zusammenhang mit Lippenpflegeprodukten und Shampoo, die Benzophenon-3 enthielten (Yesudian und King 2002). In diesen Fällen wurden im (Photo-)Epikutantest mit Benzophenon-3 (meist 10 %, teilweise nicht angegeben) nach 20 Minuten urtikarielle Reaktionen beobachtet (Emonet et al. 2001; Spijker et al. 2008; Yesudian und King 2002). Teilweise wurden die Reaktionen durch Belichtung verstärkt (Landers et al. 2003). In einem Fall wurde auch ein Hautpricktest durchgeführt, der positiv verlief (Emonet et al. 2001). Bei einer Patientin trat erst 24 Stunden nach Auftragen des Sonnenschutzmittels eine anaphylaktoide Reaktion auf und auch im Epikutantest wurde erst nach 24 Stunden eine urtikarielle Reaktion beobachtet (Tawfik und Atwater 2019). Nur in einem Fall wurde ein Assay zur Bestimmung von spezifischem IgE durchgeführt, der jedoch negativ verlief (Spijker et al. 2008).

In seltenen Fällen wurde lediglich über urtikarielle Reaktionen im Epikutantest berichtet (Berne und Ros 1998; Ramsay et al. 1972).

#### 4.4.2 Atemwegssensibilisierende Wirkung

Hierzu liegen keine Daten vor. 

### 4.5 Reproduktionstoxizität

#### 4.5.1 Fertilität

In einem systematischen Review zur Exposition gegen Benzophenon-3 in der Allgemeinbevölkerung und Reproduktionstoxizität wurden elf Humanstudien, davon vier mit Endpunkten bezüglich Spermienqualität, Fruchtbarkeit und Hormonwerten, miteinbezogen. Die Studienqualität wurde mittels des Cochrane-Risk-of-Bias-Tools von zwei unabhängigen Reviewern bewertet. Alle vier Studien (Buck Louis et al. 2014, 2015; Chen et al. 2013 a; Janjua et al. 2004) wiesen keine Assoziationen zwischen der Benzophenon-3-Exposition und den untersuchten Endpunkten auf (Ghazipura et al. 2017).

Ein detaillierter Review wertete umweltepidemiologische Studien mit Fertilitätsendpunkten aus. Es wurden acht Studien zu weiblichen bzw. fünf Studien zu männlichen Endpunkten einbezogen. Die Autoren schlussfolgern, dass es Hinweise auf Störungen des Menstruationszyklus sowie auf ein erhöhtes Risiko für Myome des Uterus und von Endometriose durch Benzophenon-3 gibt (Mustieles et al. 2023). Die untersuchten Endpunkte, Studiendesigns und Ergebnisse sind sehr unterschiedlich.

Eine Studie an 111 Paaren einer Fertilitätsklinik in Dänemark (Beck et al. 2024) wird nicht zur Bewertung herangezogen, da die Frauen sich bei der Behandlung auch unter Hormongabe befanden. Dies kann zu einer starken Verzerrung (Bias) der Ergebnisse führen.

#### 4.5.2 Entwicklungstoxizität

Die umweltepidemiologischen Studien mit Daten zur Exposition gegen Benzophenon-3 während der Schwangerschaft und zu den untersuchten entwicklungstoxischen Endpunkten sind in Tabelle 2 dargestellt.

In dem bereits erwähnten systematischen Review zeigten vier der Studien (Philippat et al. 2011, 2012; Tang et al. 2013; Wolff et al. 2008) eine statistisch signifikante Assoziation zwischen der Höhe der Urinkonzentration von Benzophenon-3 bei Schwangeren und den reproduktiven Ergebnissen. Höhere Expositionen gegen den Stoff könnten mit einem erhöhten Geburtsgewicht von Jungen sowie einem reduzierten Geburtsgewicht von Mädchen und einem erniedrigten Gestationsalter von Jungen zusammenhängen (Ghazipura et al. 2017).

**Tab. 2:** Fall-Kontroll- und Kohortenstudien mit Exposition gegen Benzophenon-3 während der Schwangerschaft

**Matrix (Messzeitpunkt),** **Konzentration,** **Land,** **(Zeitraum)**	**Kollektiv,** **Kontrollen**	**Endpunkt**	**Ergebnis**	**Adjustierung**	**Bemerkung**	**Literatur**
**Studien mit statistisch signifikant erhöhtem Risiko bzw. Effekten/Assoziationen**						
**Prospektive Kohortenstudien**						
Urin (3. Trimester), Median (25.–75. Perzentil): 7,5 (2,6–31) µg/l, USA, (1998–2002)	404 Schwangere von der Children’s Environment Health Study	Geburtsgewicht, Körperlänge, Kopfumfang, Gestationsalter	Assoziationen zwischen BP-3-Konzentrationen u. ↓ Geburtsgewicht von Mädchen u. ↑ Geburtsgewicht von Jungen	Ethnie, Geschlecht, Gestationsalter, ln-transformiertes Kreatinin, Rauchen, Bildungsstatus, Familienstand, BMI während Schwangerschaft	k. A. zu OR/RR, nur β-Koeffizienten, andere Phenole u. Phthalate untersucht	Wolff et al. 2008
Urin (vor Geburt), GM (95. Perzentil): 0,08 (0,77) µg/l, niedrig: < NG (0,04 µg/l), mittel und hoch: 2 gleichgroße Gruppen (k. w. A.), China, (2010–2012)	567 Schwangere	Geburtsgewicht, Körperlänge, Gestationsalter	Gestationsdauer: β-Koeffizient (95-%-KI): mittel exponiert: 0,11 (–0,3; 0,53); hoch exponiert: –0,45 (–0,87; –0,04); keine Assoziation bei Geburtsgewicht u. Körperlänge	Alter, Gestationsalter (nicht bei Auswertung der Gestationsdauer), BMI in später Schwangerschaft, Parität, Kreatinin, Bildungsstatus, Rauchen, Alkoholkonsum	k. A. zu OR/RR, nur Regressionskoeffizienten, andere Phenole untersucht, nur Assoziation bei Jungen statistisch signifikant, bei Mädchen nicht, Konzentrationen niedriger im Vgl. zu Werten in Europa	Tang et al. 2013
Urin (3. Trimester), Median (5.–95. Perzentil): 1,7 (0,3–143,0) µg/l, Frankreich, (2003–2006)	EDEN Kohorte, 191 Schwangere	Geburtsgewicht, Körperlänge, Kopfumfang	pos. Assoziation zwischen Anstieg der ln-transformierten BP-3-Konzentrationen um eine Einheit u. Geburtsgewicht (26 g, 95-%-KI: −2; 54) u. Kopfumfang (0,1 cm, 95-%-KI: 0,0; 0,2)	maternales Alter, BMI vor der Schwangerschaft, Parität, Jahr der Probenahme, Bildungsstatus, aktuelle Beschäftigung, aktives Rauchen, Rekrutierungsort	auch andere Phenole u. Phthalate untersucht, k. A. zu OR/RR, nur multivariate lineare Regressionsanalyse, nur Jungen untersucht	Philippat et al. 2012
Urin (4 Proben), Median (25.–75. Perzentil): 46,5 (14,0–179,0) µg/l, USA, (2006–2008)	LIFECODES Geburtskohorte, 476 Schwangere	Geburtsgewicht, Kopfumfang, abdominaler Umfang, Femurlänge	Assoziation zwischen BP-3-Konzentration u. ↓ Abdominalumfang bei Jungen, kein Effekt für Geburtsgewicht z-Score	maternales Alter, Ethnie, BMI während Schwangerschaft, Krankenversicherung, Bildungsstatus, Geschlecht, IVF, Parität, Rauchen, Alkoholkonsum	auch andere Phenole untersucht, k. A. zu OR/RR, nur generalisierte lineare Modelle	Ferguson et al. 2018
Urin (23.–29. Schwangerschaftswoche), Median (5.–95. Perzentil): 2,23 (0,22–79,4) µg/l, Frankreich, (2003–2006)	EDEN Kohorte, 473 Schwangere	Geburtsgewicht, Plazenta-Fetal-Quotient	pos. Assoziation zwischen ln-transformierten BP-3-Konzentrationen u. Geburtsgewicht (21 g, 95-%-KI: −3,54; 45,5)	Gestationsalter, maternales aktives u. passives Rauchen, maternales Alter, KG u. Körpergröße, Bildungsstatus, Parität, Rekrutierungsort	auch andere Phenole u. Phthalate untersucht, k. A. zu OR/RR, nur Regressionsmodell, nur Jungen untersucht	Philippat et al. 2019
Urin (3 Zeitpunkte), Median (25.–75. Perzentil): 26,5 (10,36–124,85) µg/l, Mexiko, (2011–2017)	PROTECT Kohorte, 922 Schwangere	vorzeitige Geburt, Gestationsalter, klein/groß für Gestationsalter	Assoziation zwischen BP-3-Konzentration u. Gestationsalter (1,9 d ↑, 95-%-KI: 0,54; 3,26)	spezifisches Gewicht Urin, maternales Alter, Versicherungsstatus, Alkoholkonsum, Passivrauchen	auch andere Phenole u. Parabene untersucht, k. A. zu OR/RR, Spearmans-Korrelationsanalysen	Aker et al. 2019
Urin (3 Zeitpunkte), 3. Trimester: Median (25.–75. Perzentil): 0,44 (NG–1,30) µg/l, China, (2014–2015)	Women and Children Medical and Healthcare Center of Wuhan, 847 Schwangere	Geburtsgewicht, Körperlänge	Assoziation zwischen ln-transformierter BP-3-Konzentration im 3. Trimester u. ↓ Geburtsgewicht bei Mädchen (–19,75 g, 95-%-KI: –37,31; –2,19) u. ↓ Körperlänge (–0,08 cm; –0,15; –0,02)	Gestationsalter, KG-Zunahme während Schwangerschaft, BMI vor Schwangerschaft, Parität, Bildungsstatus, Passivrauchen, paternale Körpergröße	auch andere Benzophenone untersucht, k. A. zu OR/RR, multiple lineare Regressionsmodelle, kein Effekt bei Jungen	Long et al. 2019
**Fall-Kontroll-Studien**						
Urin, niedrig: < NG (0,04 µgl), mittel: > 0,04–0,1 µg/l, hoch: > 0,1 µg/l, China, (2009–2014)	101 Mütter von Kindern mit Hirschsprung-Erkrankung, 322 altersangepasste Kontrollen	Hirschsprung-Erkrankung	adj. OR (niedrige Exposition als Referenzgruppe, 129 Kontrollen/37 Fälle): adj. OR 1, mittlere Exposition (97 Kontrollen/33 Fälle): adj. OR 2,39 (95-%-KI: 1,10–5,21), hohe Exposition (96 Kontrollen/31 Fälle): adj. OR 2,61 (95-%-KI: 1,15–5,92)	maternaler BMI, Kreatinin	Raucherstatus u. Alkoholkonsum kein statistisch signifikanter Unterschied bei Müttern der Fälle bzw. der Kontrollen, Dosis-Wirkungs-Beziehung, geringe Fallzahl	Huo et al. 2016
**Querschnittsstudien**						
Serum (k. A.), Median (25.–75. Perzentil): 0,545 (0,332–1,049) µg/l, China, (2022)	600 Schwangere rekrutiert im Krankenhaus von Shenzhen City	Geburtsgewicht, Brustumfang, Kopfumfang u. Körperlänge bei Geburt	pos. Assoziationen zwischen BP-3 u. Geburtsgewicht u. Brustumfang	maternales Alter, Bildungsstatus, BMI vor Schwangerschaft, Wohnort	auch andere Benzophenone untersucht, Raucherstatus u. Alkoholkonsum nicht abgefragt, keine OR/RR, nur Spearmans-Korrelationsanalyse	Fu et al. 2024
**Studien ohne statistisch signifikant erhöhtes Risiko (OR, RR, HR) bzw. ohne Assoziationen**						
**Prospektive Kohortenstudien**						
LIFE-Studie, Urin, GM (95-%-KI), Maternale Konzentration: Geburt Junge: 8,65 (5,66–13,2) µg/l, Geburt Mädchen: 7,68 (5,08–11,6) µg/l, Paternale Konzentration: Geburt Junge: 7,47 (4,94–11,3) µg/l, Geburt Mädchen: 5,54 (3,86–7,95) µg/l, Südkorea, (2005–2009)	220 Paare mit Einlingsgeburt	Verhältnis Jungen/Mädchen	keine Assoziation, adj. RR: maternal: 1,05 (95-%-KI: 0,91–1,21), paternal: 1,11 (95-%-KI: 0,98–1,24)	Kreatinin, Untersuchungsort, Alter, Haushaltseinkommen, Parität	andere Phenole untersucht: Assoziationen zwischen 4-OH-BP im maternalen Urin u. höherem Anteil an Geburten von Jungen sowie zwischen BP-2 im maternalen od. im paternalen Urin u. höherem Anteil an Geburten von Mädchen	Bae et al. 2016
Urin, Median (Min.–Max.): 0,28 (NG–2,57) µg/l, Dänemark, (2012–2014)	183 Schwangere	Geburtsgewicht, Kopfumfang, Abdominalumfang	keine Assoziation	Gestationsalter, BMI vor Schwangerschaft	andere Benzophenone untersucht, k. A. zu OR/RR, generalisierte lineare Modelle	Krause et al. 2018
Urin, Median (25.–75. Perzentil): 137,6 (55,0–377) µg/l, USA, (2004–2018)	EARTH Study, 346 Schwangere	Geburtsgewicht, Kopfumfang	keine Assoziation	maternales u. paternales Alter, BMI, maternale Bildung, maternales u. paternales Rauchen, IVF, Jahreszeit	andere Phenole untersucht	Messerlian et al. 2018
**Fall-Kontroll-Studien**						
Urin, GM (95-%-KI), Maternale Konzentration: Fall: 0,14 (0,10–0,21) µg/l, Kontrolle: 0,09 (0,07–0,12) µg/l, Paternale Konzentration: Fall: 0,08 (0,05–0,11) µg/l, Kontrolle: 0,07 (0,06–0,08) µg/l, gering: < NG (0,04 µg/l), hoch: > 0,04 µg/l, China, (k. A. zum Zeitraum)	70 Paare mit Spontanabort, 180 Kontrollpaare mit mind. einem lebenden Kind u. ohne Spontanaborte (aus Gesamtbevölkerung u. nicht aus Krankenhaus)	Spontanabort	keine Assoziation, adj. OR (hoch gegen gering exponiert): maternal: 0,77 (95-%-KI: 0,36–1,67), paternal: 1,12 (95-%-KI: 0,61–2,04)	paternales Rauchen, maternaler BMI, maternaler Alkoholkonsum, Kreatinin	weitere Phenole untersucht mit Assoziationen bei Pentachlorphenol im paternalen Urin, 4-n-Octylphenol im maternalen Urin bzw. 4-n-Nonylphenol im maternalen Urin, k. A. wie die Kontrollpaare rekrutiert wurden („zufällig aus Bevölkerung ausgewählt“)	Chen et al. 2013 b
Urin, maternal, 1. Tertil: < 0,7 µg/l (19 Fälle, 43 Kontrollen), 2. Tertil: 0,7–2,7 µg/l (12 Fälle, 30 Kontrollen), 3. Tertil: ≥ 2,7 µg/l (7 Fälle, 40 Kontrollen), Frankreich, (2002–2006)	EDEN u. PELAGIE Geburtskohorte mit 50 Müttern von Kindern mit Hodenhochstand, 206 Müttern mit Kindern ohne diesen Befund; nicht von allen Urinmessdaten	Hodenhochstand	keine Assoziation, adj. OR: 2. Tertil: 0,87 (95-%-KI: 0,3–2,5), 3. Tertil: 0,51 (95-%-KI: 0,2–1,5)	maternales Alter, Parität, Bildungsstand, Schwangerschaftsdauer, Kreatinin	weitere Phenole untersucht, geringe Fallzahl	Chevrier et al. 2012
Plazenta, Median (Min.–Max.): Fälle: < NG (NG–1,28) ng/g Gewebe, Kontrollen: < NG (0,65–3,09) ng/g Gewebe, Spanien, (2000–2002)	28 Mütter von Kindern mit Hodenhochstand od. Hypospadie, 51 Mütter mit Kindern ohne diesen Befund	Hodenhochstand od. Hypospadie	keine Analysen durchgeführt, da BP-3 nur in 4 % der Proben nachweisbar	maternales Alter, Geburtsgewicht	weitere Phenole u. Bisphenol A untersucht, geringe Fallzahl	Fernández et al. 2016

adj.: adjustiert; BMI: Body-Mass-Index; BP-2: Benzophenon-2; BP-3: Benzophenon-3; GM: geometrisches Mittel; HR: Hazard Ratio; IVF: In-vitro-Fertilisation; KI: Konfidenzintervall; LIFE: Longitudinal Investigation of Fertility and the Environment; ln-transformiert: natürlicher Logarithmus des Werts; MW: Mittelwert; NG: Nachweisgrenze; 4-OH-BP: 4-Hydroxybenzophenon; OR: Odds-Ratio; pos: positiv; RR: relatives Risiko; Vgl. Vergleich; z-Score: Maß für Abstand (Zahl an Standardabweichungen) eines Datenpunktes zum Mittelwert der Verteilung

Die zahlreichen epidemiologischen Studien unterschiedlichen Studientyps zeigen widersprüchliche Ergebnisse. 

Bei prospektiven Kohortenstudien wurden Assoziationen zwischen den Benzophenon-3-Konzentrationen im Urin der Mütter und einem erniedrigten Geburtsgewicht von Mädchen bzw. einem erhöhten Geburtsgewicht von Jungen (Wolff et al. 2008), einem erhöhten Gestationsalter bei Jungen (Tang et al. 2013), einem erhöhten Geburtsgewicht bei Jungen (Philippat et al. 2012), einem erniedrigten Abdominalumfang bei Jungen (Ferguson et al. 2018), einem erhöhten Geburtsgewicht bei Jungen (Philippat et al. 2019), einem erhöhten Gestationsalter (Aker et al. 2019) und einem erniedrigten Geburtsgewicht (Long et al. 2019) festgestellt. Eine Fall-Kontroll-Studie ergab ein statistisch signifikant erhöhtes Risiko für das Auftreten der Hirschsprung-Erkrankung (angeborenes aganglionäres Megakolon) bei erhöhten maternalen Benzophenon-3-Konzentrationen im Urin (Huo et al. 2016). In einer Querschnittsstudie zeigte sich eine Assoziation zwischen den maternalen Benzophenon-3-Konzentrationen im Serum und einem erniedrigten Geburtsgewicht und Brustumfang (Fu et al. 2024). In einer prospektiven Kohortenstudie wurde der BMI bei Kindern im Alter von zwei Jahren untersucht (Lv et al. 2024). Dieser Zeitpunkt kann nicht mehr nur auf die In-utero-Exposition zurückgeführt werden und wird daher nicht zur Bewertung herangezogen.

In prospektiven Kohortenstudien wurden keine Assoziationen zwischen maternalen Benzophenon-3-Konzentrationen im Urin und dem Geschlechterverhältnis (Bae et al. 2016), dem Geburtsgewicht, Kopfumfang und Abdominalumfang (Krause et al. 2018; Messerlian et al. 2018) gefunden. Auch bei zwei Fall-Kontroll-Studien ergaben sich keine Assoziationen zwischen den maternalen Benzophenon-3-Konzentrationen im Urin und Spontanaborten (Chen et al. 2013 b) oder Hodenhochstand (Chevrier et al. 2012). In einer Fall-Kontroll-Studie konnten aufgrund der geringen Fallzahl keine Analysen vorgenommen werden (Fernández et al. 2016).

Eine Fall-Kontroll-Studie liegt nur als Zusammenfassung eines Kongressbeitrags vor (Philippat et al. 2011). Da zahlreiche Informationen fehlen, wird die Studie nicht zur Bewertung herangezogen.

**Fazit**: In der Mehrzahl der umweltepidemiologischen Studien lagen die Benzophenon-3-Konzentrationen im Urin der untersuchten Frauen im Bereich der Hintergrundbelastung (Kreatinin-adjustierte geometrische Mittelwerte in USA: 18 bis 45 µg/l Urin; French 1992; NTP 2020). Aus den Studien, in denen die gemessenen Konzentrationen von Benzophenon-3 im Urin über die Hintergrundbelastung hinausgingen, lassen sich Hinweise auf eine Assoziation zwischen erhöhten maternalen Benzophenon-3-Konzentrationen im Urin und einem erhöhten Geburtsgewicht und Kopfumfang bei Jungen (Philippat et al. 2012, 2019) und einem erhöhten Gestationsalter (Aker et al. 2019) ableiten. Die Studie mit dem erniedrigten Abdominalumfang bei Jungen (Ferguson et al. 2018) passt nicht gut zu den erhöhten Körpergewichten.

### 4.6 Genotoxizität

Hierzu liegen keine Daten vor.

### 4.7 Kanzerogenität

Ein Zusammenhang zwischen einer selbstberichteten jemals erfolgten Diagnose hormonell abhängiger Krebsarten und gemessenen Konzentrationen (als Interquartil-Abstand (IQR)) bestimmter Chemikalien (per- und polyfluorierte Alkylverbindungen, Phenole, Parabene) wurde mit logistischen Regressionsmodellen geprüft. Die Daten stammten aus dem Biomonitoringprogramm National Health and Nutrition Examination Survey (NHANES) der USA. Einbezogen wurden Personen ab einem Alter von 20 Jahren unter Berücksichtigung von Alter, ethnischer Zugehörigkeit, Geschlecht und Sozialstatus. Für Benzophenon-3 errechnete sich in einigen Fällen ein positiver Zusammenhang für Eierstockkrebs (Odds Ratio (OR): 1,76, 95-%-Konfidenzintervall (KI): 1,00; 3,09; marginal signifikant (0,05 ≤ p < 0,1)), für Melanome bei Frauen (OR: 1,81; 95-%-KI: 1,10; 2,96), für Brustkrebs bei Frauen (OR hispanischer Frauen: 3,03; 95-%-KI: 1,22; 7,50; OR weißhäutige Frauen: 0,94, 95-%-KI: 0,67; 1,31) und für Prostatakrebs bei Männern (OR weißhäutige Männer: 1,42; 95-%-KI: 1,07; 1,89; OR dunkelhäutige Männer: 0,70; 95-%-KI: 0,41; 1,21; p = 0,022) (Cathey et al. 2023). Die Messung von Benzophenon-3 wurde erst nach erfolgter Krebsdiagnose durchgeführt. Benzophenon-3 wird innerhalb von drei Tagen zu mindestens 99 % ausgeschieden, akkumuliert also nicht im Körper (siehe Abschnitt 3.1). Daher kann aus dieser Untersuchung nicht geschlossen werden, dass Benzophenon-3 die Ursache der entsprechenden Krebsart ist.

## 5 Tierexperimentelle Befunde und In-vitro-Untersuchungen

### 5.1 Akute Toxizität

#### 5.1.1 Inhalative Aufnahme

Hierzu liegen keine Untersuchungen vor.

#### 5.1.2 Orale Aufnahme

Die orale LD_50_ für Benzophenon-3 lag bei je fünf männlichen und weiblichen Wistar-Ratten und Schlundsondengabe in wässriger 0,5%iger Carboxymethylzellulose-Suspension oberhalb von 12 800 mg/kg KG. Direkt nach der Applikation waren die Tiere schläfrig, weitere auch histopathologische Befunde traten nicht auf (ECHA 2013).

Bei Sherman-Ratten (je fünf pro Dosis, k. A. zum Geschlecht) lag die orale LD_50_ für Benzophenon-3 in Maiskeimöl bei Schlundsondengabe berechnet bei 11 600 mg/kg KG (2, 3, 4 Tiere gestorben bei 10 000, 12 500 und 16 000 mg/kg KG). Es wird von keinen weiteren Befunden berichtet (ECHA 2013).

#### 5.1.3 Dermale Aufnahme

Die dermale LD_50_ von Benzophenon-3 lag bei Kaninchen (k. w. A.) oberhalb von 16 000 mg/kg KG. Die Haut war an der Applikationsstelle leicht bis mäßig gerötet (NTP 2020).

Auch bei männlichen Albino-Kaninchen lag die dermale LD_50_ nach 18- bis 22-stündiger semiokklusiver Auftragung der pulverförmigen Testsubstanz auf die geschorene Rückenhaut und fünftägiger Nachbeobachtung oberhalb von 16 000 mg Benzophenon-3/kg KG. Nur bei der niedrigsten Dosis von 2000 mg/kg KG trat in den ersten beiden Tagen bei zwei von fünf Tieren eine leichte Rötung an der Applikationsstelle auf, bei keinem der Tiere traten histopathologische Befunde auf (ECHA 2013).

#### 5.1.4 Intraperitoneale Aufnahme

Die intraperitoneale LD_50_ für Benzophenon-3 lag bei 21 männlichen und 21 weiblichen NMRI-Mäusen oberhalb von 1600 mg/kg KG, da nur bei der Dosis 800 mg/kg KG ein Tier starb. Benzophenon-3 wurde in wässriger 0,5%iger Carboxymethylzellulose-Suspension appliziert (ECHA 2013).

### 5.2 Subakute, subchronische und chronische Toxizität

#### 5.2.1 Inhalative Aufnahme

Hierzu liegen keine Untersuchungen vor.

#### 5.2.2 Orale Aufnahme

Die Befunde der Studien mit wiederholter oraler Aufnahme von Benzophenon-3 sind in Tabelle 3 aufgeführt.

##### 5.2.2.1 Ratten

Je fünf bzw. zehn F344-Ratten pro Dosierung und Geschlecht erhielten zwei und 13 Wochen lang mit dem Futter Benzophenon-3. Die statistischen Signifikanzen wurden in der 2-Wochen-Studie anhand des Student-Tests, in der 13-Wochen-Studie anhand des Dunnett-Tests ermittelt. Die Dosierungen betrugen in der **2-Wochen**-Studie 0, 295/311, 589/564, 1159/1104, 2259/2218, 4210/3527 mg/kg KG und Tag für männliche bzw. weibliche Tiere. Es traten ab der niedrigsten Dosis von 295/311 mg/kg KG und Tag erhöhtes absolutes und relatives Lebergewicht (siehe Tabelle 3) und ab 589/564 mg/kg KG und Tag deutliche Vakuolisierungen im Zytoplasma der Hepatozyten auf. Das absolute und relative Nierengewicht war bei männlichen Tieren ab 589 mg/kg KG und Tag etwas erhöht, bei 4210 mg/kg KG und Tag statistisch signifikant, und es traten Dilatationen im Cortex, Regeneration des Epithels und Nekrosen in der Papille auf. In der **13-Wochen**-Studie erhielten männliche bzw. weibliche Ratten 0, 213/196, 429/393, 875/780, 1805/1599, 3656/3261 mg/kg KG und Tag. Das relative Lebergewicht war bei männlichen und weiblichen Tieren ab der niedrigsten Dosis statistisch signifikant zwischen 10 % und 14 % ohne histopathologisches Korrelat erhöht. Bei männlichen Tieren war ab 1805 mg/kg KG und Tag, bei weiblichen Tieren ab 393 mg/kg KG und Tag das Körpergewicht statistisch signifikant verringert. Das absolute und relative Nierengewicht war bei den weiblichen Tieren ab 1599 mg/kg KG und Tag und bei den männlichen in der höchsten Dosis statistisch signifikant erhöht (siehe Tabelle 3). Das absolute und relative Nierengewicht nahm bei den exponierten männlichen Tieren dosisabhängig zu und war nach den Autoren erst bei der höchsten Dosis statistisch signifikant erhöht. Es traten in den Nieren bei männlichen Tieren ab 875 mg/kg KG und Tag und den weiblichen Tieren in der höchsten Dosisgruppe erweiterte Tubuli und Regeneration im Tubulusepithel, bei der höchsten Dosis bei männlichen und weiblichen Tieren Nekrose in der Papille und Entzündung mit Fibrose auf. Bei der höchsten Dosis war auch das spezifische Uringewicht bei männlichen Tieren sowie die Spermienzahl in den Nebenhoden vermindert und bei weiblichen Tieren der Östruszyklus verlängert (French 1992). Da das relative Nierengewicht der männlichen Tiere bereits bei der niedrigsten Dosis um 11 % erhöht war, wird dies als beginnender adverser Effekt gewertet. Bei der höchsten Dosierung betrug das relative Nierengewicht das 2,5-Fache der Kontrolle. Die Dosierung von 393 mg/kg KG und Tag war aufgrund des verminderten Körpergewichtes weiblicher Tiere ein LOAEL.

Bei trächtigen Sprague-Dawley-Ratten, deren Nachkommen für eine Kanzerogenitätsstudie eingesetzt wurden, trat ab der mittleren Dosis von 206 mg/kg KG und Tag eine ca. 9%ige verminderte Körpergewichtszunahme während der Trächtigkeit auf. Es fanden keine weiteren Untersuchungen der 39 Tage lang exponierten F0-Muttertiere statt. Bei den F1-Tieren, mit denen eine Kanzerogenitätsstudie durchgeführt wurde, wurden vor allem (prä)neoplastische Befunde beschrieben (siehe Abschnitt 5.7) (NTP 2020).

In einer Untersuchung von 1972 wurde Benzophenon-3 **zwölf Wochen** lang an männliche und weibliche Wistar-Ratten in Dosierungen von 0, 70/70, 320/330, 2100/2100, 4700/5300 mg/kg KG und Tag verabreicht (siehe Tabelle 3). Trotz ähnlicher Futtermengenaufnahme war die Körpergewichtszunahme in den beiden hohen Dosisgruppen bei männlichen Tieren um 20 % bzw. 35 %, bei weiblichen um 25 % bzw. 35 % vermindert. Bei weiblichen Tieren waren ab 2100 mg/kg KG und Tag Hämoglobin, Leukozyten- und Neutrophilenzahlen vermindert sowie die Lymphozytenzahl erhöht und einige relative Organgewichte verändert (siehe Tabelle 3). Bei der höchsten Dosis waren bei drei männlichen und fünf weiblichen Tieren die Nieren geschwollen und wiesen Umbildungen an den Polen auf. Die histopathologische Untersuchung ergab Veränderungen am Tubulusepithel der Niere mit fortschreitender Degeneration, blasiger Entartung der Tubuli bis zur Wucherung des interstitiellen Gewebes und Infiltration von Leukozyten. Anfänge einer Degeneration in der Niere waren bei vier weiblichen Tieren der 2100-mg/kg KG und Tag-Gruppe zu beobachten (Lewerenz et al. 1972).

##### 5.2.2.2 Mäuse

B6C3F1-Mäusen wurde zwei und 13 Wochen lang oral Benzophenon-3 verabreicht und statistische Signifikanzen in der 2-Wochen-Studie anhand des Student-Tests, in der 13-Wochen-Studie anhand des Dunnett-Tests ermittelt. Die oralen Dosierungen betrugen 0, 992/1050, 1752/2330, 3947/4914, 7438/9859, 18 624/22 968 mg/kg KG und Tag für männliche bzw. weibliche Tiere in der **2-Wochen**-Studie. Erhöhtes Lebergewicht trat bei den weiblichen Tieren ab 1050 mg/kg KG und Tag und bei den männlichen Tieren ab 1752 mg/kg KG und Tag auf. Ab dieser Dosis kam es bei beiden Geschlechtern zu deutlichen Vakuolisierungen im Zytoplasma der Hepatozyten. In der **13-Wochen**-Studie bei Dosierungen von 0, 480/629, 1068/1425, 2487/3232, 5981/7579, 13 937/18 539 mg/kg KG und Tag für männliche bzw. weibliche Tiere zeigten sich keine klinischen Symptome. Das relative und absolute Lebergewicht war ab 1068/1425 mg/kg KG und Tag bei beiden Geschlechtern dosisabhängig leicht erhöht. Der Anstieg des relativen Nierengewichtes der weiblichen Tiere war nicht dosisabhängig. Bei den männlichen Tieren zeigte sich kein Trend bei den Nierengewichten, die in der niedrigsten und höchsten Dosisgruppe leicht reduziert, in den anderen Dosisgruppen eher erhöht waren. Das Körpergewicht war ab der mittleren Dosis geringer als der Kontrollwert. Histopathologische Befunde traten bei den beiden hohen Dosisgruppen in Form einer minimalen zytoplasmatischen Vakuolisierung der Hepatozyten auf. In der Hochdosisgruppe kam es bei männlichen Tieren zu einer Abnahme der Spermienzahl in den Nebenhoden, leicht erhöhter Zahl an abnormalen Spermien und eosinophilen Proteinzylindern leichten Grades in den Nierentubuli der inneren Medulla. Wenn diese auftraten, kam es auch zu leichter Dilatation und entzündlichen Zellinfiltraten in den Tubuli. Bei weiblichen Tieren war der Östruszyklus in der höchsten Dosisgruppe verlängert (French 1992). Diese Befunde traten in einer Reproduktionsstudie mit CD1-Mäusen nicht auf (NTP 1990).

In der Zwei-Jahre-Studie mit B6C3F1-Mäusen war das Körpergewicht in der höchsten Dosisgruppe von 1207/1278 mg/kg KG und Tag um 10 % vermindert, es traten zytoplasmatische Veränderungen in den Nierentubuli, ab 339/320 mg/kg KG und Tag Pigmente in Knochenmark und Milz sowie ab 113/109 mg/kg KG und Tag mehrkernige Riesenzellen in der Leber auf. Auch kam es bei der höchsten Dosierung bei fünf weiblichen Tieren zu knöchernen Metaplasien in der Niere, was statistisch signifikant war (in Kontrolle kein Tier). Diese Läsionen bestanden aus kleinen Bereichen mit reifen Knochen innerhalb des Kortex der Niere. Die knöcherne Metaplasie wird als spontane Läsion mit unbekannter pathologischer Bedeutung betrachtet. Der NOAEL lag bei der niedrigsten Dosis von 113/109 mg/kg KG und Tag (NTP 2020).

**Tab. 3:** Wirkung von Benzophenon-3 nach wiederholter oraler Verabreichung

**Stamm, Anzahl pro Gruppe**	**Exposition**	**Befunde**	**Literatur**
**Ratte**, F344/N, 5 ♂, 5 ♀	**2 Wochen**, 0, 3125, 6250, 12 500, 25 000, 50 000 mg/kg Futter (0, 295/311, 589/564, 1159/1104, 2259/2218, 4210/3527 mg/kg KG u. d für ♂/♀)	**ab 295** **mg/kg KG**: ♂: abs. (24 %) u. rel. (14 %) Lebergewicht ↑; **ab 589/564** **mg/kg KG**: zytoplasmatische Vakuolisierung der Hepatozyten, ♀: abs. (17 %) u. rel. (21 %) Lebergewicht ↑, ♂: rel. (9 %) Nierengewicht ↑ dosisabhängig; **4210/3527** **mg/kg KG**: Futterkonsum 18 % bzw. 20 % ↓, ♀: Mortalität 1/5, ♂: KG-Zunahme 12 % ↓, rel. (19 %) Nierengewicht ↑, fokale Dilatationen in renalem Cortex und/oder Medulla (4/5), Regeneration renales Tubulusepithel (3/5), fokale Nekrosen Nierenpapille (1/5)	French 1992
**Ratte**, Sprague Dawley, F0: 42 ♀ (0), 35 ♀ (1000 mg/kg Futter), 35 ♀ (3000 mg/kg Futter), 43 ♀ (10 000 mg/kg Futter)	**39 Tage**, 0, 1000, 3000, 10 000 mg/kg Futter (während Trächtigkeit: 0, 70, 206, 660 mg/kg KG u. d, während 1.–14. Laktationstag: 0, 157, 478, 1609 mg/kg KG u. d), Reinheit: > 99 %, 7 d/Wo	**206 mg/kg KG**: KG-Zunahme 9 % ↓; **660** **mg/kg KG**: während Trächtigkeit Futterkonsum ↓, KG 3 % ↓	NTP 2020
**Ratte**, Wistar, 12 ♂, 12 ♀	**12 Wochen**, 0; 0,02; 0,1; 0,5; 1 % im Futter (0, 70/70, 320/330, 2100/2100, 4700/5300 mg/kg KG u. d für ♂/♀)	rel. Organgewichte auf Gehirngewicht bezogen; **ab 2100** **mg/kg KG**: KG 20 % ↓ (♂) bzw. 25 % ↓ (♀), rel. Thymusgewicht ↓, rel. Herzgewicht ↓, ♀: Lymphozyten ↑, Neutrophile ↓, Hämoglobin ↓, Leukozyten ↓, rel. Schilddrüsengewicht ↑, beginnende Nierendegeneration (4/12); **4700/5300** **mg/kg KG**: KG 35 % ↓ (♂, ♀), rel. Hypophysengewicht ↓, rel. Nebennierengewicht ↓, geschwollene Nieren (3 ♂, 5 ♀): Veränderungen am Nierentubulusepithel, Degeneration, blasige Entartung der Tubuli, Wucherung des interstitiellen Gewebes, Infiltration von Leukozyten, degenerative Nephrose, ♀: rel. Lungengewicht ↓, rel. Milzgewicht ↓	Lewerenz et al. 1972
**Ratte**, F344/N, 10 ♂, 10 ♀	**13 Wochen**, 0, 3125, 6250, 12 500, 25 000, 50 000 mg/kg Futter (0, 213/196, 429/393, 875/780, 1805/1599, 3656/3261 mg/kg KG u. d für ♂/♀)	**ab 213/196** **mg/kg KG**: **NOAEL**, gelb-grünlicher Urin, abs. (14/11 %) u. rel. (14/10 %) Lebergewicht ↑ ohne histopathologisches Korrelat, ♂: abs. u. rel. Nierengewicht ↑ (10 bzw. 11 % n. s.) ohne histopathologisches Korrelat; **ab 393** **mg/kg KG**: ♀: KG 14 % ↓; **ab 875** **mg/kg KG**: Dilatation renaler Nierentubuli, Schweregrad Regeneration Tubulusepithelzellen ↑, alk. Phosphatase ↓; **ab 1805/1599** **mg/kg KG**: ♂: KG-Zunahme 18 % ↓, Thrombozytenzahl ↑, ♀: abs. (9 %) u. rel. (12 %) Nierengewicht ↑; **3656/3261** **mg/kg KG**: Dilatation renaler Nierentubuli, Entzündung mit Fibrose im Niereninterstitium, Nekrosen in Nierenpapille, Zellinfiltrate mit Neutrophilen u. Lymphozyten u. Makrophagen, Urinvolumen ↑, spezifisches Uringewicht ↓, Gamma-Glutamyltransferase im Blut ↑, ♀: Östruszyklus verlängert, Regeneration Tubulusepithelzellen ↑, ♂: spezifisches Uringewicht ↓, abs. (67 %) u. rel. (160 %) Nierengewicht ↑, Spermienzahl in den Nebenhoden ↓, keine Befunde in Blase	French 1992
**Maus**, B6C3F1, 5 ♂, 5 ♀	**2 Wochen**, 0, 3125, 6250, 12 500, 25 000, 50 000 mg/kg Futter (0, 992/1050, 1752/2330, 3947/4914, 7438/9859, 18 624/22 968 mg/kg KG u. d)	**ab 1050 mg/kg KG**: ♀: abs. (29 %) u. rel. Lebergewicht ↑ (23 %); **ab 1752/2330 mg/kg KG**: abs. (12/44 %) u. rel. (14/33 %) Lebergewicht dosisabhängig ↑, zytoplasmatische Vakuolisierung der Hepatozyten; **ab 7438/9859 mg/kg KG**: ♂: rel. Nierengewicht 8 % ↓, ♀: KG-Zunahme 23 % ↓	French 1992
**Maus**, B6C3F1, 10 ♂, 10 ♀	**13 Wochen**, 0, 3125, 6250, 12 500, 25 000, 50 000 mg/kg Futter (0, 480/629, 1068/1425, 2487/3232, 5981/7579, 13 937/18 539 mg/kg KG u. d für ♂/♀)	**480/629** **mg/kg KG**: **NOAEL**; **ab 1068/1425** **mg/kg KG**: abs. (14/16 %) u. rel. (11/14 %) Lebergewicht dosisabhängig ↑, ♀: rel. Nierengewicht ↑ (18 %, 2 %, 16 %, 5 %), ♂: rel. Nierengewicht ↑ (1068 und 5981 mg/kg KG: 12 % bzw. 15 %) und ↓ (480 und 13 937 mg/kg KG: 5 % bzw. 4 %); **ab 2487/3232** **mg/kg KG**: ♂: KG-Zunahme dosisabhängig ↓ (hier 7 %); **ab 5981/7579** **mg/kg KG**: minimale zytoplasmatische Vakuolisierung der Hepatozyten, ♀: KG ↓ (hier 8 %); **13 937/18 539 mg/kg KG**: ♂: Spermienzahl ↓, Zahl abnormaler Spermien ↑, eosinophile Proteinzylinder leichten Grades in Nierentubuli innerer Medulla, wenn vorhanden dann auch leichte Dilatation und entzündliche Zellinfiltrate, ♀: Östruszyklus verlängert	French 1992
**Maus**, B6C3F1, 50 ♂, 50 ♀	**2 Jahre**, 0, 1000, 3000, 10 000 mg/kg Futter (0, ca. 113/109, 339/320, 1207/1278 mg/kg KG u. d für ♂/♀), Reinheit: > 99 %	**ab 113/109** **mg/kg KG**: Pigment im Knochenmark (9/96, 2/98, 9/98, 100/100 bei Kontrolle und Dosisgruppen), Leber mit mehrkernigen Riesenzellen (♂: 2/49, 39/50, 45/50, 48/50 bei Kontrolle und Dosisgruppen), **NOAEL**; **ab 339/320** **mg/kg KG**: Pigment in Milz (16/97, 15/100, 46/98, 55/100 bei Kontrolle und Dosisgruppen), ♀: KG-Zunahme ≥ 10 % ↓; **1207/1278** **mg/kg KG**: ♂: KG-Zunahme > 10 % ↓, zytoplasmatische Veränderungen in Nierentubuli (0/48, 0/50, 0/50, 46/50 bei Kontrolle und Dosisgruppen), ♀: knöcherne Metaplasien in der Niere (5/50)	NTP 2020

abs: absolut; d: Tag; n. s.: nicht statistisch signifikant; rel.: relativ; u: und

##### 5.2.2.3 Fazit

In einer 13-Wochen-Fütterungsstudie mit F344-Ratten lag der NOAEL bei 213 bzw. 196 mg/kg KG und Tag. Bei der nächst höheren Dosis von 393 mg/kg KG und Tag war das Körpergewicht der weiblichen Tiere um 14 % vermindert, bei der höchsten Dosis von 3656 bzw. 3261 mg/kg KG und Tag traten Nierenbefunde wie Dilatationen im Tubulus (bei männlichen bereits ab 875 mg/kg KG und Tag) oder Nekrosen in der Papille sowie bei männlichen Tieren eine verminderte Spermienzahl und bei weiblichen ein verlängerter Östruszyklus auf. Auch bei B6C3F1-Mäusen war in der 13-Wochen-Fütterungsstudie das Lebergewicht ab der zweitniedrigsten Dosis von 1068 bzw. 1425 mg/kg KG und Tag um 11–16 % ohne weitere histopathologische Befunde erhöht (LOAEL). Ab dieser Dosis war auch das Nierengewicht verändert, bei weiblichen Tieren erhöht, bei männlichen nicht dosisabhängig erhöht, histopathologische Befunde traten in den Nieren nicht auf. Bei Mäusen lag der NOAEL in der 13-Wochen-Fütterungsstudie bei 480 bzw. 629 mg/kg KG und Tag (French 1992). In der Zwei-Jahre-Studie mit B6C3F1-Mäusen war die Körpergewichtszunahme in der höchsten Dosisgruppe von 1207/1278 mg/kg KG und Tag um 10 % vermindert, es traten zytoplasmatische Veränderungen in den Nierentubuli, Pigmente in Knochenmark und Milz sowie in der Leber mehrkernige Riesenzellen auf. Der NOAEL lag bei der niedrigsten Dosis von 113/109 mg/kg KG und Tag (NTP 2020). 

#### 5.2.3 Dermale Aufnahme

Die Untersuchungen sind in der Tabelle 4 dargestellt.

Es wurden jeweils zwei Zwei-Wochen-Studien mit F344-Ratten und B6C3F1-Mäusen durchgeführt, bei denen die Applikation von Benzophenon-3 in Aceton bzw. in Lotion erfolgte. Bei Ratten betrugen die Dosierungen 0; 1,25; 2,5; 5; 10 oder 20 mg/Tier (ca. 6,25/8,33; 13/16,7; 25/33; 50/66; 100/133 mg/kg KG und Tag für ♂/♀ bei 200/150 g KG), bei Mäusen 0; 0,5; 1; 2; 4 und 8 mg/Tier (ca. 25, 50, 100, 200, 400 mg/kg KG und Tag mit 20 g KG für ♂ und ♀). Als einzige Befunde traten bei Ratten und Mäusen leicht erhöhtes Lebergewicht (Ratten: 7–15 %, Mäuse: 10–23 %) oder Nierengewicht (Ratten: max. 5 %, Mäuse: max. 7 %) ohne histopathologisches Korrelat auf, wobei diese Organgewichtserhöhungen bei Applikation in Lotion leicht stärker waren als bei Aceton als Vehikel (French 1992).

Eine **13-Wochen-Studie** mit Applikation in Aceton erfolgte bei F344-Ratten mit 0; 12,5; 25; 50; 100 oder 200 mg/kg KG und Tag, bei B6C3F1-Mäusen mit 0; 22,75; 45,5; 91; 182 oder 364 mg/kg KG und Tag. Bei Ratten wurde bei 25 mg/kg KG und Tag nicht dosisabhängig erhöhtes Nierengewicht beobachtet und ab 50 mg/kg KG und Tag bei weiblichen Tieren eine erhöhte Thrombozytenzahl und bei der höchsten Dosis Lymphozytose. Bei den Mäusen nahm die Spermienzahl in den Nebenhoden ab 22,75 mg/kg KG und Tag dosisabhängig ab und ebenfalls bei männlichen Mäusen waren Nieren- und Herzgewicht nicht dosisabhängig erhöht (French 1992).

Eine weitere dermale 13-Wochen-Studie an männlichen B6C3F1-Mäusen mit bis zu 400 mg Benzophenon-3/kg KG und Tag zeigte keine Effekte auf Körpergewicht, Spermienparameter und die Histopathologie der Testes (Daston et al. 1993). Weitere Organe wurden nicht untersucht.

**Fazit**: In einer dermalen 13-Wochen-Studie trat bei B6C3F1-Mäusen ab der niedrigsten Dosis von 22 mg/kg KG und Tag eine verminderte Spermienzahl in den Nebenhoden auf. Ein NOAEL wurde somit nicht erhalten. Bei F344-Ratten lag der NOAEL bei 12,5 mg/kg KG und Tag, ab 25 mg/kg KG und Tag kam es zu einem erhöhten relativen Nierengewicht von ca. 10 % ohne histopathologisches Korrelat (French 1992).

**Tab. 4:** Wirkung von Benzophenon-3 nach wiederholter dermaler Verabreichung

**Stamm, Anzahl pro Gruppe**	**Exposition**	**Befunde**	**Literatur**
**Ratte**, F344/N, 5 ♂, 5 ♀	**2 Wochen**, 0; 1,25; 2,5; 5; 10; 20 mg/Tier (ca. 6,25/8,33; 13/16,7; 25/33; 50/66; 100/133 mg/kg KG u. d bei 200/150 g KG für ♂ u. ♀) **in Aceton**, 5 d/Wo	**ab 33 mg/kg KG**: ♀: abs. (15 %) u. rel. (12 %) Lebergewicht ↑; **66** **mg/kg KG**: ♀: rel. Nierengewicht 5 % ↑	French 1992
**Ratte**, F344/N, 5 ♂, 5 ♀	**2 Wochen**, 0; 1,25; 2,5; 5; 10; 20 mg/Tier (ca. 6,25/8,33; 13/16,7; 25/33; 50/66; 100/133 mg/kg KG u. d bei 200/150 g KG für ♂ / ♀) **in Lotion**, 5 d/Wo	**ab 66 mg/kg KG**: ♀: abs. (9 %) u. rel. (8 %) Lebergewicht ↑; **25 u. 100 mg/kg KG**: ♂: abs. (13 % u. 13 %) u. rel. (8 % u. 7 %) Lebergewicht ↑	French 1992
**Ratte**, F344/N, 10 ♂, 10 ♀	**13 Wochen**, 0; 12,5; 25; 50; 100; 200 mg/kg KG u. d **in Aceton**, 5 d/Wo	**12,5** **mg/kg KG**: **NOAEL**; **bei 25** **mg/kg KG**: ♂: rel. Nierengewicht 10 % ↑; **ab 25** **mg/kg KG**: ♀: rel. Nierengewicht 8–19 % ↑ (nicht dosisabhängig); **ab 50** **mg/kg KG**: ♀: Thrombozyten ↑; **200 mg/kg KG**: ♀: Lymphozytose	French 1992
**Maus**, B6C3F1, 5 ♂, 5 ♀	**2 Wochen**, 0; 0,5; 1; 2; 4; 8 mg/Tier (0, ca. 25, 50, 100, 200, 400 mg/kg KG u. d bei 20 g KG für ♂ u. ♀) **in Aceton**, 5 d/Wo	**ab 200** **mg/kg KG**: abs. u. rel. (♂ 11 % u. 10 %, ♀ 24 % u. 14 %) Lebergewicht ↑, ♂: rel. Nierengewicht 7 % ↑	French 1992
**Maus**, B6C3F1, 5 ♂, 5 ♀	**2 Wochen**, 0; 0,5; 1; 2; 4; 8 mg/Tier (0, ca. 25, 50, 100, 200, 400 mg/kg KG u. d bei 20 g KG für ♂ u. ♀) **in Lotion**, 5 d/Wo	**ab 200** **mg/kg KG**: ♂: abs. (20 %) u. rel. (14 %) Lebergewicht ↑; **400** **mg/kg KG**: ♀: abs. (22 %) u. rel. (17 %) Lebergewicht ↑	French 1992
**Maus**, B6C3F1, 10 ♂, 10 ♀	**13 Wochen**, 0; 22,75; 45,5; 91; 182; 364 mg/kg KG u. d **in Aceton**, 5 d/Wo	♂: rel. Nierengewicht u. Herzgewicht nicht dosisabhängig ↑, **22,75/91/364** **mg/kg KG**: Spermienzahl in Nebenhoden dosisabhängig ↓ (dieser Parameter nur bei diesen Dosierungen untersucht)	French 1992

abs.: absolut; d: Tag; rel.: relativ; u: und; Wo: Woche

### 5.3 Wirkung auf Haut und Schleimhäute

#### 5.3.1 Haut

Benzophenon-3 (500 mg auf mit destilliertem Wasser angefeuchtete Pflaster) wirkte in einer Untersuchung nach OECD-Prüfrichtlinie 404 aus dem Jahr 1990 bei semiokklusiver Auftragung vier Stunden lang auf die geschorene Rückenhaut nicht reizend bei drei weiblichen Weiße-Neuseeländer-Kaninchen. Es zeigte sich keine Hautreaktion bei den Ablesezeitpunkten zwischen 24 und 72 Stunden nach Substanzgabe (ECHA 2013).

Mit Wasser angefeuchtetes Benzophenon-3 (500 mg) wirkte auch nach 24-stündiger okklusiver Applikation an der intakten oder abradierten Haut von je drei männlichen und je drei weiblichen Weiße-Neuseeländer-Kaninchen nicht reizend (ECHA 2013).

Vier bis 100 % Benzophenon-3 war nicht reizend an der intakten oder abradierten Haut von Albino-Kaninchen (k. w. A.; French 1992).

In einem Test nach OECD-Prüfrichtlinie 439 an humaner rekonstruierter Epidermis (EpiDerm^TM^) wirkte unverdünntes Benzophenon-3 (30 µl) nach 60-minütiger Applikation nicht reizend. Die durch die Resorption des aus MTT (3-(4,5-Dimethylthiazol-2-yl)-2,5-diphenyltetrazoliumbromid) gebildeten Farbstoffs Formazan bestimmte durchschnittliche Zellviabilität betrug 97,8 % im Vergleich zur Negativkontrolle. Die Positivkontrolle Natriumdodecylsulfat (SDS) zeigte ein funktionierendes Testsystem an (Han et al. 2020).

##### Phototoxizität

Um Benzophenon-3 auf phototoxische Wirkung zu untersuchen, wurden auf die rasierte Haut von Albino-Kaninchen 24 mg Benzophenon-3 aufgetragen und diese Hautstelle fünfmal pro Woche, zwei Wochen lang mit UV-Licht bestrahlt. Es entstanden eine leichte Rötung und leichte Ödeme und die Hautstelle schuppte sich etwas. Es trat keine phototoxische Wirkung auf (k. w. A.; French 1992).

In einer Validierungsstudie führten neun Testlabore den 3T3-Neutralrot-Phototoxizitätstest mit Benzophenon-3 durch. Dabei wurden BALB/c-3T3-Mausfibroblasten 60 Minuten lang mit verschiedenen Konzentrationen Benzophenon-3 (maximale Konzentrationen je nach Labor 100 bis 10 000 µg/ml) behandelt und anschließend erfolgte eine 50-minütige Bestrahlung mit 5 J/cm^2^ UVA (Kontrolle im Dunkeln ohne Bestrahlung). Nach 24 Stunden wurde die Aufnahme des Vitalstoffes Neutralrot (NRU) gemessen und die jeweiligen IC_50_-Werte als die Konzentrationen der Testsubstanz definiert, die eine 50%ige Verringerung von NRU im Vergleich zu den unbehandelten Kontrollkulturen verursachen. Anschließend wurde der Photoirritationsfaktor (PIF) berechnet, indem zwei gleich wirksame zytotoxische Konzentrationen (IC_50_) der Testchemikalie verglichen wurden, die in Abwesenheit (–UV) und in Gegenwart (+UV) einer nicht zytotoxischen Bestrahlung erhalten wurden. Substanzen mit einem PIF ≥ 5 galten als phototoxisch. Zusätzlich wurde der mittlere Photoeffekt (MPE) auf der Grundlage eines Vergleichs der vollständigen Konzentrations-Wirkungs-Kurven berechnet. Substanzen mit einem MPE ≥ 0,1 wurden als phototoxisch bewertet. Benzophenon-3 lag bei acht von neun Laboratorien unter den jeweiligen Grenzwertkriterien für Phototoxizität. Bei dem neunten Studienlabor lag der PIF-Wert über 2,7 und der MPE-Wert bei 0,195, was von den Autoren als zufällig angesehen wurde (Spielmann et al. 1998). Somit war Benzophenon-3 im Phototoxizitätstest unter Versuchsbedingungen, die inzwischen in der OECD-Prüfrichtlinie 432 festgelegt worden sind, negativ.

Anhand der OECD-Prüfrichtlinie 432 wurde Benzophenon-3 auf sein phototoxisches Potenzial untersucht. Dies erfolgte in vitro an 3T3-Fibroblastenkulturen mittels des NRU-Phototoxizitätstests. Die Zellen wurden nach Exposition gegen Benzophenon-3 einer Bestrahlung ausgesetzt bzw. die Kontrollen nicht. In dieser Untersuchung war Benzophenon-3 negativ: der PIF betrug 1 und lag damit unterhalb der Grenze von 2 für ein positives Ergebnis. Der sekundäre Parameter MPE soll für ein negatives Ergebnis kleiner 0,1 sein und lag in einem von zwei Durchläufen genau bei 0,1 (Benevenuto et al. 2015). Da der MPE nur zur Bewertung herangezogen wird, wenn keine IC_50_ und damit kein PIF bestimmbar ist, was bei Benzophenon-3 nicht der Fall war, wird Benzophenon-3 als negativ gewertet.

Eine weitere Untersuchung prüfte Phototoxizität in verschiedenen Zelllinien, auch gemäß OECD-Prüfrichtlinie 432, die sich jedoch nur auf die 3T3-Zelllinie bezieht. Dabei war die BALB/c-3T3-Fibroblastenzelllinie der Maus sensitiv für Bestrahlung mit UVA- und UVB-Licht, während die humane Fibroblastenzelllinie HS68 vor allem empfindlich für UVA und die humane Keratinozytenzelllinie HaCaT für UVB war. Benzophenon-3 war in den drei Zelllinien nicht phototoxisch bei UVB-Bestrahlung und bei UVA-Bestrahlung nur in einem von zwei Durchläufen mit HaCaT positiv, alle Durchläufe mit den anderen Zelllinien waren negativ (Xiong et al. 2019). Da die nach OECD-Prüfrichtlinie relevante Zelllinie die 3T3-Fibroblastenzelllinie ist und diese in allen Untersuchungen negativ war, ist Benzophenon-3 auch in dieser Untersuchung nicht phototoxisch.

#### 5.3.2 Auge

Benzophenon-3-Pulver (0,1 ml, 32 mg) wirkte in einer Untersuchung nach OECD-Prüfrichtlinie 405 aus dem Jahr 1990 sehr leicht reizend am Auge von drei weiblichen Weiße-Neuseeländer-Kaninchen, wobei die Stärke der Wirkung keiner Einstufung nach der CLP-Verordnung der EU bedarf. Die individuellen Reizindizes nach 24, 48 und 72 Stunden betrugen für Bindehautrötung 1; 1; 0,67 (von maximal 3). Schwellungen wurde ausschließlich in der ersten Stunde nach Substanzgabe beobachtet, Cornea und Iris waren nicht betroffen. Alle Befunde waren bis zum Ende der siebentägigen Nachbeobachtungszeit reversibel (ECHA 2013).

In einer weiteren Studie aus dem Jahr 1969 wurden 0,1 g Benzophenon-3-Pulver in den Konjunktivalsack von je drei männlichen und drei weiblichen Weiße-Neuseeländer-Kaninchen eingebracht und bis zu sieben Tage nach der Applikation keine Anzeichen einer reizenden Wirkung beobachtet (ECHA 2013).

#### 5.3.3 Fazit

Pulverförmiges, angefeuchtetes Benzophenon-3 wirkt nicht reizend an der Haut von Kaninchen und an rekonstituierter Humanhaut in vitro. Benzophenon-3 war in drei In-vitro-Untersuchungen auf Phototoxizität nach OECD-Prüfrichtlinie 432 negativ, daher wären bei Pharmazeutika nach ICH S10 auch keine In-vivo-Untersuchungen erforderlich. Am Kaninchenauge wirkt die Substanz als Pulver nicht bis maximal sehr leicht reizend.

### 5.4 Allergene Wirkung

#### 5.4.1 Kontaktsensibilisierende und photokontaktsensibilisierende Wirkung

##### 5.4.1.1 Tierexperimentelle Untersuchungen

Benzophenon-3 wurde in einem Local Lymph Node Assay nach OECD-Prüfrichtlinie 429 jeweils an vier weiblichen CBA-Mäusen pro Dosisgruppe getestet. Mit den Konzentrationen 12,5; 25 oder 50 % in Dimethylformamid wurden Stimulationsindices von 1,64; 1,33 und 1,61 beobachtet, ab einem Stimulationsindex von 3 wird das Ergebnis als positiv gewertet. Das Ergebnis ist damit negativ (ECHA 2013).

Des Weiteren liegen Ergebnisse aus zwei Maximierungstests vor.

Im ersten Test (ähnlich zur späteren OECD-Prüfrichtlinie 406) an Hartley-White-Meerschweinchen erfolgte die intradermale Induktion mit 5 % Benzophenon-3 in Maiskeimöl oder 5 % Benzophenon-3 in 50 % wässrigem Freundschem Adjuvans. Eine Woche später wurde auf dieselbe, zuvor mit 10 % SDS behandelte Hautstelle eine 10%ige Testzubereitung von Benzophenon-3 in Vaseline appliziert. Die Provokation erfolgte mit der 2,5%igen Testzubereitung in Vaseline auf einer zuvor unbehandelten Hautstelle. Nach 48 Stunden zeigten 18 der 20 Tiere keine Reaktion, bei zwei Tieren wurde ein leichtes Erythem beobachtet. Nach 72 Stunden zeigten 17 von 20 Tieren keine Reaktion und drei Tiere ein leichtes Erythem (jedoch andere Tiere als zuvor). Von 30 Kontrolltieren zeigte ebenfalls keines eine Reaktion (sekundär zitiert in SCCP 2006). In der Testbeschreibung ist die Anzahl der getesteten Tiere widersprüchlich, vermutlich wurden 20 Tiere getestet. 

In einem weiteren Maximierungstest an 20 Pirbright-White-Meerschweinchen erfolgte die intradermale Induktion mit 5 % Benzophenon-3 in Erdnussöl. Für die epidermale Induktion wurde eine 30%ige Testzubereitung in Vaseline eingesetzt. Auf die Provokation mit einer 10%igen Testzubereitung in Vaseline reagierte nach 24 Stunden keines und nach 48 Stunden eins von 20 Tieren (ECHA 2013). Die Konzentrationen sind nicht gemäß Prüfrichtlinie gewählt: In der Vorstudie wurden mit einer Testzubereitung von 30 % keine Anzeichen erythematöser Reaktionen beobachtet. Die Autoren wählten für die Provokation trotzdem die 10%ige Testzubereitung, da nach deren Aussage eine höhere Konzentration zu unspezifischen Reaktionen bei mit Adjuvans behandelten Tieren führen könnte. Eine Vorbehandlung mit SDS wurde nicht durchgeführt. 

Ein dritter Maximierungstest, der jedoch nur sekundär zitiert und unvollständig dokumentiert ist, lieferte ein negatives Ergebnis (French 1992).

Zu Benzophenon-3 ist eine Untersuchung zur photokontaktsensibilisierenden Wirkung nur als Sekundärzitat verfügbar. Bei der Behandlung von Albino-Kaninchen mit einem Sonnenschutzmittel, das 6 % Benzophenon-3 enthielt, wurde keine Photosensibilisierung beobachtet. Details zur Durchführung fehlen (SCCP 2008).

##### 5.4.1.2 Ergebnisse aus nicht-tierbasierten Alternativverfahren zur Untersuchung der hautsensibilisierenden Wirkung 

Zur Ableitung hautsensibilisierender Eigenschaften einer Chemikalie werden gemäß der OECD-Richtlinie 497 die Ergebnisse aus Testverfahren miteinander verknüpft, die Schlüsselereignisse (Key Events, KE) des Adverse Outcome Pathway (AOP) prüfen. Die Berücksichtigung mehrerer Testverfahren und deren Verknüpfung bei der Ableitung des sensibilisierenden Potenzials einer Chemikalie beruht auf der Annahme, dass ein Einzeltestverfahren die komplexe Abfolge bei der Entwicklung einer Sensibilisierung nicht abbilden kann.

Zur Ableitung eines Gesamtergebnisses unter Anwendung der Integrated Testing Strategy Version 2 (ITSv2) gemäß der OECD-Richtlinie 497 können Ergebnisse aus dem Direkten Peptidreaktivitätstest (DPRA, KE1), dem Human Cell Line Activation Test (h-CLAT, KE3) sowie Ergebnisse aus Untersuchungen aus computergestützten Methoden (OECD QSAR Toolbox v 4.7; OECD 2024) berücksichtigt werden.

Das Ergebnis für Benzophenon-3 im h-CLAT in Analogie zur späteren OECD-Prüfrichtlinie 442 E und Auswertung nach heutigen Kriterien ist positiv (Hoya et al. 2009).

Für Benzophenon-3 liegt kein Ergebnis im DPRA vor. Daher basiert die Integration auf den Daten des h-CLAT (bewertet mit 2/3 Punkten) und der OECD QSAR Toolbox (0/1 Punkten). Insgesamt ergibt sich daraus eine Gesamtpunktzahl von 2/4 Punkten, was gemäß OECD-Richtlinie 497 zu einem positiven Gesamtergebnis mit Klassifikation in GHS-Kategorie 1B (moderates Allergen) führt.

##### 5.4.1.3 Ergebnisse aus nicht-tierbasierten Methoden in Entwicklung zur Untersuchung der photosensibilisierenden Wirkung 

Der molare Extinktionskoeffizient von Benzophenon-3 ist 9700 M^–1^cm^–1^ (Lovell und Jones 2000). Damit hat der Stoff grundsätzlich ein photoaktives Potenzial (OECD-Prüfrichtlinie 432). Der Phototoxizitätstest nach OECD-Prüfrichtlinie 432 an 3T3-Zellen sowie an zwei weiteren Zelllinien (HS68 (auch Fibroblasten) und HaCaT) verlief jedoch negativ für Benzophenon-3 (Xiong et al. 2019; Abschnitt 5.3.1).

Das Molekül wurde im Rahmen der Entwicklung verschiedener Photosensibilisierungstests miteingeschlossen. In Methoden, die die Peptid- und Proteinbindung von Substanzen unter UVA-Belichtung untersuchen, wurde Benzophenon-3 negativ getestet (Lovell und Jones 2000).

Ein Photo-Human Cell Line Activation Test (Photo-hCLAT) mit Benzophenon-3 lieferte ein positives Ergebnis (Hoya et al. 2009).

#### 5.4.2 Atemwegssensibilisierende Wirkung

Hierzu liegen keine Daten vor.

### 5.5 Reproduktionstoxizität

#### 5.5.1 Fertilität

In einer modifizierten Ein-Generationenstudie erhielten je 25 Sprague-Dawley-Ratten (Hsd:Sprague Dawley^®^ SD^®^) pro Dosis und Geschlecht 0, 3000, 10 000 oder 30 000 mg Benzophenon-3/kg Futter ab dem 6. Trächtigkeitstag. Dies entsprach während der Trächtigkeit (6. bis 21. Tag) 0, 205, 697 und 2644 mg Benzophenon-3/kg KG und Tag und während der Laktation (1. bis 13. Tag) 0, 484, 1591 und 5120 mg Benzophenon-3/kg KG und Tag. Die Substanzaufnahme der F1-Tiere nach dem Entwöhnen am 28. Lebenstag bis zum 91. Lebenstag betrug 0, 267, 948 und 3003 mg/kg KG und Tag (männliche Tiere) und 0, 287, 983 und 3493 mg/kg KG und Tag (weibliche Tiere). Die erwachsenen weiblichen F1-Tiere nahmen 0, 240, 825 und 2760 mg Benzophenon-3/kg KG und Tag (0. bis 21. Trächtigkeitstag) und 0, 426, 1621 und 5944 mg/kg KG und Tag (1. bis 13. Laktationstag) auf. Die Untersuchung erfolgte an einer pränatalen Kohorte und einer Kohorte zur Untersuchung der Fertilitätsparameter. Als Positivkontrolle wurde einer Gruppe 0,05 mg Ethinylöstradiol/kg Futter verabreicht. Bis zur höchsten Dosis wurden keine Benzophen-3-induzierten Effekte auf Paarungs- und Trächtigkeitsindices festgestellt. In der F1-Kohorte wurde bei der höchsten Dosis eine statistisch signifikante Erniedrigung der durchschnittlichen Anzahl an Corpora lutea/Muttertier (14,89 ± 0,87; Kontrolle: 18,56 ± 0,77), F2-Implantationen/Muttertier (12,94 ± 0,88; Kontrolle: 15,61 ± 0,65) und der Anzahl lebender Feten am 21. Trächtigkeitstag/Wurf im Vergleich zur Kontrolle (12,0 ± 0,4; Kontrolle: 13,6 ± 0,5) beobachtet. In der F0- und der F1-Generation war während der Trächtigkeit die Körpergewichtszunahme ab der mittleren Dosis statistisch signifikant erniedrigt, in der F0-Generation war der Futterverbrauch bei der höchsten Dosis statistisch signifikant erhöht, jedoch nicht in der F1-Generation. Benzophenon-3 führte nicht zu Veränderungen der Östrogen- und Androgen-vermittelten Entwicklungsmarker. Bei den adulten Tieren wurden bei der Nekropsie auch keine Veränderungen gesehen, die Störungen der Östrogen- und Androgen-Rezeptor-vermittelten Entwicklung anzeigen. Die Positivkontrolle ergab die entsprechenden östrogen-abhängigen Effekte. Basierend auf der geringfügig erniedrigten Wurfgröße in der F2-Generation in den beiden Kohorten (Pränatal- und Fertilitätskohorte) wurde die Reproduktionstoxizität von Benzophenon-3 an Sprague-Dawley-Ratten als nicht eindeutig bewertet. Die erniedrigte Wurfgröße könnte ein direkter Effekt (eine Abnahme der ovulierten Eizellen, gezeigt in der pränatalen Kohorte) oder ein indirekter Stress-induzierter Effekt (geringeres Körpergewicht) sein. Die Spermienzahlen blieben ohne Veränderungen durch die Benzophenon-3-Exposition (NTP 2022 a). Daraus leitet sich ein NOAEL für Fertilität von 10 000 mg/kg Futter (F1-Generation, männliche bzw. weibliche Tiere: 948 bzw. 983 mg/kg KG und Tag) ab. Bei dieser Dosis waren Körpergewicht bzw. Körpergewichtszunahme während der Trächtigkeit erniedrigt, sodass der NOAEL für Maternaltoxizität bei der niedrigsten Dosis (3000 mg/kg Futter, F1-Generation, 287 mg/kg KG und Tag) liegt.

Es wurde von NTP eine Reproduktionsstudie mit kontinuierlicher Verpaarung (RACB, Reproductive Assessment by Continuous Breeding) an VAF Crl: Swiss CD-1 (ICR)BR-Mäusen in den Dosierungen 0, 12 500, 25 000 oder 50 000 mg Benzophenon-3 (Reinheit > 99 %)/kg Futter (männliche Tiere: 0, 1800, 3800, 8600 mg/kg KG und Tag, weibliche Tiere: 0, 1900, 4100, 9500 mg/kg KG und Tag) durchgeführt. Die Tiere (20 Tiere/Dosisgruppe und Geschlecht, 40 Tiere/Kontrollgruppe und Geschlecht) wurden kontinuierlich sieben Tage vor dem Zusammensetzen sowie während einer 98-tägigen Kohabitationszeit behandelt. Ab der mittleren Dosis war der Futterverbrauch statistisch signifikant erhöht (errechnet pro Käfig), jedoch das Körpergewicht in der F0-Generation nach zwei und drei Wochen erniedrigt. Es zeigte sich kein Einfluss auf die Fertilität der F0-Tiere, aber die Zahl an lebenden Nachkommen pro Wurf war statistisch signifikant ab der mittleren Dosis verringert. Dies ging mit verringerten Körpergewichten der Elterntiere einher. Es traten keine veränderten Spermienparameter (Spermienbeweglichkeit, Anzahl, Morphologie) oder veränderter Östruszyklus auf, die kumulativen Tage bis zum Werfen waren für den zweiten und dritten Wurf in der höchsten Dosisgruppe jedoch erhöht. Bei den F1-Tieren war das Körpergewicht der Jungtiere statistisch signifikant verringert, ansonsten zeigte sich nur ein minimaler Effekt auf die Fertilität im Erwachsenenalter. Benzophenon-3 war systemisch toxisch, hatte aber bei den untersuchten Dosierungen nur minimale Effekte auf die Fertilität oder Reproduktion (NTP 1990). Der NOAEL für Maternaltoxizität und Fertilität lag bei der niedrigsten Dosis von 1900 mg/kg KG und Tag.

In einer Fertilitätsstudie mit kontinuierlicher Verpaarung wurden zehn trächtige C57BL/6-Mäuse dermal (offen auf dem rasierten Rücken, neun Kontrolltiere) gegen 0 oder 50 mg Benzophenon-3/kg KG und Tag vom 9. Gestationstag bis zum 21. Postnataltag exponiert (Vehikel Olivenöl). Die kontinuierliche Verpaarung der männlichen Nachkommen von behandelten Muttertieren mit weiblichen Nachkommen der unbehandelten Muttertiere und umgekehrt über sechs Monate führte bei den In-utero-behandelten weiblichen Nachkommen zu einer reduzierten Anzahl an Würfen, was eine reduzierte weibliche Fertilität anzeigt. Die weiblichen Nachkommen behandelter Muttertiere wiesen am 77. Postnataltag eine erniedrigte Zahl von Oozyten und Primordialfollikeln auf, die vermutlich für die reduzierte Fertilität verantwortlich ist. Das Körpergewicht der Nachkommen war transient im Vergleich zu den Kontrollen erniedrigt (männliche Nachkommen: 3. bis 18. Postnataltag, weibliche Nachkommen: 15. und 18. Postnataltag). Es ergaben sich keine substanzbedingten Veränderungen beim Körpergewicht der Muttertiere, der Gestationslänge, der Anzahl der Nachkommen/Muttertier, dem Beginn der Pubertät sowie dem Geschlechterverhältnis. Auch der Östruszyklus der weiblichen Nachkommen und die Spermienzahl der männlichen Nachkommen sowie die Hormonkonzentrationen (Östradiol, Progesteron, Testosteron) im Serum blieben durch die Benzophenon-3-Behandlung unbeeinflusst (Galliani et al. 2024).

Die Exposition von adulten Tieren gegen Benzophenon-3 führte zu einer veränderten Spermienkonzentration und Vaginalzytologie. Männliche F344/N-Ratten, die 90 Tage lang 50 000 mg Benzophenon-3/kg Futter (3656 mg/kg KG und Tag) erhielten, wogen 30 % weniger als die Kontrolltiere, ihr rechter Nebenhoden 17 % weniger und der rechte kaudale Nebenhoden 22 % weniger, und die Spermienkonzentration war um 27 % verringert. Bei weiblichen F344/N-Ratten war die Länge des Östruszyklus bei 50 000 mg Benzophenon-3/kg Futter (3261 mg/kg KG und Tag) um einen Tag verlängert, während das Körpergewicht um 12 % reduziert war. Geringere Dosierungen führten nicht zu diesen Effekten (siehe Abschnitt 5.2.2; French 1992). Bei männlichen B6C3F1-Mäusen traten nach 90-tägiger Gabe bei 50 000 mg/kg Futter (13 937 mg/kg KG und Tag) ein um 16 % vermindertes Körpergewicht und um 27 % verminderte Spermienkonzentrationen auf. Weibliche B6C3F1-Mäuse dieser Dosisgruppe (18 539 mg/kg KG und Tag) hatten einen um mindestens 0,5 Tage verlängerten Östruszyklus bei einem um 13 % niedrigeren Körpergewicht. Geringere Dosierungen führten nicht zu diesen Effekten (siehe Abschnitt 5.2.2; French 1992).

In der dermalen 90-Tage-Studie an männlichen und weiblichen F344-Ratten kam es bis zur höchsten Dosis von 200 mg/kg KG und Tag nicht zu Veränderungen an den Reproduktionsorganen einschließlich Spermien und Östruszyklus (French 1992).

In der dermalen 90-Tage-Studie (5 Tage/Woche) an B6C3F1-Mäusen trat ab der niedrigsten Dosis von 22,75 mg/kg KG und Tag eine dosisabhängige erniedrigte Spermienkonzentration in den Nebenhoden auf (untersuchte Dosierungen: 22,75; 91; 364 mg/kg KG und Tag). Effekte auf das Körpergewicht und die weiblichen Reproduktionsorgane, einschließlich Östruszyklus, traten bis zur höchsten Dosis von 364 mg/kg KG und Tag nicht auf (French 1992).

In einer dermalen 13-Wochen-Studie an männlichen B6C3F1-Mäusen mit bis zu 400 mg Benzophenon-3/kg KG und Tag zeigten sich keine Effekte auf Körpergewicht, Spermienparameter und die Histopathologie der Testes (Daston et al. 1993). Die Autoren erklärten die Diskrepanz zur Studie von French (1992) mit deren hoher Spermatozoenkonzentration, die über den historischen Kontrolldaten lag. In ihrer eigenen Studie lag die Spermatozoenkonzentration allerdings klar unter den historischen Kontrolldaten.

#### 5.5.2 Entwicklungstoxizität

In Tabelle 5 werden die Studien zur Entwicklungstoxizität von Benzophenon-3 aufgeführt.

**Tab. 5:** Entwicklungstoxizitätsstudien nach Verabreichung von Benzophenon-3

**Spezies, Stamm, Anzahl pro Gruppe**	**Exposition**	**Befunde**	**Literatur**
Pränatale Exposition			
Ratte, Wistar (Crl:WI(Han)), 25 ♀	OECD TG 414, GD 6–19, 0, 40, 200, 1000 mg/kg KG u. d, Gavage, Reinheit 99,8 %, Vehikel: Maiskeimöl, Untersuchung GD 20	**200 mg/kg KG**: **NOAEL** Entwicklungs- u. Maternaltoxizität; **ab 200 mg/kg KG**: Muttertiere: transienter Speichelfluss sofort nach der Dosierung (GD 12–19: 3/25 Tiere, nicht advers); **bei 1000 mg/kg KG**: Muttertiere: transienter Speichelfluss sofort nach der Dosierung (GD 7–19: alle Tiere), mit Urin beflecktes Fell (GD 7–20: 16/25 Tiere), rötlich verfärbter Urin (GD 13–20: 10/25, wahrscheinlich Substanz od. Metaboliten), Futteraufnahme ↓ (GD 6–10: –30,3 %, GD 6–19: –3,5 %), KG-Zunahme ↓ (GD 6–8: –59,7 %, GD 6–19: –7,4 %), korrigierte KG-Zunahme ↓ (terminales KG am GD 20 abzgl. Gew. des nicht geöffneten Uterus abzgl. KG am GD 6, –20,8 %), Feten: skelettale Variationen auf Feten- u. Wurfbasis ↑ (nicht vollständige Ossifikationen von Schädelknochen u. der Halswirbelsäule, zusätzliche 14. Rippen, Gesamtzahl der Variationen), keine Teratogenität	BASF AG Experimental Toxicology and Ecology 2005; SCCP 2008; SCCS 2021
Maus, ICR, 15 ♀	GD 0–6, 0; 0,1; 10; 1000 mg/kg KG u. d, Gavage, Reinheit 98 %, Vehikel: Maiskeimöl, Untersuchung GD 12	**ab 0,1 mg/kg KG**: Muttertiere: Plazenta: beginnende Thrombose u. Gewebsnekrose mit einer verstärkten Thrombozytenaggregation (k. A. von Inzidenzen u. Schweregraden); **1000 mg/kg KG**: Muttertiere: fetaler Verlust ↑, keine Effekte auf KG, rel. Organgewicht von Leber, Gehirn, Uterus, Plazenta, Feten: kein Effekt auf KG	Han et al. 2022
Maus, ♀ C57BL/6J verpaart mit ♂ BALB/c, 4–6 ♀/Zeitpunkt (insgesamt 19/Dosis)	GD 0–6, 0, 50 mg/kg KG u. d (applizierte Dosis entspricht der errechneten Dosis beim Menschen nach Ganzkörperapplikation einer Sonnencreme^a)^), dermal (offen auf den rasierten Rücken), Einzeltierhaltung im Käfig während Gestation zur Verhinderung der oralen Aufnahme durch andere Tiere; SCCS 2021), Reinheit 98 % (SCCS 2021), Vehikel: Olivenöl, Untersuchung GD 5, 10, 14	**50 mg/kg KG**: Muttertiere: Uterusarterie transiente Veränderung von hämodynamischen Parametern (nur GD 10, k. A. zur KG-Entwicklung), kein Effekt auf die Implantationen, Serum: 19,3–24,8 ng/ml (4 Proben, GD 6; am GD 14: 3 Proben: in 2 nicht nachweisbar u. 16,8 ng/ml), Feten: intrauterine Wachstumsrestriktion (Ultraschalluntersuchung GD 5, 8, 10, 12, 14), KG ↓ (GD 14, Plazentagewicht nicht verändert), feto-plazentaler Index ↓, Geschlechterverhältnis verändert: Prozentsatz ♀ ↑ (ca. 84 %, Kontrolle: ca. 64 % aus Abbildung), Benzophenon-3 im Serum: 22,4 ng/ml (4 h nach Applikation am GD 6), 16,8 ng/ml (8 d nach Applikation am GD 6)^b)^; 2. Trächtigkeit (ohne weitere Exposition): Muttertiere: Plazentagewicht ↓ (fetales KG am GD 14 nicht verändert), Benzophenon-3 im Serum nicht nachweisbar (Nachweisgrenze: 0,7 ng/ml), Feten: feto-plazentaler Index ↓, Prozentsatz ♀ ↑	Santamaria et al. 2020
Maus, ♀ C57BL/6 verpaart mit ♂ BALB/c, 7 ♀/Zeitpunkt	GD 6 bis maximal GD 10, 0, 50 mg/kg KG u. d, dermal (offen auf den rasierten Rücken), 5–10 min nach Applikation Tiere einzeln im Käfig zur Verhinderung der oralen Aufnahme, bis Öl visuell völlig resorbiert, Reinheit 98 % wie bei Santamaria et al. 2020, Vehikel: Olivenöl, Untersuchung GD 10, 12, 14	**50 mg/kg KG**: Muttertiere: kein Effekt auf die Implantationen, Plazentamorphologie u. -effizienz verändert, hämodynamische Parameter u. Umbau der Spiralarterie im Uterus nicht verändert, Plazenta (GD 14): Genexpression bei ♀ Feten: Pgr, Icam1, Gzmb ↓, keine Veränderung bei Esr1, Esr2, Genexpression bei ♂ Feten: keine Veränderung Esr1, Esr2, Pgr, Icam1, Gzmb; Feten: intrauterine Wachstumsrestriktion (Ultraschalluntersuchung GD 5, 8, 10, 12, 14), Plazentagewicht nicht verändert, keine Effekte bei den Muttertieren auf KG u. KG-Zunahme, Uterus u. Dezidua: Prozentsatz u. abs. Zahlen von Immunzellpopulationen (T-, B-, NK-, Mastzellen, Makrophagen) nicht verändert, k. A. zum Geschlechterverhältnis, keine Messung der Benzophenon-3-Konzentrationen im Serum	Fischer et al. 2024 (gleiche Arbeitsgruppe wie Santamaria et al. 2020)
Prä- u. postnatale Exposition			
Ratte, Sprague Dawley, 7–8 ♀	GD 6–PND 23, 0, 1000, 3000, 10 000, 25 000, 50 000 mg/kg Futter (0, 68, 207, 671, 1798, 3448 mg/kg KG u. d), Reinheit k. A. (Ivy Fine Chemicals), Untersuchung GD 10, 15, 20, PND 23	**207 mg/kg KG**: **NOAEL** Maternaltoxizität; **ab 207 mg/kg KG**: Nachkommen: ♂ Testes: Spermatozytenzahl/Hodenkanälchen ↓ (PND 23); **ab 671 mg/kg KG**: Muttertiere: Leber: abs. u. rel. Gewicht ↑ (mindestens 51 %); **ab 1798 mg/kg KG**: Muttertiere: KG ↓ (GD 10, 13), Serum: ALT, ALP ↑, Nachkommen: KG ↓ (PND 23), ♀ Verzögerung der follikulären Entwicklung, Anzahl der antralen Follikel ↓ (PND 23); **bei 3448 mg/kg KG**: Muttertiere: KG ↓ (GD 10, 13, 20), Serumkonzentrationen von BP-3 u. DHB dosis- u. zeitabhängig ↑ (BP-3 am GD 20 in aufsteigender Dosis, angefangen mit Kontrolle: < NG (0,005 µg/ml), < NG, 0,0096 ± 0,0009; 0,0660 ± 0,0417; 0,1466 ± 0,0721; 0,4850 ± 0,2496 µg/ml), Nachkommen: ♂ AGD/KG ↓ (PND 23, ♀ keine Veränderung), keine statistisch signifikanten Veränderungen bei Implantationen/Wurf, Resorptionen/Wurf, % Würfe mit Resorptionen, Anzahl u. KG der lebenden Nachkommen, Geschlechterverhältnis, Testosteron im Serum ♂ Nachkommen nicht dosisabhängig ↓ (PND 23, stat. sign. nur bei 207 u. 1798 mg/kg KG)	Nakamura et al. 2015
Ratte, Hsd:Sprague Dawley^®^ SD^®^, 25 ♀	modifizierte Ein-Generationenstudie, GD 6–PND 91, 0, 3000, 10 000, 30 000 mg/kg Futter (Gestation: F0: 0, 205, 697, 2644 mg/kg KG u. d; F1: 0, 240, 825, 2760 mg/kg KG u. d), Reinheit > 99 %, Untersuchung GD 21 u. postnatal; Positivkontrolle: 0,05 mg Ethinylestradiol/kg Futter	**205/240 mg/kg KG**: **NOAEL** Maternal- u. Perinataltoxizität; **ab 697/825 mg/kg KG**: Muttertiere: F0 u. F1 KG-Zunahme ↓, Nachkommen: ♂ Balanopreputial-Separation verzögert (bei Bezug auf KG nicht, daher wahrscheinlich durch Wachstumsverzögerung verursacht), F1 ♂ KG/Wurf ↓ (PND 1 u. 4; F2 nur Trend statistisch signifikant, aber paarweiser Vergleich nicht); **bei 2644/2760 mg/kg KG**: Muttertiere: F0 Futteraufnahme ↑, Anzahl lebender Feten/Wurf (GD 21) ↓ (12,0 ± 0,4, Kontrolle: 13,6 ± 0,5, aber auch KG ↓ u. Corpora lutea/Muttertier ↓, d. h. auch Stresseffekt od. Effekt auf Fertilität möglich), Nachkommen: F1 ♀ KG/Wurf ↓ (PND 4, PND 1 nicht, F2 nur Trend statistisch signifikant, aber paarweiser Vergleich nicht), keine Veränderungen der Östrogen- u. Androgenrezeptor-vermittelten Entwicklungsmarker	NTP 2022 a; siehe. Abschnitt 5.5.1
Maus, VAF Crl: Swiss CD-1 (ICR)BR, 20 ♀ u. 40 ♀ Kontrolltiere	Reproduktionsstudie mit kontinuierlicher Verpaarung, 1 Wo vor Zusammensetzen u. insgesamt 98 d, 0, 12 500, 25 000, 50 000 mg/kg Futter (♀ Tiere: 0, 1900, 4100, 9500 mg/kg KG u. d), Reinheit > 99 %	**1900 mg/kg KG**: **NOAEL** Maternal- u. Perinataltoxizität; **ab 4100 mg/kg KG**: Muttertiere: KG transient ↓ (nach 2 u. 3 Wo), Futterverbrauch ↑ (errechnet pro Käfig), Anzahl lebender Nachkommen ↓ (ging einher mit verringertem KG der Elterntiere); **bei 9500 mg/kg KG**: Muttertiere: kumulative Tage bis zum Werfen für 2. u. 3. Wurf ↑, Nachkommen: KG ↓	NTP 1990; siehe. Abschnitt 5.5.1
Maus, BALB/c, k. A. zur Anzahl der Muttertiere (Histologie 5–7 Nachkommen/Dosis)	GD 0–PND 21, 0; 0,03; 0,212; 3 mg/kg KG u. d, oral mit Pipette, Vehikel: Maiskeimöl, Reinheit > 99 %, Untersuchung ♀ Nachkommen im Alter von 17–19 Wo	**bei 3 mg/kg KG**: Nachkommen ♀: Brustdrüse Dichte der Ducti ↑ im Vergleich zur Kontrolle u. zu Nulliparen, Zellproliferation des Ductusepithels ↑, Muttertiere u. Nachkommen: kein Effekt auf Überleben u. KG, viele Parameter gemessen, die entgegen der Meinung der Autoren nicht dosisabhängig verändert sind	LaPlante et al. 2018
Maus, BALB/c, 9–11 Muttertiere (je 1 Tier/Geschlecht u. Dosis u. Zeitpunkt untersucht)	GD 0–PND 21, 0; 0,03; 0,212; 3 mg/kg KG u. d, oral mit Pipette, Vehikel: Maiskeimöl mit Tocopherolzusatz, Reinheit > 99 %, Untersuchung ♂/♀ Nachkommen im Alter von 21 d (vor Pubertät), 32–35 d (Pubertät), 9–11 Wo (adult)	**bei 3 mg/kg KG**: Nachkommen ♀ u. ♂: Zellproliferation des Ductusepithels ↓ (gegenteiliger Effekt bei ♀ im Vergleich zu Studie von LaPlante et al. 2018, selbe Arbeitsgruppe), ♀: Brustdrüse Anzahl TEB ↑ (32–35 d), Größe der Alveolarknospen ↑ (9–11 Wo), ♂: Brustdrüse Wachstum u. Größe ↓, Muttertiere u. Nachkommen: kein Effekt auf Überleben u. kein dosisabhängiger Effekt auf KG, AGD/KG ohne Dosis-Wirkungs-Beziehung u. kein Effekt nach 9–11 Wo, viele Parameter gemessen, die entgegen der Meinung der Autoren nicht dosisabhängig verändert sind, die Autoren berichten über antiöstrogene (in der Publikation derselben Arbeitsgruppe LaPlante et al. 2018 wird von östrogener Aktivität berichtet) u. antiandrogene Aktivität	Matouskova et al. 2020

a) basierend auf den Daten aus einer Humanstudie mit dermaler Ganzkörperapplikation von 2 mg/cm^2^ einer Sonnencreme mit 10 % Benzophenon-3: 17 Frauen mit durchschnittlichem KG von 68 kg und applizierter Sonnencrememenge von 35 g (Janjua et al. 2008) entspricht 51,7 mg Benzophenon-3/kg KG und Tag

b) basierend auf den Daten unter ^a)^: höchster Wert von 187 ng/ml 4 h nach Applikation; 53 ng/ml 96 h nach Applikation (Janjua et al. 2008), d. h. die Mäuse waren geringer belastet

abzgl.: abzüglich; AGD: Anogenitalabstand; ALP: alkalische Phosphatase; ALT: Alaninaminotransferase; BP-3: Benzophenon-3; d: Tag; DHB: 2,4-Dihydroxybenzophenon; Esr1: Östrogenrezeptor α; Esr2: Östrogenrezeptor β; GD: Gestationstag; Gzmb: Granzym b; Icam-1: Intercellular Adhesion Molecule 1; Pgr: Progesteronrezeptor; PND: Postnataltag; TEB: terminale Endknospen (Terminal End Buds); TG: Test Guideline (Prüfrichtlinie); u: und

In einer Studie zur pränatalen Entwicklungstoxizität nach OECD-Prüfrichtlinie 414 an Wistar (Crl:WI(Han))-Ratten wurden 0, 40, 200 oder 1000 mg Benzophenon-3/kg KG und Tag per Gavage vom 6. bis zum 19. Gestationstag verabreicht. Bei 1000 mg/kg KG und Tag traten bei den Muttertieren mit Urin beflecktes Fell sowie transient erniedrigte Futteraufnahme und Körpergewichtszunahme auf. Die Feten wiesen bei dieser Dosis vermehrt skelettale Variationen auf (nicht vollständige Ossifikationen von Schädelknochen und der Halswirbelsäule, zusätzliche 14. Rippen; Gesamtzahl der Variationen statistisch signifikant erhöht). Teratogene Effekte wurden durch Benzophenon-3 nicht ausgelöst. Es ergab sich ein NOAEL für Maternal- und pränatale Entwicklungstoxizität von 200 mg/kg KG und Tag (BASF AG Experimental Toxicology and Ecology 2005; SCCP 2008; SCCS 2021).

Bei ICR-Mäusen, die vom 0. bis zum 6. Gestationstag per Gavage behandelt wurden, kam es ab der niedrigsten Dosis von 0,1 mg Benzophenon-3/kg KG und Tag in der Plazenta zu einer beginnenden Thrombose und Gewebsnekrose mit einer verstärkten Thrombozytenaggregation (k. A. von Inzidenzen u. Schweregraden). Bei 1000 mg Benzophenon-3/kg KG und Tag traten vermehrt fetale Verluste auf (Han et al. 2022). Zur beginnenden Thrombose und Gewebsnekrose in der Plazenta werden nur repräsentative histologische Gewebeschnitte gezeigt, eine quantitative Auswertung von Inzidenz und Schweregrad wird nicht vorgenommen. Es ist unklar, wie häufig derartige Veränderungen bei diesem Mäusestamm spontan auftreten. Die Effektdosis von 1000 mg/kg KG und Tag ist 100-mal so hoch wie die nächstniedrigere Dosis von 10 mg/kg KG und Tag, sodass hier die Angabe eines NOAEL für Entwicklungstoxizität nicht sinnvoll ist.

In einer Studie an Mäusen mit dermaler Applikation von 50 mg Benzophenon-3/kg KG vom 0. bis zum 6. Gestationstag, was einer Dosis für Menschen bei der Verwendung von Sonnencreme entspricht, wurde eine intrauterine Wachstumsrestriktion sowie eine Erhöhung des Prozentsatzes weiblicher Feten festgestellt (Santamaria et al. 2020). Da der Postimplantationsverlust nicht erhöht war, ist letzteres wahrscheinlich ein Zufallsbefund. Eine nachfolgende Studie derselben Arbeitsgruppe bestätigte die Befunde der intrauterinen Wachstumsrestriktion bei 50 mg/kg KG und Tag nach dermaler Applikation. In den Plazenten war die Genepression bei weiblichen Feten, jedoch nicht bei männlichen Feten, am 14. Gestationstag verändert. So waren die Genexpressionen von Pgr (Progesteronrezeptor), Icam1 (Intercellular Adhesion Molecule 1) und Gzmb (Granzym b) erniedrigt. Diese Rezeptoren sind bei plazentalen Funktionen wie dem hormonellen Signalling (Pgr), Zelladhäsion (Icam1) und dem Remodelling des Gewebes (Gzmb) beteiligt. Die Genexpression der beiden Östrogenrezeptoren blieb bei beiden Geschlechtern durch die Benzophenon-3-Gabe unverändert. Die Autoren postulieren eine Beeinträchtigung des fetalen Wachstums durch eine Störung der Progesteron-Signalübertragung. Progesteron ist für die Dezidualisierung und die Aufrechterhaltung der Schwangerschaft verantwortlich. Während der Plazentation schränkt Progesteron die übermäßige Invasion extravillärer Trophoblasten ein und stimuliert die Migration vaskulärer Endothelzellen, die für die Angiogenese wichtig ist (Fischer et al. 2024). Die Benzophenon-3-Konzentrationen im Serum der behandelten Mäuse lagen bei 22,4 ng/ml vier Stunden und bei 16,8 ng/ml acht Tage nach der letzten Applikation am 6. Gestationstag (Santamaria et al. 2020). Im Vergleich dazu wurden bei 17 Frauen in einer Probandenstudie mit dermaler Ganzkörperapplikation (durchschnittliches Körpergewicht der Frauen 68 kg, 35 g Sonnencreme mit 10 % Benzophenon-3) folgende Medianwerte im Plasma bestimmt: Der höchste Medianwert von 187 ng/ml wurde vier Stunden nach der Applikation gemessen, 96 Stunden nach der Applikation lag die Konzentration bei 53 ng/ml (Janjua et al. 2008). Demnach lagen die Messwerte bei den Frauen mindestens um das 3,2-Fache höher im Vergleich zu den weiblichen Mäusen. In den Studien von Santamaria et al. (2020) und Fischer et al. (2024) wurden zwei verschiedene Mausstämme gekreuzt und für einzelne Untersuchungen nur 3–4 Tiere (Santamaria et al. 2020) oder 5–6 Tiere (Fischer et al. 2024) eingesetzt. Zudem handelt es sich bei den untersuchten Parametern nicht um von der Prüfrichtlinie empfohlene Parameter; aufgrund der fehlenden Validierung ist die Aussagekraft der Parameter fraglich. Es ist nur eine Dosis eingesetzt worden, daher kann keine Aussage über die Dosis-Wirkungs-Beziehung getroffen werden. Insgesamt ist das Tiermodell in Frage zu stellen und die Daten werden als nicht belastbar angesehen. Des Weiteren sind in der Reproduktionsstudie mit kontinuierlicher Verpaarung an einem anderen Mäusestamm erst bei weitaus höheren oralen Dosierungen (Futtergabe) von 9500 mg/kg KG und Tag Wachstumsverzögerungen bei den Feten aufgetreten (NTP 1990). Auch wenn diese Applikationsart von der dermalen Gabe abweicht, weist dies darauf hin, dass permanente Wachstumsverzögerungen im niedrigen Dosisbereich (bei 50 mg/kg KG und Tag) nach dermaler Gabe nicht zu erwarten sind.

Benzophenon-3 zeigte in der im Abschnitt 5.5.1 beschriebenen modifizierten **Ein-Generationenstudie** an Sprague-Dawley-Ratten ab 697 (F0-Generation) bzw. 825 mg/kg KG und Tag (F1-Generation) eine verzögerte Körpergewichtszunahme bei den Muttertieren der F0- und der F1-Generation sowie Wachstumsverzögerungen bei den Nachkommen in der F1- und F2-Generation. Es wurden bis zur höchsten Dosis von 2644/2760 mg/kg KG und Tag keine Anzeichen einer östrogenen, androgenen oder antiandrogenen Wirkung festgestellt. So traten weder Veränderungen der androgen-vermittelten Entwicklungsmarker, noch histopathologische Veränderungen auf, die auf eine Störung der normalen Östrogen- oder Androgenrezeptor-vermittelten Entwicklung hindeuten (NTP 2022 a). Es ergibt sich ein NOAEL für Maternal- und Perinataltoxizität von 205/240 mg/kg KG und Tag.

In der Reproduktionstoxizitätsstudie mit kontinuierlicher Verpaarung an Mäusen kam es nach Gabe mit dem Futter ab 4100 mg/kg KG und Tag zu einer transienten Abnahme des maternalen Körpergewichts sowie zu einer erniedrigten Anzahl lebender Nachkommen (NTP 1990; siehe Abschnitt 5.5.1). Daraus ergibt sich ein NOAEL für Maternal- und Perinataltoxizität von 1900 mg/kg KG und Tag.

In zwei Untersuchungen derselben Arbeitsgruppe an BALB/c-Mäusen, die sich nur auf die Brustdrüsenentwicklung der Nachkommen fokussiert hatten, wurden die Muttertiere vom 0. bis zum 21. Postnataltag behandelt. Es wurden Veränderungen der Brustdrüse der Nachkommen gesehen, die zum Teil (Zellproliferation des Ductusepithels) entgegengesetzt und zum größten Teil nicht dosisabhängig waren (LaPlante et al. 2018; Matouskova et al. 2020). Es wurde nur eine geringe Anzahl an Tieren eingesetzt. Zudem sehen die Autoren Dosis-Wirkungs-Beziehungen, obwohl diese den Abbildungen nicht zu entnehmen sind.

### 5.6 Genotoxizität

#### 5.6.1 In vitro

Untersuchungen mit der Benzophenon-3-Charge, die auch in der Kanzerogenitätsstudie (siehe Abschnitt 5.7) eingesetzt wurde, waren zwischen 20 und 6000 µg/Platte in Salmonella typhimurium TA98 und TA100 sowie in Escherichia coli WP2 *uvr*A/pKM101 in An- und Abwesenheit von 10 % eines metabolischen Aktivierungssystems aus Rattenleber negativ (NTP 2020). 

Nach Angaben in ECHA (2013) war eine Studie von 1983 mit bis zu 1250 µg Benzophenon-3/Platte an Salmonella typhimurium TA98, TA100, TA1535, TA1537 und TA1538 und Escherichia coli WP2 und WP2 *uvrA* sowohl in An- als auch in Abwesenheit eines metabolischen Aktivierungssystems aus der Rattenleber nach OECD-Prüfrichtlinie 471 negativ. Oberhalb von 1250 µg Benzophenon-3/Platte zeigte sich in Vorversuchen Toxizität. In keiner der Platten wurde ein Anstieg der Anzahl an Revertanten beobachtet (ECHA 2013). Jedoch fehlen Tabellen mit Einzelergebnissen, sodass die Bewertung der Befunde nicht überprüft werden kann. 

In einer Untersuchung von 1991 an Salmonella typhimurium TA98, TA100, TA1535, TA1537 sowie Escherichia coli WP2 *uvr*A und einem metabolischen Aktivierungssystem aus Aroclor 1254-aktivierten Rattenlebern trat keine Mutagenität, jedoch mehr als 50%ige Zytotoxizität ab 625 µg Benzophenon-3/Platte bei TA1537 und 1250 µg/Platte bei TA1535 auf (ECHA 2013). Die Einzeldaten wurden berichtet.

In zwei unabhängigen Untersuchungen an Salmonella typhimurium TA98, TA100, TA1535, TA1537 war Benzophenon-3 bis 1000 µg/Platte in An- und Abwesenheit von 10 % S9-Mix aus Ratten- oder Hamsterleber negativ (French 1992).

In einem weiteren Versuch wurde Benzophenon-3 in Salmonella typhimurium TA97, TA98, TA100 und TA1535 bis 1000 µg/Platte in An- und Abwesenheit von 10 % oder 30 % S9-Mix aus Ratten- und Hamsterleber untersucht. Dabei zeigte sich eine leicht mutagene Wirkung in TA97 sowie TA100, jedoch nur bei Verwendung von 30 % S9-Mix aus Hamsterleber, nicht bei 10 % oder mit S9-Mix aus Rattenleber. Die Revertantenzahlen erreichten keine Verdopplung (French 1992). Aufgrund der weniger als verdoppelten Revertantenzahlen und des positiven Ergebnisses nur mit 30 % S9-Mix wird aus dieser Studie kein Hinweis auf eine mutagene Wirkung von Benzophenon-3 abgeleitet.

In Chinesischen-Hamster-Ovarienzellen (CHO-Zellen) induzierte Benzophenon-3 in zwei Ansätzen bei 5 bis 50 µg/ml Schwesterchromatidaustausche (SCE) in Anwesenheit von S9-Mix aus Rattenlebern. Der SCE-Test ohne metabolische Aktivierung war nicht statistisch signifikant im paarweisen Vergleich, jedoch zeigte sich ein konzentrationsabhängiger Trend (French 1992).

In derselben Zelllinie wurden Chromosomenaberrationen ebenfalls nur mit metabolischer Aktivierung, im ersten Ansatz bei 20 und 43 µg/ml, im zweiten bei 30 und 45 µg/ml, induziert. Die fehlende Konzentrationsabhängigkeit und das negative Ergebnis höherer Konzentrationen (≥ 60 µg/ml) liegt an der Präzipitation der Substanz oder Zytotoxizität (French 1992). 

Eine Untersuchung von 2005 auf strukturelle Chromosomenaberrationen in V79-Hamsterzellen mit bis zu 75 µM Benzophenon-3 war in An- und Abwesenheit von künstlichem UV-Licht als Aktivierungssystem in zwei verschiedenen Untersuchungen in Anlehnung an OECD-Prüfrichtlinie 473 negativ ohne Bestrahlung. Der mitotische Index war bei der höchsten Konzentration reduziert und es kam zur Präzipitation. Benzophenon-3 war somit mit bzw. ohne künstliches UV-Licht nicht klastogen bis zu zytotoxischen Konzentrationen. Die Positivkontrolle zeigte das erwartete Ergebnis (ECHA 2013; SCCP 2008). Jedoch fehlen Tabellen mit Einzelergebnissen, sodass die Bewertung der Befunde nicht überprüft werden kann.

Auch zwei HPRT-Tests an V79-Zellen des Chinesischen Hamsters nach OECD-Prüfrichtlinie 476 mit 5 bis 160 µg/ml verliefen negativ (ECHA 2013). Auch hier fehlen Tabellen mit Einzelergebnissen, sodass die Bewertung der Befunde nicht überprüft werden kann.

Benzophenon-3 zeigte in keiner der Tox21-Untersuchungen auf DNA-Schäden eine Aktivität (NTP 2020).

#### 5.6.2 In vivo

Eine Untersuchung auf somatische Mutationen und Rekombinationen (SMART) in Drosophila, die im Larvenstadium 72 Stunden lang Futter mit 0, 3000 oder 3500 ppm Benzophenon-3 erhielten, war negativ (NTP 2020; Robison et al. 1994). Es wurden keine Mutationen einschließlich Deletionen oder Rekombinationen hervorgerufen (Robison et al. 1994), wobei keine Einzeldaten berichtet werden.

Auch trat keine Induktion von Chromosomenaberrationen bei männlichen Sprague-Dawley-Ratten auf, die einmalig oder an fünf aufeinander folgenden Tagen bis zu 5000 mg Benzophenon-3/kg KG mit der Schlundsonde erhielten (NTP 2020).

In der oralen 13-Wochen-Studie an männlichen und weiblichen Mäusen trat bis zur höchsten Dosis von 13 937 bzw. 18 539 mg/kg KG und Tag kein Anstieg an Mikronuklei in normochromatischen Erythrozyten im Blut auf (French 1992).

#### 5.6.3 Fazit

Benzophenon-3 war in Untersuchungen nach OECD-Prüfrichtlinien an Bakterien überwiegend negativ. Die gering erhöhten (weniger als verdoppelten) Revertantenzahlen in TA97 und TA100 wurden nur in einer Studie und nur nach Zugabe von 30 % metabolischem Aktivierungssystem der Hamsterleber beobachtet, mit 10 % nicht. TA100 ist in anderen Studien negativ. Daher wird Benzophenon-3 insgesamt als nicht mutagen in Bakterien bewertet. Auch in Säugerzellen wurde keine mutagene Wirkung beobachtet. In CHO-Zellen verliefen Tests auf SCE und Chromosomenaberrationen in Anwesenheit eines metabolischen Aktivierungssystems positiv. Dieser Hinweis auf eine klastogene Wirkung bestätigte sich jedoch nicht in vivo. Benzophenon-3 führte bei männlichen Ratten nach Schlundsondengabe an einem oder fünf aufeinander folgenden Tagen nicht zu Chromosomenaberrationen am Knochenmark und in Erythrozyten von Mäusen nicht zu Mikronuklei nach 90-tägiger Gabe mit dem Futter. Benzophenon-3 war ebenfalls negativ in einer Untersuchung auf somatische Mutationen und Rekombinationen in Drosophila.

### 5.7 Kanzerogenität

In einer Kanzerogenitätsstudie mit B6C3F1-Mäusen erhielten männliche und weibliche Tiere mit dem Futter bis zu 1207 bzw. 1278 mg Benzophenon-3/kg KG und Tag. Bei dieser höchsten Dosis war die Körpergewichtszunahme um mehr als 10 % vermindert, was das Erreichen der maximal tolerierbaren Dosis anzeigt. Es traten keine erhöhten Tumorinzidenzen auf (NTP 2020). 

Bei männlichen und weiblichen Mäusen dieser Studie wurden vereinzelt histiozytäre Sarkome beobachtet, die eine geringe Inzidenz (max. 2/50) aufwiesen, nicht dosisabhängig waren und auch bei einem Kontrolltier beobachtet wurden. NTP (2020) und die Kommission bewerten diese daher als nicht substanzbedingt.

#### Kanzerogenitätsstudie an F1-Ratten mit Expositionsbeginn in utero

Wegen des weit verbreiteten Einsatzes von Benzophenon-3 in Körperpflegeprodukten kann es bereits während der Schwangerschaft zu einer Exposition des Ungeborenen kommen. Daher wurde eine Kanzerogenitätsstudie an trächtigen Harlan Sprague-Dawley-Ratten mit Expositionsbeginn ab dem 6. Trächtigkeitstag durchgeführt, während der Laktation erfolgte die Exposition weiter durch Futter mit 0, 1000, 3000 und 10 000 mg Benzophenon-3 pro kg Futter. Nach dem Absetzen wurden je 50 männliche und 50 weibliche F1-Tiere pro Dosisgruppe zwei Jahre lang weiter mit denselben Dosierungen im Futter exponiert, die höchste Dosis entsprach 585 bzw. 632 mg/kg KG und Tag für männliche bzw. weibliche Tiere. Bei exponierten weiblichen F1-Ratten trat eine erhöhte Inzidenz an Polypen im Stroma des Uterus, atypische Hyperplasie im Endometrium des Uterus, aber eine verminderte Zahl an Adenokarzinomen im Uterus in der mittleren Dosisgruppe und eine erhöhte Inzidenz an C-Zell-Adenomen der Schilddrüse auf, wobei letztere in der mittleren Dosis oberhalb der historischen Kontrollinzidenz, sonst innerhalb der historischen Kontrolle lagen. Bei männlichen F1-Ratten wurde bei einem Tier der niedrigen (58 mg/kg KG und Tag) und drei Tieren der mittleren Dosis (168 mg/kg KG und Tag) aus zwei verschiedenen Würfen ein malignes Meningeom im Gehirn und bei einem Tier im Rückenmark (dieses wies kein Meningeom im Gehirn auf) beobachtet (siehe Tabelle 6). Die Inzidenz historischer Kontrollen wird mit 0/340 angegeben (NTP 2020).

Die Polypen im Stroma des Uterus sind nicht-neoplastische Befunde und werden von der Kommission als substanzbedingt, aber nicht einstufungsrelevant bewertet. Die atypischen Hyperplasien des Endometriums des Uterus sind eine präkanzerogene Veränderung, jedoch nahm gleichzeitig die Inzidenz an Adenokarzinomem im Uterus dieser Dosisgruppe ab.

Die bei weiblichen Ratten beobachteten C-Zell-Adenome in der Schilddrüse nahmen zwar in der Inzidenz dosisabhängig zu, liegen aber in einem von der Kommission als normal bewerteten Bereich und werden damit ebenfalls als nicht einstufungsrelevant bewertet.

Bei den malignen Meningeomen handelt es sich um eine selten spontan vorkommende Tumorart, die nach Angaben der Studienautoren in historischen Kontrolldaten über alle Applikationswege eine Inzidenz von 0/340 aufweist. Daher werden der eine Tumor in der niedrigen und die drei Tumoren im Gehirn und ein Tumor im Rückenmark in der mittleren Dosisgruppe männlicher Ratten als auffällige Häufung gewertet, zumal die betroffenen Tiere in der mittleren Dosis aus zwei verschiedenen Würfen stammten. Von den vier betroffenen Tieren mit Tumor im Gehirn sind drei vor Studienende gestorben. Eine mögliche Erklärung für den vorzeitigen Tod ist, dass die Raumforderung des Tumors bei seinem Wachstum lebenswichtige Areale bedrängt hat, für die es unter dem Schädelknochen oder im Rückenmarkkanal keinen Raum zum Ausweichen gibt. Es ist kein Mechanismus bekannt, durch den die malignen Meningeome im Gehirn bzw. Rückenmark männlicher Ratten substanzbedingt hervorgerufen werden könnten und der erklärt, warum dies nur bei männlichen Tieren und nur bei Ratten erfolgt. Da Meningeome in der Gehirnhaut lokalisiert sind, ist für eine mögliche Auslösung durch eine Substanz keine Gehirngängigkeit derselben notwendig. Maligne Meningeome zeigen eine abnehmende Expression von Hormonrezeptoren mit zunehmenden Tumorgrad. Diese Tumorart kann durch Bestrahlungen ausgelöst werden (siehe Abschnitt 2.2). Bestrahlungen der Tiere fanden in der NTP-Studie nicht statt (NTP 2020) und können daher kein Auslöser dieser Tumorart sein.

NTP hat von 2010 bis 2011 vier Studien mit Harlan Sprague-Dawley-Ratten gestartet, bei denen die Muttertiere die entsprechende Substanz, einmal Benzophenon-3, mit dem Futter bekamen und die Kanzerogenitätsstudie mit in utero exponierten F1-Tieren erfolgte. In diesen vier Studien trat bei einem männlichen (1/199) und einem weiblichen (1/200) Kontrolltier (zwei verschiedene Studien) ein malignes Meningeom im Gehirn, in der dritten Studie ein benignes Meningeom bei einem männlichen Kontrolltier im Gehirn auf (NTP 2022 b). Nach dieser Zusammenstellung ist das spontane Auftreten von Meningeomen nicht ganz so selten oder wird durch den Expositionsbeginn in utero gefördert.

Bei den fünf beobachteten malignen Meningeomen bei Benzophenon-3 exponierten männlichen Ratten handelt es sich um eine seltene Tumorart an einem sehr sensiblen Organ. Da keine Kanzerogenitätsstudie an Ratten mit Expositionsbeginn im Erwachsenenalter vorliegt, kann keine Aussage getroffen werden, ob diese Tumorart durch den Expositionsbeginn in utero begünstigt wurde. Somit wird der Befund berücksichtigt, auch wenn er nicht statistisch signifikant, nicht dosisabhängig, eventuell durch den Expositionsbeginn in utero begünstigt und fraglich substanzbedingt ist.

**Tab. 6:** Spezielle Kanzerogenitätsstudie mit Benzophenon-3 an F1-Ratten mit Expositionsbeginn am 6. Trächtigkeitstag in utero

Autor:	NTP 2020				
Stoff:	Benzophenon-3 (> 99 % rein)				
Spezies:	**Ratte**, Harlan Sprague Dawley, je 50 F1 ♂, 50 F1 ♀, **die bereits in utero exponiert waren**^#^				
Applikation:	in utero ab GD 6 bis zum Absetzen im Futter der Muttertiere, danach im Futter der F1-Tiere				
Konzentration:	0, 1000, 3000, 10 000 mg/kg Futter (für Muttertiere und F1-Tiere)(0, 58/60, 168/180, 585/632 mg/kg KG u. d für ♂/♀)				
Dauer:	2 Jahre, 7 d/Wo				
Toxizität:	♀: ab 60 mg/kg KG: fokale Hyperplasie Nierenkortex; ♂: 585 mg/kg KG: KG ↓				
		**Expositionskonzentration [mg/kg KG u. d] ♂/♀**			
		**0/0**	**58/60**	**168/180**	**585/632**
Überlebende	♂	30/50 (60 %)	29/50 (58 %)	24/50 (48 %)	33/50 (66 %)
	♀	30/50 (60 %)	33/50 (66 %)	34/50 (68 %)	26/50 (52 %)
**Tumoren und Präneoplasien**					
**Gehirn:**					
malignes Meningeom^a)^	♂	0/50 (0 %)	1/50 (2 %)	3/50 (6 %)	0/50 (0 %)
Inzidenz pro Wurf	♂	0/29 (0 %)	1/25 (4 %)	2/25 (8 %)	0/28 (0 %)
adjustierte Rate^b)^	♂	0 %	2,3 %	7 %	0 %
terminale Inzidenz^c)^	♂	0/30 (0 %)	0/29 (0 %)	1/24 (4 %)	0/33 (0 %)
erstes Auftreten [Tag]		–	162	430	–
Rao-Scott-adjustierter Poly-3-Test		P = 0,583N	P = 0,706	P = 0,291	n. b.
**Rückenmark**					
malignes Meningeom	♂	0/50 (0 %)	0/50 (0 %)	1/50 (2 %)	0/50 (0 %)
**Schilddrüse:**					
C-Zell-Adenom^d)^	♂	7/50 (14 %)	10/50 (20 %)	8/50 (16 %)	8/50 (16 %)
	♀	5/50 (10 %)	11/50 (22 %)	17/50 (34 %)	10/50 (20 %)
Inzidenz pro Wurf	♀	5/29 (17 %)	10/25 (40 %)	15/25 (60 %)	9/29 (31 %)
adjustierte Rate^e)^	♀	12,4 %	25,1 %	37,2 %	24,3 %
terminale Inzidenz^f)^	♀	3/30 (10 %)	9/33 (27 %)	12/34 (35 %)	6/26 (23 %)
erstes Auftreten [Tag]	♀	582	529	540	581
C-Zell-Karzinom^g)^	♀	1/50 (2 %)	1/50 (2 %)	0/50 (0 %)	1/50 (2 %)
Inzidenz pro Wurf	♀	1/29 (3 %)	1/25 (4 %)	0/25 (0 %)	1/29 (3 %)
adjustierte Rate^e)^	♀	2,6 %	2,3 %	0 %	2,5 %
terminale Inzidenz^f)^	♀	1/30 (3 %)	1/33 (3 %)	0/34 (0 %)	1/26 (4 %)
erstes Auftreten [Tag]	♀	730	730	–	730
**Nebenniere**					
Cortex, fokale Hypertrophie	♀	24/50 (48 %) (2,0)^h)^	42/50** (84 %) (1,8)^h)^	39/50* (78 %) (1,6)^h)^	27/50 (54 %) (1,7)^h)^
**Uterus**					
Polypen Stroma	♀	8/50 (16 %)	15/50 (30 %)	18/50 (36 %)	10/50 (20 %)
atypische Hyperplasie Endometrium	♀	9/50 (18 %)	14/50 (28 %)	19/50 (38 %)*	14/50 (28 %)
Adenokarzinom	♀	5/50 (10 %)	3/50 (6 %)	0/50 (0 %)	4/50 (8 %)

# Ratten bereits in utero exponiert, wobei die Muttertiere ab der mittleren Dosisgruppe während Trächtigkeit und Laktation leicht verminderte KG-Zunahmen aufwiesen, die jedoch so gering waren, dass keine Effekte auf die Nachkommen angenommen wurden; nach dem Absetzen erhielten diese F1-Tiere weiterhin 2 Jahre lang Futter mit den entsprechenden Dosierungen 0, 1000, 3000, 10 000 mg/kg Futter;

d: Tag; GD: Gestationstag; n. b.: nicht berechenbar; Wo: Woche

* p ≤ 0,05

** p ≤ 0,01

a) Historische Kontrolle alle Applikationsrouten in 2-Jahre-Studien nach NTP 2020: 0/340; bei Expositionsbeginn in utero (4 Studien): 1/199 (2022 b)

b) Poly-3 bestimmte erwartete Inzidenz nach Adjustierung von zwischenzeitlicher Mortalität

c) Beobachtete Inzidenz zum Studienende

d) Historische Kontrolle alle Applikationsrouten in 2-Jahre-Studien nach NTP 2020: 38/339 (11,85 % ± 7,01 %); Bereich: 4 % bis 22 %

e) Poly-3 bestimmte erwartete Inzidenz nach Adjustierung für zwischenzeitliche Mortalität

f) Inzidenz zum Studienende

g) Historische Kontrolle alle Applikationsrouten in 2-Jahre-Studien nach NTP 2020: 4/339 (1,33 % ± 1,63 %); Bereich: 0 % bis 4 %

h) Schweregrad des Effektes: 1 = minimal, 2 = leicht, 3 = mittel, 4 = schwer

## 6 Bewertung

Benzophenon-3 wirkt beim Menschen als Kontaktallergen. Durch Belichtung können verstärkte kontaktallergische und photokontaktallergische Reaktionen auftreten. Ein weiterer kritischer Befund war in einer oralen Kanzerogenitätsstudie mit Ratten und **in utero**-Expositionsbeginn das Auftreten von malignen Meningeomen im Gehirn oder Rückenmark männlicher F1-Ratten, deren Entstehungsmechanismus unklar ist.

**Krebserzeugende Wirkung. **Eine Kanzerogenitätsstudie mit B6C3F1-Mäusen führte bis zu 1278 mg Benzophenon-3/kg KG und Tag im Futter zu keiner erhöhten Tumorinzidenz.

Bei Sprague-Dawley-Ratten, die bereits ab dem 6. Trächtigkeitstag in utero exponiert waren und mit denen eine Kanzerogenitätsstudie durchgeführt wurde, traten bei weiblichen F1-Tieren eine erhöhte Inzidenz an C-Zell-Adenomen der Schilddrüse (in der mittleren Dosis oberhalb der historischen Kontrollinzidenz, sonst innerhalb der historischen Kontrolle liegend) und Polypen im Stroma des Uterus auf. Bei männlichen F1-Tieren wurde ein malignes Meningeom bei einem Tier der niedrigen (58 mg/kg KG und Tag) und drei Tieren der mittleren Dosis (168 mg/kg KG und Tag) aus zwei verschiedenen Würfen im Gehirn und bei einem weiteren Tier dieser Dosisgruppe im Rückenmark beobachtet (NTP 2020). Diese Tumoren sind sehr selten, traten jedoch in Parallelstudien mit Expositionsbeginn in utero mit demselben Rattenstamm auch bei einem männlichen und einem weiblichen Kontrolltier auf (NTP 2022 b). Es ist kein Mechanismus bekannt, durch den diese Tumoren bei männlichen Ratten durch Benzophenon-3 hervorgerufen werden könnten. Daher kann keine Aussage über deren mögliche Geschlechts- und Speziesspezifität oder Humanrelevanz getroffen werden. Meningeome treten bei Frauen ca. zwei- bis dreifach so häufig auf wie bei Männern und ein hormoneller Einfluss ist wahrscheinlich. Auch ist unklar, in wieweit die Entwicklung dieser Tumorart durch den Expositionsbeginn der Ratten in der sehr sensiblen Entwicklungsphase in utero begünstigt wird, da keine Studie mit Expositionsbeginn ab dem Erwachsenenalter vorliegt. Die Relevanz dieser Tumoren aus einer In-utero-Exposition für den Arbeitsplatz ist nicht bekannt.

Benzophenon-3 wirkt nicht genotoxisch. Es kann aufgrund der vorliegenden Daten nicht entschieden werden, ob die fünf bei männlichen Ratten beobachteten Meningeome durch die Exposition während der sehr sensiblen Entwicklungsphase in utero und substanzbedingt hervorgerufen wurden. Daher wird Benzophenon-3 in die Kategorie 3 für Kanzerogene eingestuft.

**Keimzellmutagene Wirkung. **Benzophenon-3 wirkte nicht mutagen in Bakterien und Säugerzellen. In CHO-Zellen kam es zur Induktion von SCE und Chromosomenaberrationen. Dieser Hinweis auf eine klastogene Wirkung bestätigte sich jedoch nicht in vivo. Benzophenon-3 führte nach Schlundsondengabe an einem oder fünf aufeinander folgenden Tagen nicht zu Chromosomenaberrationen am Knochenmark männlicher Ratten und nach 90-tägiger Gabe mit dem Futter nicht zu Mikronuklei in Erythrozyten von Mäusen. Ein Test auf somatische Mutationen und Rekombinationen in Drosophila war ebenfalls negativ. Untersuchungen an Keimzellen oder ein Strukturverdacht liegen nicht vor. Daher erfolgt keine Einstufung in eine Kategorie für Keimzellmutagene.

**MAK-Wert und Spitzenbegrenzung. **Es liegen keine Studien mit wiederholter Inhalation von Benzophenon-3 vor. In einer Kanzerogenitätsstudie traten nach Exposition in utero ab der niedrigsten Dosis von 58 mg/kg KG und Tag mit dem Futter bei männlichen Ratten in geringer Inzidenz maligne Meningeome auf (siehe Abschnitt 5.7). Die Relevanz dieser Tumoren aus einer In-utero-Exposition für den Arbeitsplatz ist nicht bekannt. Aufgrund einer fehlenden Dosis ohne Effekt für eine mögliche Tumorinduktion wird für Benzophenon-3 kein MAK-Wert abgeleitet. Eine Spitzenbegrenzung entfällt.

**Fruchtschädigende Wirkung. **Aus den epidemiologischen Studien, in denen die Konzentrationen von Benzophenon-3 im Urin der untersuchten Frauen über der Hintergrundbelastung lagen, lassen sich Hinweise auf eine Assoziation zwischen erhöhten maternalen Benzophenon-3-Konzentrationen im Urin und einem erhöhten Geburtsgewicht bei Jungen (Philippat et al. 2012, 2019) sowie einem erhöhten Gestationsalter (Aker et al. 2019) ableiten.

In einer Studie zur pränatalen Entwicklungstoxizität nach OECD-Prüfrichtlinie 414 an Wistar (Crl:WI(Han))-Ratten mit Gavagegabe vom 6. bis zum 19. Gestationstag kam es bei 1000 mg/kg KG und Tag bei gleichzeitiger Maternaltoxizität (mit Urin beflecktes Fell, erniedrigte Futteraufnahme und Körpergewichtszunahme) bei den Feten vermehrt zu skelettalen Variationen (nicht vollständige Ossifikationen von Schädelknochen und der Halswirbelsäule, zusätzliche 14. Rippen; Gesamtzahl der Variationen erhöht). Teratogene Effekte wurden durch Benzophenon-3 nicht ausgelöst. Der NOAEL für maternale und pränatale Entwicklungstoxizität liegt bei 200 mg/kg KG und Tag (BASF AG Experimental Toxicology and Ecology 2005; SCCP 2008; SCCS 2021). Bei ICR-Mäusen kam es nach Gavagegabe vom 0. bis zum 6. Gestationstag ab der niedrigsten Dosis von 0,1 mg Benzophenon-3/kg KG und Tag in der Plazenta zu einer beginnenden Thrombose und Gewebsnekrose mit einer verstärkten Thrombozytenaggregation ohne genauere Angaben von Inzidenz und Schweregrad. Bei 1000 mg Benzophenon-3/kg KG und Tag traten vermehrt fetale Verluste auf (Han et al. 2022). Es ist unklar, wie häufig Thrombose und Gewebsnekrose der Plazenta bei diesem Mäusestamm spontan auftreten. Die Effektdosis für fetale Verluste von 1000 mg/kg KG und Tag ist 100-mal so hoch wie die nächstniedrige Dosis von 10 mg/kg KG und Tag, sodass hier die Angabe eines NOAEL für Entwicklungstoxizität nicht sinnvoll ist.

Auch in einer modifizierten Ein-Generationen-Futterstudie an Sprague-Dawley-Ratten traten ab 697 (F0-Generation) bzw. 825 mg/kg KG und Tag (F1-Generation) Wachstumsverzögerungen bei den Nachkommen in der F1- und F2-Generation auf bei gleichzeitig verzögerter Körpergewichtszunahme bei den Muttertieren der F0- und der F1-Generation (NTP 2022 a). Der NOAEL für Maternal- und Perinataltoxizität liegt bei 205/240 mg/kg KG und Tag. Eine Reproduktionstoxizitätsstudie mit kontinuierlicher Verpaarung von Mäusen ließ bei Futtergabe von Benzophenon-3 ab 4100 mg/kg KG und Tag eine transiente Abnahme des maternalen Körpergewichts sowie eine erniedrigte Anzahl lebender Nachkommen erkennen (NTP 1990; siehe Abschnitt 5.5.1). Der NOAEL für Maternal- und Perinataltoxizität liegt bei 1900 mg/kg KG und Tag.

Da kein MAK-Wert für Benzophenon-3 abgeleitet wird entfällt die Zuordnung zu einer Risiko-basierten Schwangerschaftsgruppe. Aus der beschriebenen Datenlage zu Benzophenon-3 ergibt sich kein Anlass, den Stoff der Schwangerschaftsgruppe B (Verdacht) zuzuordnen.

**Hautresorption. **Es wurde eine dermale Resorption beim Menschen aus Sonnencremes nachgewiesen, jedoch kann aus den vorliegenden Untersuchungen kein Flux berechnet werden (siehe Abschnitt 3.1.2). Bei auf den ganzen Körper applizierter Sonnenschutzlotion mit 4 % Benzophenon-3 wurden ca. 15 mg resorbiert, was bei 70 kg Körpergewicht ca. 0,2 mg/kg KG entspräche. Bei 2500 cm^2^ exponierter Hautfläche (Hände, Unterarme und Gesicht) wäre die resorbierte Dosis 0,025 mg/kg KG, bei wiederholter Applikation aber deutlich höher (siehe Abschnitt 3.1.2). In einer In-vitro-Studie mit Humanhaut betrug der Flux 2 µg/cm^2^ und Stunde bei 20%iger Resorption (durch und in die Haut). Bei 2500 cm^2^ exponierter Hautfläche (Hände, Unterarme und Gesicht) und einer Stunde Exposition wäre die resorbierte Dosis 0,071 mg/kg KG.

Für eine dermale Belastung am Arbeitsplatz ohne Anwendung als Sonnenschutzmittel errechnen sich aus dem niedrigsten Flux mit einer ethanolischen Lösung an Ratten von 0,347 µg/cm^2^ und Stunde (El Dareer et al. 1986) und aus dem höchsten Flux aus Paraffinöl bei Ratten von 7 µg/cm^2^ (Mutlu et al. 2020) bei den Standardbedingungen einer einstündigen Exposition von 2000 cm^2^ Haut (Hände, Unterarme) Belastungen mit 694 bzw. 14 000 µg Benzophenon-3.

Bei 13-wöchiger dermaler Gabe an Mäuse war die Spermienzahl in den Nebenhoden ab 22,75 mg/kg KG verringert, umgerechnet in einen NAEL (1:3) erhält man 7,6 mg/kg KG. Die Resorption aus einer Lotion betrug ca. 50 %, also 3,8 mg/kg KG bei der Maus. Dieser Wert allometrisch für den Menschen (1:7) umgerechnet ergibt 0,54 mg/kg KG. Eine Zeitextrapolation ist nicht notwendig, da es sich um Spermieneffekte handelt. Aus der Extrapolation vom Tier auf den Menschen (1:2) ergibt sich eine systemisch tolerable Dosis von 0,27 mg/kg KG.

Beim Szenario von 2500 cm^2^ gegen Sonnencreme am Arbeitsplatz exponierter Haut (Hände, Unterarme und Gesicht) beträgt die Aufnahme ca. 0,25 mg/kg KG, und kann bei wiederholter Applikation ca. dreimal so hoch werden. Aus der In-vitro-Studie mit Humanhaut ergibt sich eine Dosis von 0,071 mg/kg KG (28 % der tolerablen Dosis). Bei dem Szenario ohne Anwendung in Sonnenschutzmitteln beträgt die maximale Dosis 14 mg, bezogen auf 70 kg Körpergewicht 0,2 mg/kg KG und läge damit im Bereich der systemisch tolerablen Dosis. Dieser Wert ist sehr konservativ, weil der Flux durch die Rattenhaut höher ist als der Flux durch Humanhaut. 

Aus allen drei Berechnungswegen ergibt sich, dass Benzophenon-3 mit „H“ markiert wird.

**Sensibilisierende Wirkung. **Die vorliegenden Ergebnisse aus experimentellen Untersuchungen am Meerschweinchen sind negativ, jedoch wegen Abweichungen von den Prüfrichtlinien oder unvollständiger Dokumentation nicht eindeutig zu bewerten. Ein Local Lymph Node Assay an der Maus verlief negativ. Das Gesamtergebnis aus nicht-tierbasierten Alternativverfahren zur Untersuchung der hautsensibilisierenden Wirkung war positiv. In entsprechenden Photosensibilisierungstests ergab die Prüfung von Benzophenon-3 kein eindeutiges Ergebnis. 

Beim Menschen wurden kontaktallergische und photokontaktallergische Reaktionen beschrieben. Aufgrund der kontaktallergenen Wirkung beim Menschen wird Benzophenon-3 mit „Sh“ markiert. Es ist zu beachten, dass durch Belichtung verstärkte Reaktionen auftreten können. Da Benzophenon-3 jedoch nicht ausschließlich als Photoallergen wirkt, erfolgt keine Markierung mit „SP“. 

Zur Sensibilisierung an den Atemwegen liegen keine Daten vor, es erfolgt daher keine Markierung mit „Sa“.
